# Pervasive compartment‐specific regulation of gene expression during homeostatic synaptic scaling

**DOI:** 10.15252/embr.202052094

**Published:** 2021-08-16

**Authors:** David Colameo, Marek Rajman, Michael Soutschek, Silvia Bicker, Lukas von Ziegler, Johannes Bohacek, Jochen Winterer, Pierre‐Luc Germain, Christoph Dieterich, Gerhard Schratt

**Affiliations:** ^1^ Laboratory of Systems Neuroscience Institute for Neuroscience Department of Health Science and Technology Swiss Federal Institute of Technology ETH Zurich Switzerland; ^2^ Neuroscience Center Zurich ETH Zurich and University of Zurich Zurich Switzerland; ^3^ Institute for Physiological Chemistry Biochemical‐Pharmacological Center Marburg Philipps‐University of Marburg Marburg Germany; ^4^ Laboratory of Behavioural and Molecular Neuroscience Institute for Neuroscience Department of Health Science and Technology Swiss Federal Institute of Technology ETH Zurich Switzerland; ^5^ Institute for Neuroscience Department of Health Science and Technology Swiss Federal Institute of Technology ETH Zurich Switzerland; ^6^ Laboratory of Statistical Bioinformatics Department of Molecular Life Sciences University of Zürich Zurich Switzerland; ^7^ Section of Bioinformatics and Systems Cardiology Department of Internal Medicine III and Klaus Tschira Institute for Integrative Computational Cardiology University of Heidelberg Heidelberg Germany; ^8^ Present address: Neuroscience Therapeutic Area UCB Pharma Braine l'Alleud Belgium

**Keywords:** cellular compartment, homeostatic plasticity, local translation, microRNA, synaptic scaling, Methods & Resources, Neuroscience

## Abstract

Synaptic scaling is a form of homeostatic plasticity which allows neurons to adjust their action potential firing rate in response to chronic alterations in neural activity. Synaptic scaling requires profound changes in gene expression, but the relative contribution of local and cell‐wide mechanisms is controversial. Here we perform a comprehensive multi‐omics characterization of the somatic and process compartments of primary rat hippocampal neurons during synaptic scaling. We uncover both highly compartment‐specific and correlating changes in the neuronal transcriptome and proteome. Whereas downregulation of crucial regulators of neuronal excitability occurs primarily in the somatic compartment, structural components of excitatory postsynapses are mostly downregulated in processes. Local inhibition of protein synthesis in processes during scaling is confirmed for candidate synaptic proteins. Motif analysis further suggests an important role for trans‐acting post‐transcriptional regulators, including RNA‐binding proteins and microRNAs, in the local regulation of the corresponding mRNAs. Altogether, our study indicates that, during synaptic scaling, compartmentalized gene expression changes might co‐exist with neuron‐wide mechanisms to allow synaptic computation and homeostasis.

## Graphical abstract

Downscaling of excitatory synapses in response to high activity involves compartment‐specific changes in the neuron's transcriptome and proteome. This study supports the view that local gene regulation is important in both Hebbian and homeostatic forms of synaptic plasticity.

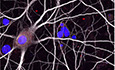

## Introduction

Synaptic scaling is an important cellular mechanism that keeps excitatory synaptic strength in a physiological range in response to chronic (> 6 h) changes in synaptic input (Turrigiano, 2008). In the mammalian brain, synaptic scaling plays important roles during neural circuit development (e.g. activity‐dependent development of the visual and barrel cortex) (Desai *et al*, 2002; Glazewski *et al*, 2017) and in cognition in the adult (e.g. memory consolidation during sleep) (Tononi & Cirelli, 2014; Diering *et al*, 2017). Moreover, defects in synaptic scaling have been associated with the pathophysiology of several neurological diseases, e.g. autism (Rett syndrome), mental retardation (e.g. Fragile‐X Syndrome) (Ramocki & Zoghbi, 2008), epilepsy (Swann & Rho, 2014) and mood disorders (Kavalali & Monteggia, 2012). Mechanistically, synaptic scaling involves mainly two components (Turrigiano, 2008). First, a sensing mechanism for the detection of changes in neuronal firing rates, such as intracellular calcium‐dependent pathways. Second, executing mechanisms which bring about changes in AMPA‐ and NMDA‐type glutamate receptor function at excitatory synapses. Recent evidence suggests that synaptic scaling requires changes in gene expression, both at the level of mRNA transcription (Ibata *et al*, 2008; Schaukowitch *et al*, 2017) and translation (Goold & Nicoll, 2010; Schanzenbacher *et al*, 2016; Schanzenbacher *et al*, 2018; Dorrbaum *et al*, 2020). This leads to a remodelling of the synaptic proteome, in particular by synthesis/degradation of neurotransmitter receptors, presynaptic proteins and components of the calcium‐dependent signalling pathways (Schanzenbacher *et al*, 2016; Rajman *et al*, 2017; Dorrbaum *et al*, 2020). The molecular mechanisms underlying these orchestrated changes in *de novo* protein synthesis, however, are only poorly understood. In hippocampal neurons, specific mRNAs are locally translated in the synapto‐dendritic compartment, which provides neurons with a means to regulate the activity of individual synapses or at least dendritic branches (Martin & Ephrussi, 2009). Interestingly, in contrast to classical Hebbian synaptic plasticity, homeostatic plasticity was initially believed to operate exclusively at a global level, either at the level of entire neurons or networks (Ibata *et al*, 2008). However, theoretical considerations in the past challenged this view and suggested an important contribution of local mechanisms (e.g. operating at the level of individual dendritic segments) to synaptic scaling (Rabinowitch & Segev, 2008). This view is supported by experimental results from cultured hippocampal neurons, which show local dendritic regulation of retinoic acid receptor signalling (Aoto *et al*, 2008) and AMPA‐type glutamate receptor synthesis (Sutton *et al*, 2004; Sutton *et al*, 2006) in response to synaptic scaling up induced by chronic activity blockade. In contrast, whether synaptic downscaling in response to chronically elevated activity similarly involves local regulation of gene expression has not been addressed. More generally, compartmentalized changes in gene expression and their regulatory mechanisms during homeostatic plasticity or any other form of synaptic plasticity have not been comprehensively assessed on a more general level using multi‐omics approaches.

## Results

**Figure 1:**
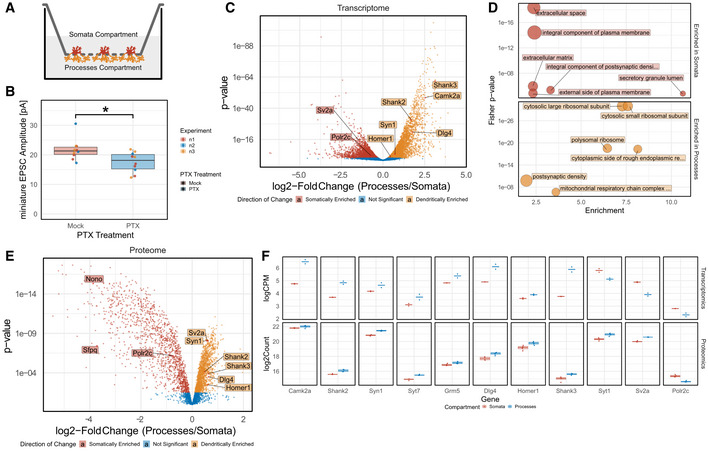
Compartment‐specific localization of neuronal transcripts and proteins Schematic of the workflow for the culture of primary hippocampal neurons and 48 h of PTX treatment.Quantification of miniature EPSC amplitudes in mock‐ and PTX‐treated hippocampal neurons. (*n* = 10 neurons per condition from three independent experiments, independent two‐sample Wilcoxon–Mann–Whitney *U*‐test, *P < 0.05).Volcano plots demonstrating enrichment of transcripts in either the somatic (red) or process (yellow) compartment with representative genes highlighted.Top Gene Ontology Pathway Analysis for transcripts enriched in the somatic and process compartment.Volcano plots demonstrating enrichment of proteins in either the somatic (red) or process (yellow) compartment with representative genes highlighted.Quantification of the highlighted genes enriched in either the somata or processes at the transcript or protein level. (Upper row: Scatterplots of transcript level; *n* = 2; crossbar represents mean. Lower row: Boxplot of protein levels; *n* = 4). Boxplots: central line: median; box: 25^th^ to 75^th^ percentile; whiskers: until last data point within 1.5× interquartile range (IQR).Linear model was used to fit transcriptome and proteome data and subsequently contrasted using likelihood ratio testing (transcriptome) or empirical Bayes statistics (proteome) for compartment effects.

**Figure EV1:**
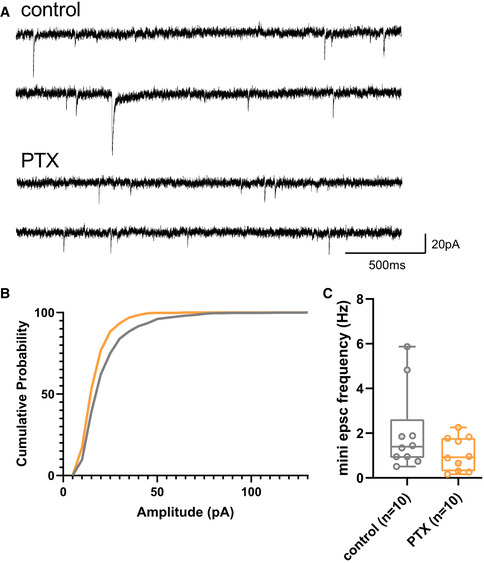
Additional data on electrophysiological experiments Example traces of a mock‐ and PTX‐treated neuron.Cumulative distribution of all miniature EPSC events of mock‐ (grey line) and PTX‐treated (orange line) neurons.Quantification of miniature EPSC frequency (Hz) presented as a boxplot (*n* = 10 neurons from three independent biological replicates). Boxplots: central line: median; box: 25^th^ to 75^th^ percentile; whiskers: until last data point within 1.5× interquartile range (IQR).

**Figure EV2:**
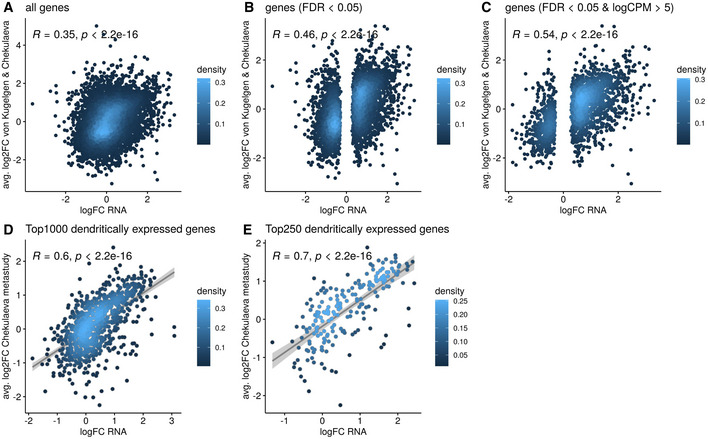
Subsumption of compartment‐specific gene location with a recent meta‐study under basal conditions Pearson's correlation of RNA logFC (Processes vs Somata) under basal conditions to the average of significant log_2_ enrichments of genes as of obtained by (von Kugelgen & Chekulaeva, 2020) from 11 high coverage datasets describing neuronal localization. The statistical significance of Pearson correlations was calculated in the standard fashion, i.e. by testing against a null hypothesis of *r* = 0 using a *t*‐distribution with *N*‐2 degrees of freedom.
ACorrelation with all genes detected in our study.BSubset of significantly enriched or depleted genes in the two compartments (FDR < 0.05).CSubset of significantly changing and highly expressed genes in the two compartments (FDR < 0.05 and logCPM > 5).D, ELocal enrichment correlation between a subset of the most abundant genes detected in neurites as classified by Kugelgen and Chekulaeva (2020) to genes detected in the compartment sequencing. Regression lines indicate fitted linear models, with the light grey shaded areas depicting the 95% confidence interval. Data information: Coloured is in each case the X‐Y density.

In this study, we interrogated compartmentalized gene expression in rat hippocampal neurons undergoing homeostatic plasticity using a multi‐omics approach. Therefore, we combined two *in vitro* model systems that we had previously established. First, a compartmentalized primary rat hippocampal neuron culture system, whereby neurons are plated on the upper side of filter inserts with small pore size (1 μm) that selectively allow the growth of neuronal processes (axons, dendrites), but not somata, to the lower side of the inserts (Fig 1A) (Bicker *et al*, 2013). Second, a pharmacological stimulation protocol (48 h) treatment with the GABA‐A receptor antagonist picrotoxin (PTX) that induces a robust and chronic increase in network activity followed by downscaling of excitatory synaptic strength (Fiore *et al*, 2014). Using patch‐clamp recordings, we detected a significant reduction in amplitude of miniature excitatory postsynaptic currents (mEPSC) in hippocampal pyramidal neurons 48 h post PTX treatment, demonstrating robust downscaling of excitatory synapses (Figs 1B and EV1A–C). To validate the compartmentalized culture system, we first performed a comparative transcriptomic analysis of the somatic and process compartment in unstimulated neurons using ribosome depletion RNA sequencing (ribo(−)RNAseq). Thereby, we identified 1,231/1,673 genes that were significantly enriched with a logFC of greater than ± 1 in the somatic/process compartment, respectively (Fig 1C). As expected, many well‐known dendritically and axonally localized mRNAs, e.g. Camk2a, Shank2/3, Homer1, Dlg4 and Syn1, were significantly enriched in the process compartment. In contrast, genes encoding nuclear proteins, such as Polr2c, Sfpq and Nono, were significantly enriched in the somatic compartment (Fig 1C). GO‐term analysis indicates the overrepresentation of specific cellular components in these compartments: ribosomal subunits, postsynaptic density and mitochondrial respiratory chain complex in the process compartment: extracellular matrix, extracellular space and secretory granule lumen in the somatic compartment (Fig 1D; Appendix Fig S1). Importantly, we observed a very high correlation between our RNA‐seq dataset and a recently published meta‐analysis (von Kugelgen & Chekulaeva, 2020) of neurite‐enriched mRNAs derived from 11 independent high coverage datasets (Fig EV2A–E). Together, these results are consistent with previous large‐scale studies (Poon *et al*, 2006; Cajigas *et al*, 2012; Zappulo *et al*, 2017; Kuzniewska *et al*, 2020) and suggest that our approach is able to faithfully capture differences in mRNA distribution between neuronal compartments.

We next investigated whether mRNA compartmentalization translated into corresponding changes in the neuronal proteome. Towards this aim, we prepared protein extracts from the somatic and process compartment of primary rat hippocampal neurons and subjected them to label‐free proteomics (see Materials and Methods). In four independent preparations, we identified unique peptides corresponding to a total of 4,250 different proteins in the somatic/process compartment, demonstrating a high sensitivity of our approach despite the low amount of starting material. Principal component analysis (PCA) of the replicates indicates a high reproducibility of both the RNA‐seq (Appendix Fig S2A) and proteomics (Appendix Fig S2B) workflow. Similar to transcriptomics, a large number of neuronal proteins displayed preferential localization to either the somatic or process compartment (Fig 1E). For most differentially localized mRNAs, the respective proteins showed a corresponding compartment‐specific expression (e.g. Syn1, Shank2/3, Dlg4, Homer1, Polr2c) (Fig 1F). This suggests that local mRNA translation in processes contributes to the compartment‐specific expression of those genes. However, for some genes (e.g. the presynaptic Sv2a and Syt1), mRNA and protein enrichment did not match, suggesting that protein transport plays a major role in the subcellular localization of these genes.

**Figure 2:**
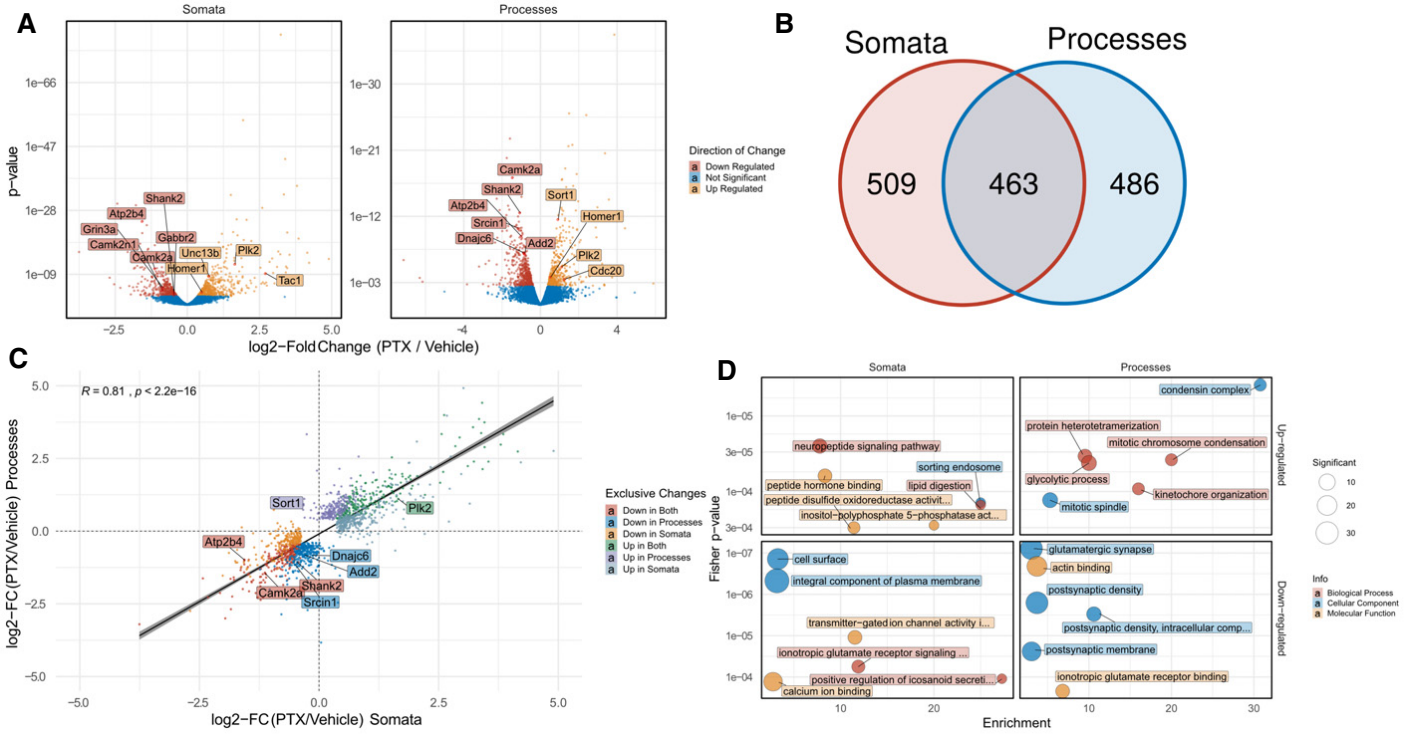
Compartment‐specific regulation of neuronal transcripts by PTX Volcano plots representing transcript down‐ (red) or up‐regulation (yellow) after 48 h PTX in the somatic and process compartment. Representative transcripts are highlighted and labelled. Linear model was used to fit transcriptome data and subsequently contrasted using likelihood ratio testing for PTX treatment effects across compartments.Venn‐Diagram of differentially expressed transcripts between somatic and process compartmentSpearman rank correlation between log_2_‐fold changes of differentially expressed genes significant in either the somatic or process compartment colour‐coded for exclusive changes in the respective compartments. Representative genes are highlighted and labelled. The statistical significance of Spearman correlations was calculated in the standard way, using Spearman's rho statistic. Regression lines indicate fitted linear models, with the light grey shaded areas depicting the 95% confidence interval.Top 6 Gene Ontology Pathways enriched in differentially expressed genes in either the somatic or process compartment colour‐coded for Cellular Component (blue), Molecular Function (yellow) or Biological Process (red).

**Figure EV3:**
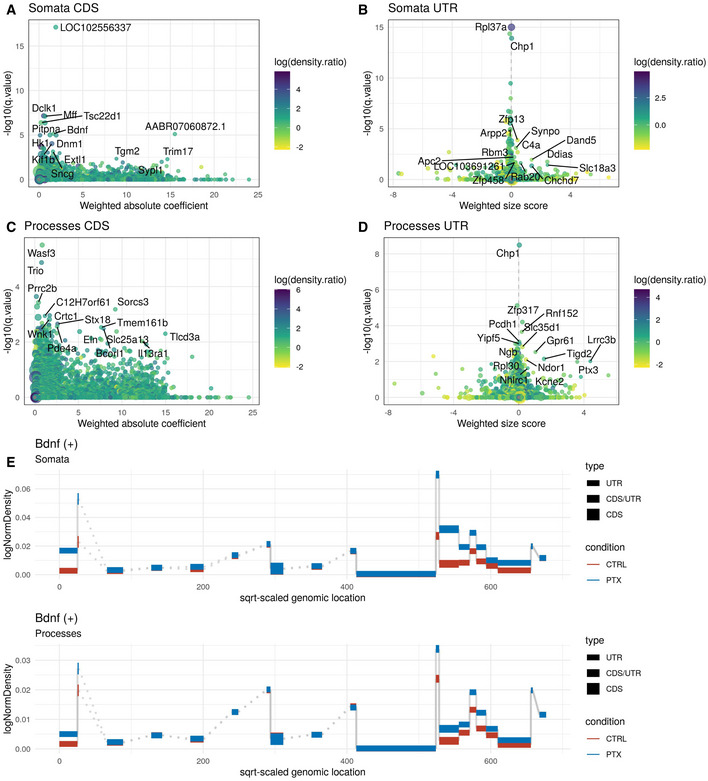
Differential exon‐usage (DEU) analysis upon PTX treatment in the somata and processes compartment A–DGene‐Level statistic plots of the DEU analysis in each compartment, separately for the coding sequence (CDS, left) and 3′UTR (right), with the top 15 candidates labelled. Gene‐level estimates of effect sizes (bin‐level coefficients weighted by significance) are displayed on the *x*‐axis in CDS plots (A + C), whereas the weighted size score in 3′UTR plots (B + D) includes a further length weighing (see Gerber *et al* (2021) for further details).EBin‐level plot displaying different exon‐usage upon PTX treatment in both compartments for the example gene Bdnf.

Having validated our cell culture model, we went on to investigate dynamic compartment‐specific changes in the transcriptome/proteome upon synaptic downscaling in response to 48 h PTX application. Using Ribo(−)‐RNAseq, we identified hundreds of RNAs that were differentially expressed (*q* < 0.05) between PTX‐ and mock‐treated conditions in the somatic (*n* = 972; 431 down/541 up) and process (*n* = 949; 525 down/424 up) compartment (Fig 2A). Of those, about 50% (463) were common to both compartments, demonstrating that a large fraction of PTX‐responsive RNAs displays compartment‐specific regulation (Fig 2B). To better visualize compartment‐specific effects of PTX, we plotted logFC in somata vs logFC in processes and colour‐coded gene groups according to their regulation (Fig 2C). Examples of candidate mRNAs within each of these gene groups are marked in Fig 2C, e.g. process up (Sort1), process down (Srcin1, Add2, Dnajc6) and genes commonly up‐ (Plk2) or downregulated (Camk2a, Atp2b4, Shank2) in either compartment. In addition to protein‐coding mRNAs, PTX also altered the expression of many non‐coding RNAs in a compartment‐specific manner, e.g. long non‐coding RNAs (lncRNAs) and primary microRNAs (miRNAs) (Appendix Fig S3). Furthermore, we detected numerous examples of genes which display compartment‐specific alterations in exon/UTR usage upon PTX treatment (Fig EV3A–D), e.g. BDNF (Fig EV3E). Exon‐Intron split analysis (EISA) (Appendix Fig S4) and differential expression analysis based on RNA half‐lives (Appendix Fig S5) further indicates that PTX‐dependent regulation of many genes cannot be solely explained by transcriptional effects. To obtain insight into the biological function of compartment‐specific regulation by PTX, we performed GO term analysis (Fig 2D; Appendix Fig S6). Genes specifically upregulated in the process compartment were often associated with the cell cycle (e.g. Cdc20) or metabolism, whereas “somata up genes” were enriched in neuropeptide signalling (e.g. Tac1) and intracellular trafficking (e.g. Sort1). Consistent with PTX‐mediated downscaling of excitatory synapses, PTX‐downregulated genes in both compartments were highly enriched for regulators of excitatory synaptic transmission. However, whereas the majority of “somata down genes” encode for regulators of synaptic excitation, e.g. ionotropic glutamate receptors (subunits of the AMPA, NMDA and Kainate type) and transmembrane transporters (e.g. several members of the Slc family), “process down genes” mostly encode for structural components of the postsynaptic density, e.g. scaffolding (e.g. Shank1,3, Dlg3, Srcin1, Syngap1, Dlgap1, Homer2/3) and cytoskeletal proteins (e.g. Add2, Dnm1, Map1a/b/s). Taken together, our results demonstrate that compartment‐specific regulation of the neuronal transcriptome during synaptic scaling is more pervasive than previously anticipated.

**Figure 3:**
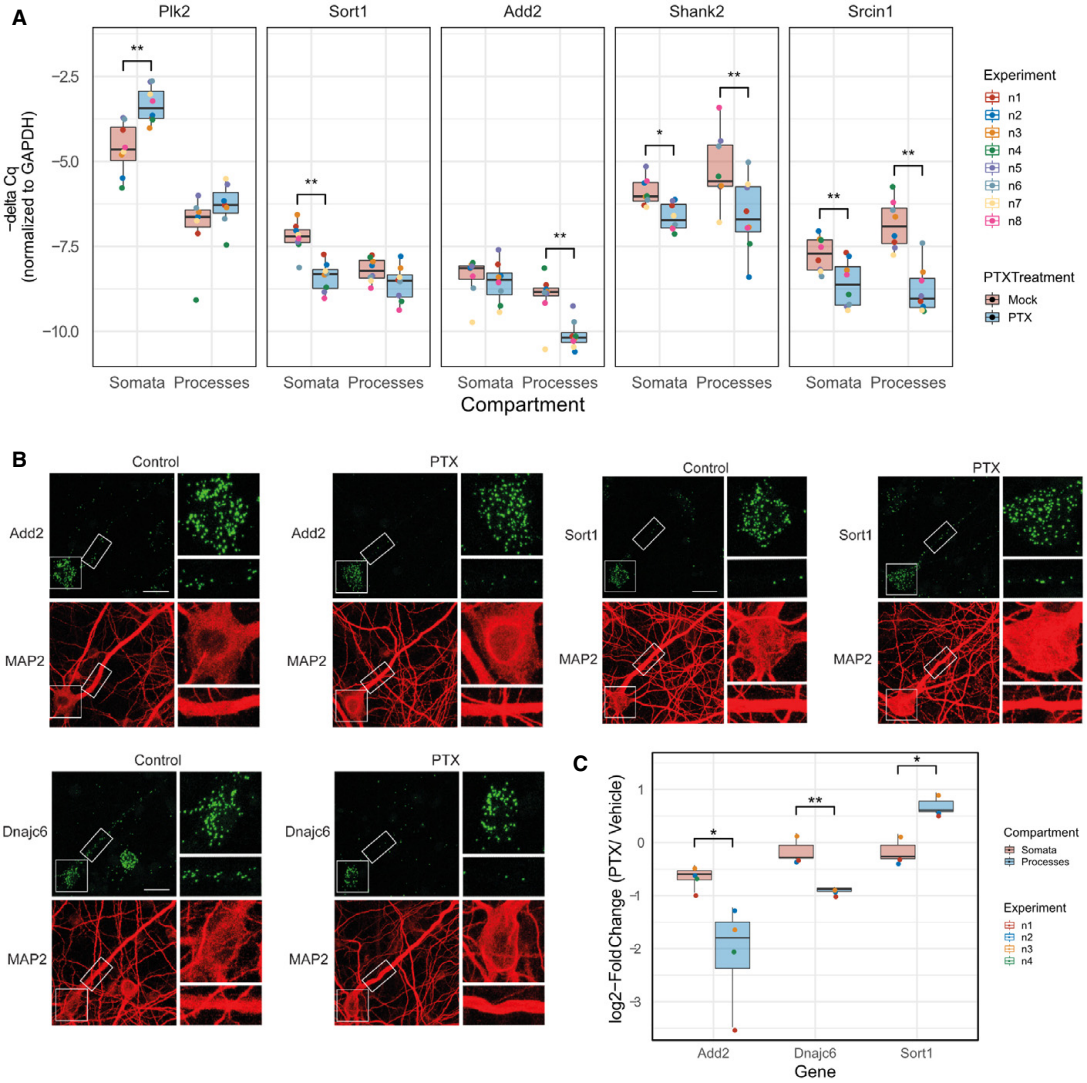
Validation of compartment‐specific regulations Real‐time quantitative PCR (RT–qPCR) of transcripts changing differentially between compartments using the compartmentalized cultures after 48 h PTX treatment. *n* = 8 independent biological replicates (except for Plk2 (*n* = 7), where one measurement couldn'*t* be performed due to insufficient cDNA); PTX effect was assessed by three‐way ANOVA followed by Tukey's *post hoc* multiple comparison test; *P < 0.05; **P < 0.01.Representative images of single‐molecule FISH (smFISH) in either control or 48 h PTX‐treated rat hippocampal neurons (DIV20) using probes specific for Add2, Dnajc6 and Sort1 (green). MAP2 immunostaining (red) was used to visualize neuronal somata and dendrites. Inserts at higher magnification illustrate PTX‐dependent changes in dendritic RNA puncta. Scale bar = 10 μm.Quantification of (B). (*n* = 3–4 independent biological replicates with 8–10 cells averaged per condition and replicate; two‐sample Student's *t*‐test; *P < 0.05; **P < 0.01).Data information: Boxplots: central line: median; box: 25^th^ to 75^th^ percentile; whiskers: until last data point within 1.5× interquartile range (IQR).

We went on to validate selected high‐ranking candidates that display robust compartment‐specific regulation by PTX, particularly focusing on genes that are preferentially regulated in the process compartment. Using qPCR, we validated PTX‐dependent differential regulation of several genes, such as Sort1, Add2, Shank2 and Srcin1 (Fig 3A, Appendix Fig S7). Furthermore, we detected up‐regulation of Plk2 in either compartment, consistent with a previous report (Seeburg *et al*, 2008). Compartment‐specific regulation by PTX was further confirmed for Add2, Sort1 and Dnajc6 at the single neuronal level using single‐molecule fluorescent *in situ* hybridization (smFISH) (Fig 3B and C; Appendix Fig S7). smFISH further allowed us to distinguish between dendritic and axonal processes. Whereas PTX selectively reduced dendritic Add2 and Dnajc6 mRNA puncta, Sort1 mRNA puncta were significantly increased in dendrites. For all three genes, levels of somatic mRNA puncta were unaffected by PTX treatment.

**Figure 4:**
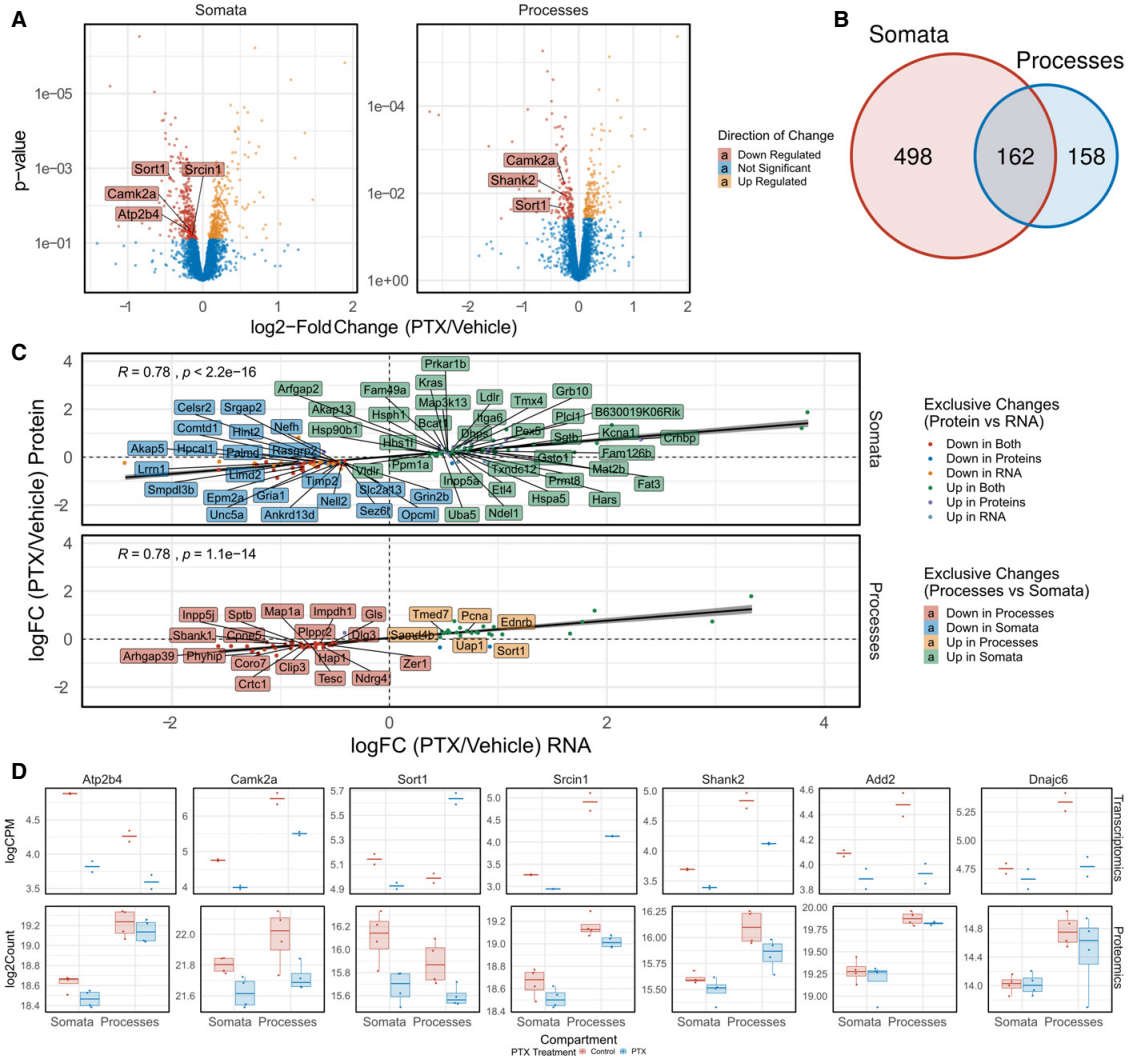
Compartment‐specific regulation of neuronal proteins by PTX Volcano plots representing proteins down‐ (red) or up‐regulation (yellow) after 48 h PTX in the somatic and process compartment. FDR < 0.5. Representative proteins are highlighted and labelled. Linear model was used to fit proteome data and subsequently contrasted using empirical Bayes statistics for PTX treatment effects across compartments.Venn‐Diagram of differentially expressed proteins between somatic and process compartment.Spearman rank correlation between log_2_‐fold changes of differentially expressed genes changing at the transcript and protein level. The statistical significance of Spearman correlations was calculated in the standard way, using Spearman's rho statistic. Regression lines indicate fitted linear models, with the light grey shaded areas depicting the 95% confidence interval.Representative plots of genes changing after PTX at the transcript and protein level. (Upper row: Scatterplots of transcript level; *n* = 2; crossbar represents mean. Lower row: Boxplot of protein levels; *n* = 4). Boxplots: central line: median; box: 25^th^ to 75^th^ percentile; whiskers: until last data point within 1.5× interquartile range (IQR).

**Figure EV4:**
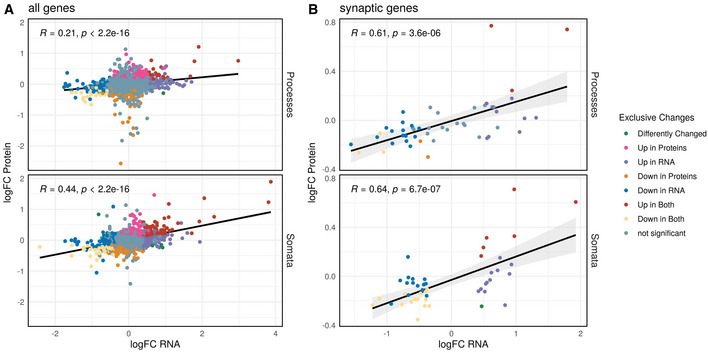
Correlation of changes in RNA and Protein abundance upon PTX treatment Pearson's correlation of RNA‐ and Protein logFC upon PTX treatment in both compartments. The statistical significance of Pearson correlations was calculated in the standard fashion, i.e. by testing against a null hypothesis of *r* = 0 using a *t*‐distribution with *N*‐2 degrees of freedom. Regression lines indicate fitted linear models, with the light grey shaded areas depicting the 95% confidence interval.
ACorrelation with all genes detected in both assays.BSubset of only synaptic genes (see Fig 6). Coloured are the changes in both compartments (RNA significance with FDR < 0.05, Protein significance with FDR < 0.5).

**Figure EV5:**
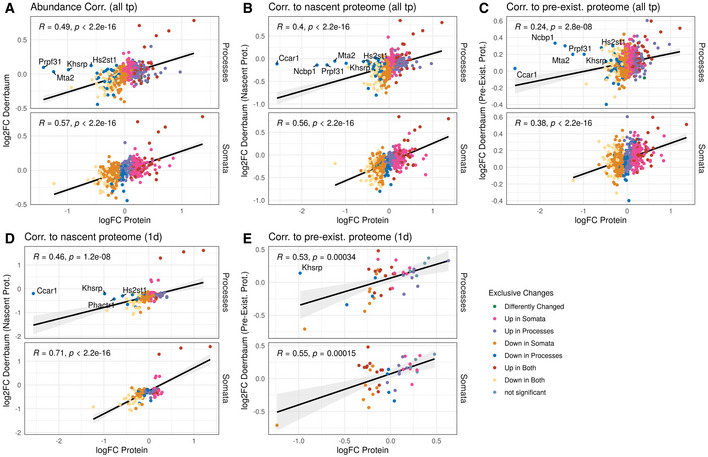
Comparison of PTX effects on protein changes to the dynamics published in Dorrbaum *et al* (2020) Pearson's correlation of significantly changing proteins (FDR < 0.5) upon PTX treatment in both compartments to protein dynamics determined by a whole cell SILAC experiment subsequent bicuculline treatment (Dorrbaum *et al*, 2020). The statistical significance of Pearson correlations was calculated in the standard fashion, i.e. by testing against a null hypothesis of *r* = 0 using a *t*‐distribution with *N*‐2 degrees of freedom. Regression lines indicate fitted linear models, with the light grey shaded areas depicting the 95% confidence interval. Proteins significantly downregulated in the processes compartment with a logFC of less than −0.5 are highlighted in the processes panel.
Correlation with changes in protein abundance as determined by Dörrbaum and colleagues upon bicuculline treatment over the time period of 7 days (three time points of protein collection).Correlation with changes of the nascent (newly synthesized) proteome over the 7 days' time course.Correlation with changes of the pre‐existing proteome over the 7 days' time course.Correlation with changes of the nascent proteome seen by Dorrbaum *et al* (2020) after the time point of 1 day.Correlation with changes of the pre‐existing proteome at the 1‐day time point.

We next wondered whether the PTX‐induced compartment‐specific changes in transcript levels translated into corresponding changes in protein. Although dynamic time‐ and polarity‐dependent changes in the neuronal proteome have been previously demonstrated in synaptic scaling using labelled proteomics (Schanzenbacher *et al*, 2016; Schanzenbacher *et al*, 2018; Dorrbaum *et al*, 2020), these studies did not address potential compartment‐specific effects. Therefore, we extracted proteins from PTX‐treated compartmentalized hippocampal neuron cultures and quantified proteome‐wide changes using label‐free proteomics. In total, we detected peptides corresponding to 4,250 different proteins with our approach. Differential expression analysis revealed that 660 proteins were significantly changing in somata and 320 proteins in processes, respectively (FDR < 0.5, Fig 4A; Appendix Fig S8). Much like our observations taken from RNA‐seq, we detected substantial compartment‐specific regulation of protein levels during synaptic downscaling, with only 162 proteins commonly regulated in both the somatic and process compartment (Fig 4B). Comparable with the RNA‐data, GO‐term analysis of differentially expressed proteins indicated a strong enrichment of synaptic proteins among PTX‐downregulated proteins (Appendix Fig S9). Importantly, although many of the top‐ranking PTX‐regulated mRNAs were not detectable by proteomics, PTX‐dependent changes in RNA and protein levels in both compartments strongly correlated, in particularly for genes encoding synaptic proteins (Fig 4C and EV4A and B; Appendix Fig S10A and B), e.g. preferential downregulation of Shank1, Dlg3, Crtc1 and Map1a in the process‐ and Grin2b, Slc2a13 in the somatic compartment, respectively. A correlation of PTX‐dependent changes in RNA and protein levels is also observed for most of the candidates validated by qPCR (Fig 4D). However, for a few genes, PTX‐dependent changes in protein and RNA levels did not correlate. For example, Sort1 is significantly upregulated at the RNA level (Fig 3C), but decreased at the protein level in the process compartment (Fig 4D). This result suggests that additional regulatory mechanisms at the level of mRNA translation and/or protein turnover contribute to PTX‐dependent process expression of those genes. To obtain further insight about compartment‐specific effects of PTX on protein expression, we compared our dataset to a previous publication which investigated protein synthesis and degradation in response to the GABA‐A‐R antagonist bicuculline independent of cellular compartments (Dorrbaum *et al*, 2020). In general, we observed a significant positive correlation between protein logFC, suggesting that PTX and Bic induce similar proteomic changes (Fig EV5A). Interestingly, when focusing only on nascent proteins (those which are arguably more strongly correlated with RNA abundance), the correlation was much stronger for proteins which significantly change in the process compared to the somatic compartment (Fig EV5B–E). Accordingly, many of the proteins we found strongly downregulated in the process compartment (e.g Khsrp, Ccar) were only modestly altered in the study by Dorrbaum *et al*. Together, this result underscores the utility of our compartment‐specific approach for the detection of local changes in protein abundance at high spatiotemporal resolution.

**Figure 5:**
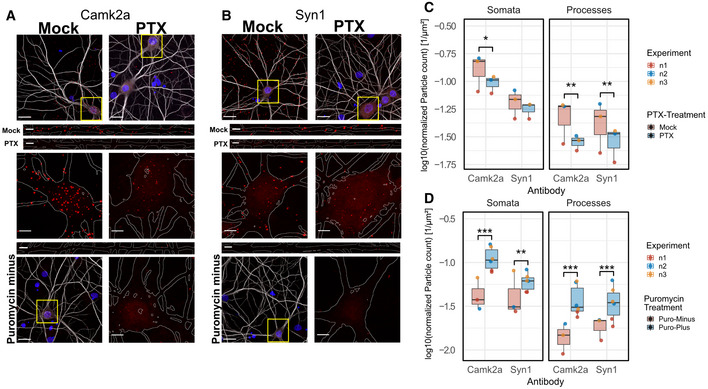
PTX‐mediated regulation of local translation Representative pictures of nascent Camk2a‐peptides in mock‐ and PTX‐treated neurons. Upper Panels: Representative overview images with merged channels (grey: MAP2‐staining, red: Duolink‐PLA signal, blue: Hoechst nuclear staining). Middle Panels: Magnifications of boxed areas marked in (A). MAP2 signal was used to outline somata and dendritic processes (white lines). Lower Panels: Negative control of puromycin‐untreated cells with representative Duolink signal in dendrite and soma, respectively. (Scale bars: in overview images 20 μm, in inserts of higher magnification 5 μm).Same as (A) with nascent Synapsin1‐peptides.Quantification of Duolink‐particle density of (A) and (B) assessing PTX treatment effect. *n* = 3 independent experiments with 10 images averaged per condition and experiment; Three‐way ANOVA followed by Tukey's *post hoc* multiple comparison test; *P < 0.05; **P < 0.01).Quantification of Duolink‐particle density of (A) and (B) assessing Puromycin‐treatment effect. *n* = 3 independent experiments for Puromycin‐Minus and *n* = 6 data points for Puromycin‐Plus conditions (PTX and mock conditions combined together across *n* = 3 independent experiments, taken into account in ANOVA model) with 10 images averaged per condition and experiment; three‐way ANOVA followed by Tukey's *post hoc* multiple comparison test; **P < 0.01, ***P < 0.001).Data information: Boxplots: central line: median; box: 25^th^ to 75^th^ percentile; whiskers: until last data point within 1.5× interquartile range (IQR).

In an effort to unequivocally address local regulation of nascent protein synthesis, we performed the previously published puromycin proximity ligation assay (PLA) (tom Dieck *et al*, 2015) for selected candidates (Fig 5). For this analysis, we chose Camk2a (downregulated in both somatic and process compartment) and Syn1 (downregulated selectively in the process compartment) based on our results from RNA‐seq and proteomics. Whereas puncta labelling nascent Camk2a protein were significantly reduced in both the somata and dendrites of PTX‐ compared to mock‐treated neurons (Fig 5A and C), Syn1 puncta density was only significantly reduced in dendrites and remained unchanged in somata (Fig 5B and C). In the absence of puromycin treatment (lower panels in Figs 5A, B and D), signal intensity was strongly reduced, demonstrating the specificity of the assay. Together, these data confirm the compartment‐specific regulation of nascent protein synthesis by PTX for selected candidates.

**Figure 6:**
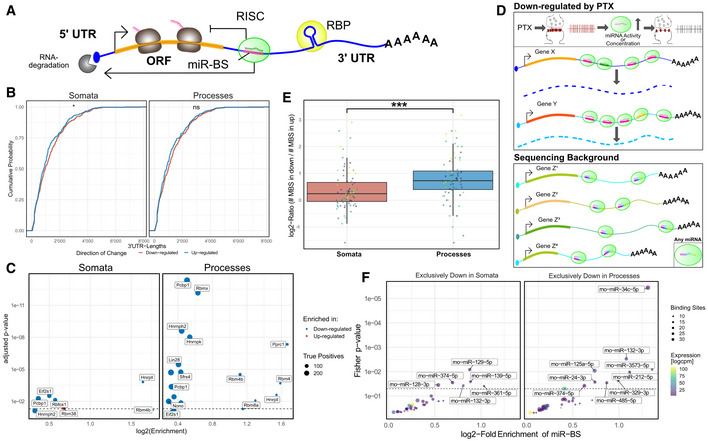
3′ UTR‐dependent mechanisms of regulation by RBPs and miRNAs Schematic of cis‐acting RNA‐binding proteins (RBPs) and microRNAs (miRNAs) and their effects on mRNA stability.Cumulative distribution of 3′‐UTR lengths of down‐ and upregulated genes in either compartment. Two‐sample Kolmogorov–Smirnov test; ns > 0.05, *P‐value < 0.05.Enrichment analysis for RBP motifs in down‐ and upregulated genes in either compartment. Fisher's exact test was used to perform enrichment analysis; FDR‐corrected.Schematic of miRNA‐binding site (MBS) enrichment. PTX‐mediated synaptic downscaling might lead to a local increase in miRNA abundance or activity. These effects can be potentially captured by testing for an enrichment of MBS in 3′UTRs of downregulated genes in comparison with the sequencing background (expressed genes).Log_2_‐Ratios of the number of miRNA‐binding sites found in significantly up‐ vs downregulated genes in the somata (red) or processes (blue). Elevated ratio in processes indicates that miRNA‐binding sites are more frequent in transcripts downregulated by PTX in processes. Non‐parametric Mann–Whitney *U*‐test was used to test difference (mean somata: 0.37 vs mean processes: 0.783); ***P‐value < 0.0001; *n* = 80 miRNAs, represented as coloured data points. Boxplots: central line: median; box: 25^th^ to 75^th^ percentile; whiskers: until last data point within 1.5× interquartile range (IQR).Enrichment of MBS in genes that are exclusively downregulated in somata or processes compared to the sequencing background. The significant miRNA families (*P*‐value < 0.05 and log_2_‐fold enrichment > 0.5) are labelled by a representative miRNA. See Methods for detailed statistical analysis.

Next, we sought to characterize the molecular mechanisms which underlie the compartment‐specific regulation by PTX, focusing on dynamic changes in mRNA levels. Untranslated regions (UTRs) of mRNAs are considered as important determinants of both mRNA localization and post‐transcriptional regulation. 3′UTRs in particular contain sequence elements for trans‐acting post‐transcriptional regulators, such as RNA‐binding proteins (RBPs) and miRNAs (Fig 6A). We first determined the average 3′UTR length in genes regulated in a compartment‐specific manner (Fig 6B). 3′UTRs of PTX‐downregulated genes were slightly longer in comparison with upregulated genes in both the somatic and process compartment, although the difference was only statistically significant in the former compartment. Thus, 3′UTR length differences alone unlikely explain the high degree of compartment‐specific gene regulation during synaptic scaling.

Subsequently, we investigated the compartment‐specific enrichment of specific sequence motifs located within 3′UTRs. We first interrogated overrepresentation of RBP binding motifs determined in a previous large‐scale *in vitro* study (Ray *et al*, 2013) in the 3′UTRs of PTX‐regulated mRNAs (Fig 6C). Whereas only five motifs were significantly (adjusted *P*‐value < 0.05) overrepresented in PTX‐regulated somatic mRNAs, 16 motifs fulfilled these criteria in PTX‐regulated process‐enriched mRNAs (Fig 6C). Interestingly, only one motif in total could be recovered from PTX‐upregulated genes, suggesting that RBPs acting by 3′UTR‐dependent mechanisms are mostly involved in PTX‐mediated gene repression. Among the motifs, most highly enriched in PTX‐downregulated genes in somata is the Rbfox1 consensus binding site (GCAUG). This fits well with our previous results showing an important role of Rbfox1 in synaptic scaling at the level of the entire neuron (Rajman *et al*, 2017). Concerning process‐enriched RBP motifs, many of them are recognized by known regulators of mRNA stability and translation (e.g. hnRNP‐K (Folci *et al*, 2014; Bottini *et al*, 2017; Leal *et al*, 2017), Nono (Bottini *et al*, 2017) and Lin‐28 (Amen *et al*, 2017)). This suggests an important contribution of RBPs to compartment‐specific gene regulation during synaptic scaling.

**Figure 7:**
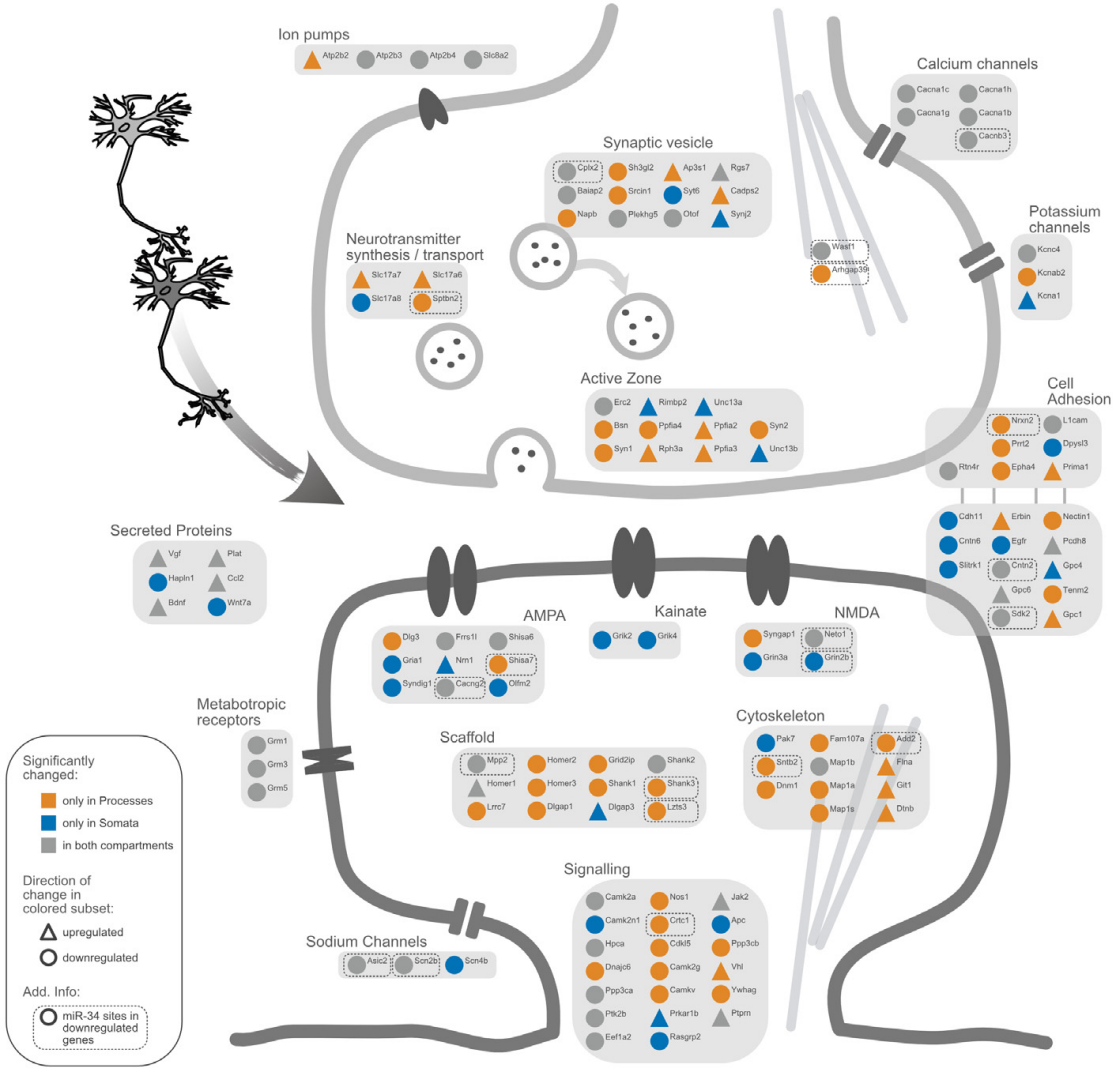
PTX‐mediated changes in synaptic genes Schematic representation of significantly changing genes (compartment RNA‐sequencing) associated with excitatory synapses. Genes differentially expressed in only one compartment are highlighted with either yellow (processes compartment) or blue (somata compartment). The direction of change is illustrated as symbol shape (circle = sign. downregulated genes, triangle = sign. upregulated genes). miR‐34 family binding sites in sign. Downregulated genes are marked with a dashed line.

miRNAs represent an additional class of gene‐specific regulatory molecules that negatively affect mRNA expression by recruiting a silencing protein complex to specific sites (miRNA‐binding sites, MBS) in 3′UTRs (Bartel, 2009). To estimate miRNA contribution to PTX‐dependent regulation, we performed an MBS enrichment analysis. Briefly, the number of MBS within a gene set of interest (i.e. PTX‐downregulated genes in the process compartment) were compared to those present in the sequencing background (Fig 6D; see Materials and Methods for further details). We calculated enrichment scores for MBS of neuronally expressed miRNAs (*n* = 221) in the 3′UTR of genes that are downregulated by PTX in a compartment‐specific manner. In both compartments, MBS were on average more abundant in PTX down‐ compared to upregulated genes (log_2_[PTX/control] > 0; Fig 6E), as expected. However, the enrichment value (log_2_‐fold ratio) obtained from processes was significantly higher compared to somata (Fig 5E; *P* < 0.001). Taken together, these results suggest that miRNA‐dependent mRNA repression plays a particularly important role in the process compartment during synaptic downscaling. At the level of individual miRNA families, specific sets of MBS were significantly enriched over background in PTX‐downregulated genes in either the somatic or process compartment (Fig 6F). Our approach independently identified miR‐129‐5p, which we previously showed to be required for synaptic downscaling (Rajman *et al*, 2017), as the miRNA whose binding motifs were most strongly enriched in PTX‐downregulated genes in the somatic compartment. This finding indicates that our approach is able to identify functionally important miRNAs solely on the basis of differential expression analysis. On the other hand, our enrichment analysis identified an overrepresentation of several miRNA‐binding motifs in mRNAs that are specifically downregulated by PTX in the process compartment, such as the activity‐regulated miR‐132/212 (Wayman *et al*, 2008), miR‐24‐3p, miR‐125a‐5p, and the miR‐379‐410 cluster members miR‐329‐3p and miR‐485‐5p (Lackinger *et al*, 2019). One miRNA that appears to be specifically active in dendrites during PTX‐induced downscaling is miR‐34c‐5p, a member of the miR‐34/449 family (Fig 5F, Appendix Fig S11). A total of 53 PTX‐downregulated genes in processes contain miR‐34/449 binding motifs in their 3′UTRs, including important synaptic regulatory proteins such as Add2, Shank3, Cntn2, Cacng2, Scn2b, Mpp2 and Crtc1 (Torc1) (Fig 7).

Taken together, compartment‐specific regulation of neuronal transcripts during synaptic downscaling is associated with specific 3′UTR features, including unique miRNA and RBP motifs. Post‐transcriptional regulation by miRNAs and RBPs appears to be particularly widespread during PTX‐mediated downregulation of synaptic genes in the process compartment.

## Discussion

In this study, we provided the first comprehensive analysis of compartmentalized gene expression during synaptic plasticity, more specifically in response to homeostatic synaptic downscaling induced by prolonged treatment of primary rat hippocampal neurons with the GABA‐A‐R blocker PTX. A major outcome of our study was the observation that PTX treatment induces compartment‐specific changes in the neuronal transcriptome and proteome. The existence of local and global mechanisms in homeostatic plasticity has been known for some time (Turrigiano, 2012), but a clear picture of how homeostatic feedback is structured at the molecular level has not yet emerged. Our study lends further support to the hypothesis that local synaptic and neuron‐wide mechanisms co‐exist in neurons and act in concert to counteract excessive excitation which would inevitably occur if feedforward Hebbian plasticity operated unconstrained (Rabinowitch & Segev, 2008).

What distinguishes the somatic and process compartment during synaptic scaling? Some clues might come from our GO‐term analysis, which showed a strong overrepresentation of genes involved in the regulation of neuronal excitability (e.g. ion channels, transporters, receptors) in the cell body, whereas the majority of regulation of structural synaptic genes (e.g. postsynaptic scaffolds, cytoskeletal proteins) occurred in the process compartment (Fig 7). Consistent with this observation, many of the process‐regulated mRNAs are known dendritic mRNAs, e.g. Shank1/3 and Homer2 and Syngap1, and have recently been shown to be preferentially translated in the synaptic neuropil *in vivo* (preprint: Glock *et al*, 2020). On the other hand, “neuronal excitability genes” encode mostly transmembrane proteins, for which the somatic biosynthesis machinery might represent the major source, although dendritic translation has been demonstrated for some of these genes. Thus, mRNA sorting under basal conditions might already pre‐determine compartment‐specific responsiveness during scaling or other plasticity‐inducing cues.

What are the molecular mechanisms underlying the compartment‐specific regulation during synaptic scaling? In principle, compartment‐specific mRNA changes could be a result of altered mRNA transcription, transport, stability and translation, or combinations thereof. Our experimental setup does not allow to disentangle these intimately coupled processes. However, several lines of evidence suggest that increased mRNA degradation could play a major role, at least in the context of downregulation of synaptic gene expression in processes. First, mRNA and protein levels of differentially expressed genes strongly correlated in both compartments (Figs 4C and EV4), which argues in favour of a mechanism which involves alterations in mRNA abundance. Second, PTX did not uniformly reduce mRNA levels of synaptic genes across departments and also did not affect short‐lived mRNAs more strongly (Appendix Fig S5), as would have been expected if these changes were purely a result of transcriptional inhibition. Third, we did not obtain evidence for a massive PTX‐dependent relocation of candidate mRNAs between compartments from RNA‐seq and smFISH (Fig 3B), arguing against a major contribution of PTX‐regulated RNA transport. In this regard, the inhibitory effects of PTX observed in the local mRNA translation assay (Puro‐PLA) might be primarily a consequence of reduced levels of the corresponding mRNAs, although additional effects on mRNA translation efficiency cannot be ruled out. In the future, more direct approaches to investigate local mRNA degradation (e.g. TREAT (Horvathova *et al*, 2017), 4‐thiouridine metabolic labelling (Herzog *et al*, 2017)) will have to be performed.

The striking overrepresentation of motifs for miRNAs and RBPs in 3′UTRs of PTX‐regulated genes which are selectively downregulated in processes (Fig 6C and F) further suggests that gene‐specific post‐transcriptional mechanisms might play a particularly important role in the local control of synaptic gene expression. Regarding miRNAs, many of the PTX‐downregulated synaptic genes (e.g. Add2, Shank3, Cntn2, Cacng2, Scn2b, Mpp2) contain binding sites for the miR‐34/449 family. A synaptic function of miR‐34 was very recently reported in *Drosophila*, where it controls pre‐ and postsynaptic function at the neuromuscular junction (NMJ) (McNeill *et al*, 2020). miR‐34 has also been implicated in stress‐associated disorders, e.g. anxiety and epilepsy (Sano *et al*, 2012), providing potential links between miR‐34, synaptic scaling and neurological disease which should be explored in the future. Additional candidate miRNAs for follow‐up studies include miR‐24, miR‐132 and miR‐125, which are high‐ranking synaptic miRNAs according to a recent study (Epple *et al*, 2021). Concerning RBPs, motifs for several members of the hnRNP family, Lin‐28 and Nono were specifically enriched in “process down genes”, consistent with their reported presence in dendritic RNA granules (Kanai *et al*, 2004). Among them, hnRNP‐K might be a strong candidate for follow‐up experiments. Since hnRNP‐K has mostly stabilizing functions on mRNA (Nagano *et al*, 2006; Shanmugam *et al*, 2008; Proepper *et al*, 2011), one possible scenario is that it counteracts miRNA activity under basal conditions and is subsequently inactivated upon scaling. In a broader context, specific combinations of motifs for trans‐acting regulatory factors (“regulons”) might underlie the spatiotemporal control of neuronal gene expression in response to extracellular cues.

What could be the biological relevance of compartmentalized gene expression during homeostatic scaling? One can speculate that it might allow neurons to maintain excitability while taking into account individual plasticity needs of synapses or dendritic domains (Rabinowitch & Segev, 2006). Intriguingly, a very recent study described the local allocation of proteins in so‐called “synaptic neighborhoods”, about 10 μm long dendritic domains, in a related form of homeostatic plasticity, synaptic upscaling (preprint: Chao Sun, 2020). In agreement with our results, many of these allocated proteins might arise from local dendritic mRNA translation. Thus, local or quasi‐local mechanisms operating at the level of synapses, neighbourhoods or dendritic domains might represent a common feature of homeostatic plasticity in excitatory neurons.

In conclusion, system‐wide approaches like the one presented here might help to disentangle the spatiotemporal logic of the complex regulatory networks underlying synaptic plasticity and homeostasis and their aberrations in diseases such as epilepsy and autism.

## Materials and Methods

### Cell culture, transfection and stimulation

Primary cultures of Sprague Dawley rats (Charles River Laboratories, Sulzfeld, Germany) embryonic hippocampal neurons were prepared as described previously (Schratt *et al*, 2006) and plated onto porous membrane cell culture inserts as described previously (Bicker *et al*, 2013). For stimulation, 18DIV neurons were treated either with Picrotoxin (PTX; 100 μM final concentration, Sigma) or vehicle (ethanol absolute) for 48 h and lysed at 20DIV. All animal experiments were carried out under institutional guidelines (ZH196/17 Kanton Zürich Gesundheitsdirektion Veterinäramt).

### Electrophysiology

Whole cell patch clamp recordings were performed on an upright microscope (Olympus BX51WI) at room temperature. Data were collected with an Axon MultiClamp 700B amplifier and an Digidata 1550B digitizer and analysed with pClamp 11 software (all from Molecular Devices). Recording pipettes were pulled from borosilicate capillary glass (Harvard Apparatus; GC150F‐10) with a DMZ‐Universal‐Electrode‐Puller (Zeitz) and had resistances between 3 and 4 MΩ.

Miniature EPSC (mEPSC) were recorded from primary cultured hippocampal neurons after 48 h of picrotoxin treatment at DIV 21–23. The extracellular solution was composed of (in mM) 140 NaCl, 2.5 KCl, 10 HEPES, 2 CaCl_2_, 1 MgCl_2_, 10 glucose (adjusted to pH 7.3 with NaOH), the intracellular solution of (in mM) 125 K‐Gluconate, 20 KCL, 0.5 EGTA, 10 HEPES, 4 Mg‐ATP, 0.3 GTP and 10 Na_2_‐Phosphocreatine (adjusted to pH 7.3 with KOH). 1 μM TTX and 1 μM Gabazine were added to the extracellular solution to block action‐potential driven glutamate release and GABAergic synaptic transmission, respectively. Cells were held at −70 mV. The sampling frequency was 5 kHz and the filter frequency 2 kHz. Series resistance was monitored, and recordings were discarded if the series resistance changed significantly (≥ 10%) or exceeded 20 MΩ.

### Single molecule fluorescence *in situ* hybridization

Dissociated hippocampal neurons were fixed at 20DIV using 4% paraformaldehyde/4% sucrose/PBS for 30 min at room temperature. FISH was performed using the QuantiGene (QG) ViewRNA kit (Affymetrix) according to the manufacturer's protocol using probes for Add2, Dnajc6 and Sort1. The protease treatment step was omitted in to maintain dendritic integrity. After completion of the FISH protocol, cells were processed for immunostaining, using an anti‐MAP2 antibody (1:1,000, Sigma Cat. Nr.: M9942, RRID: AB_477256) in GDB buffer (0.02% gelatin–0.5% Triton X‐100–PBS). Images were acquired on a Leica SP5 laser‐scanning confocal microscope. For z‐stack images of whole cells, 12 consecutive optical sections were taken at a 0.4 μm interval with a resolution of 1,024 × 1,024 pixels using a 63× objective and a digital zoom factor of 2.5. For images of cell bodies, 15 consecutive optical sections were taken at a 0.4 μm interval with a resolution of 512 × 512 pixels using a 63× objective and a digital zoom factor of 5. For all pictures, the pinhole was set to 1 AU. Laser settings were kept constant between experimental conditions. Maximum intensity projections of the z‐stacks were used for signal quantification, which was conducted in a blinded manner. The density of RNA particles was analysed by CellProfiler (Carpenter *et al*, 2006), using the MAP2 immunostaining as mask to define cell bodies and dendrites.

### Puromycin‐proximity ligation assay

Detection of translation foci was performed using an antibody against puromycin after metabolic labelling of ribosomes by puromycin and an antibody against the nascent peptide of interest as previously described (tom Dieck *et al*, 2015). The Duolink in situ orange PLA mouse/rabbit kit (Sigma) allows the spatial coincidence detection (< 40 nm) of these two antibodies via a rolling circle amplification step using fluorescently labelled oligonucleotides. For better background estimation, conditions where cells were left untreated of puromycin but processed with all respective antibodies, were included. In all conditions, cells were incubated with antibodies against puromycin (mouse anti‐puromycin [3RH11], 1:2,500, Kerafast, Cat. Nr.: EQ0001, RRID: AB_2620162), the peptide of interest (either rabbit anti‐Camk2a, 1:500, Thermo Fisher Scientific, Cat. Nr.: PA5‐84083, RRID: AB_2791235, 1:500 or rabbit anti‐Synapsin1, 1:500, Sigma, Cat. Nr. AB1543, RRID: AB_2200400) and the cell‐marker MAP2 (chicken‐anti‐MAP2, 1:1,000, Thermo Fisher Scientific, Cat. Nr.: PA1‐16751, RRID: AB_2138189).

In brief, primary hippocampal neurons were cultured on coverslips as described earlier and treated with 100 nM PTX for 48 h. Before fixation, cells were incubated with puromycin (3 μM final concentration, Invivogen) for 5 min or left untreated for control conditions, briefly rinsed twice with PBS and fixed in 4% sucrose‐PFA for 10 min at room temperature. Cells were then rinsed twice in PBS and incubated in permeabilization buffer (0.05% Triton X, 10% normal goat serum in PBS) for 5min at room temperature. After permeabilization, we proceeded with the Duolink Proximity Assay (Sigma) according to the manufacturer's protocol with slight modifications. The coverslips were placed on parafilm facing up in the wet‐chamber of the RNAscope HybEZ™ II oven (ACD), where all temperature‐sensitive steps were carried out. Cells were first treated with the supplied blocking buffer for 1 h at 37° and then immediately incubated with the primary antibody solution (mouse anti‐puromycin, rabbit anti‐peptide of interest and chicken anti‐MAP2 in supplied antibody diluent) for 1 h at room temperature. All subsequent incubation steps were followed by three washing steps of 5 min each in washing buffer A (supplied by the Duolink kit). After washing, cells were incubated in secondary antibody solutions (anti‐rabbit Plus‐probe, 1:5, anti‐mouse Minus‐probe, 1:5 and anti‐chicken Alexa Plus 647‐secondary antibody, 1:1,000, Thermo Fisher Scientific, Cat. Nr: A32933, RRID: AB_2762845) for 1 h at 37°C followed by a ligation step (30 min at 37°C) and amplification step (100 min at 37°C) according to manufacturer's protocol. The final washing steps consisted of three times 10 min with the supplied washing buffer B, followed by a brief nuclear counterstaining with Hoechst (1:10,000) in PBS and mounting on a glass slide using Aqua‐Poly‐Mount (Polysciences, Cat. Nr: 18606‐20). Imaging was performed within the next week, and the slides stored at 4°C upon further use. Images were acquired in a AiryScan Zeiss LSM 800 in Fast Airyscan optimal high‐resolution acquisition mode (1,920 × 1,920 pixels, Z‐Stacked with an interval of 0.18 μm across the whole cells) using a 63× 1.4 NA oil objective (Plan‐Apochromat 63×/1.4 Oil DIC M27). After inspection of proper Duolink signal and avoiding the edge of the coverslips, 10 images per condition and replicate were acquired using the MAP2‐channel and morphological assessment of the neurons as the only criteria for the selection of cells. Images were post‐processed using the Airyscan‐Processing function on the Zeiss Zen Blue acquisition programme. A custom Python‐script written in the context of the ImageJ‐framework (Schindelin *et al*, 2012) was used for image analysis and is freely available via the ImageJ‐update site (for more information https://github.com/dcolam/Cluster‐Analysis‐Plugin). In brief, to count the Duolink signal within the somatic compartment, a selection mask was created using the MAP2‐channel and the nuclei were detected within to get rid of astrocytic nuclei. The neuronal nuclei were expanded by 3 µm and used as a somatic mask to count the Duolink spots within. To create the selection mask for the process compartment, the somatic mask was inverted and used to detect the MAP2‐channel within the inverted somatic mask. The resulting selection was used as a mask for the process compartment, where the Duolink signal was counted. The count of Duolink signal was normalized by the area of the corresponding selection. The Duolink‐density was then averaged across biological replicate and condition and log_10_‐transformed.

### RNA extraction

RNA was extracted from primary hippocampal neuronal cultures using either peqGOLD Isolation Systems TriFast™ (Peqlab) to prepare sequencing library or mirVana™ total RNA Isolation Kit for quantitative real‐time PCR following the manufacturer's protocol. To remove potential DNA contamination, RNA samples were treated with TURBO™ DNase (Ambion) and RNA was re‐extracted as described before. Samples were stored at −80°C until further use. Prior to RNA extraction, compartmentalized cell culture system was pre‐processed as described (Zheng *et al*, 2001).

### Quantitative real‐time PCR

RNA was reverse‐transcribed with iScript™ reverse transcription supermix (Bio‐Rad) using random hexamers (total RNA) or oligo dT20primers (poly‐RNA) according to manufacturer's instructions. Quantitative real‐time PCR was performed with the Step One Plus Real‐Time PCR System (Applied Biosystems), using iTaq SYBR Green Supermix with ROX (Bio‐Rad) for detection of mRNA.

**Table:** 

Primers:	
*Sort1:*	*FW (ATCCACGTGTCAACAGACCA)*
	*Rev (CATGCATGAACACCATGTCA)*
*Add2:*	*FW (ACAAGGATGGAGGATAGTTCCCA)*
	*Rev (GCCTGTCCAGTCTCTGTCCT)*
*Srcin1:*	*FW (AGCGAGATGCGTTTATGGAC)*
	*Rev (GGTTGCTGCTCTCTCATCCT)*
*Shank2:*	*FW (AATGGTCGCTATCCCCGGAA)*
	*Rev (GTATCAGCTTTTGCCCCTCG)*
*Dnajc6:*	*FW (CGACCGTGGAAAAGGATCTA)*
	*Rev (AGGCCCCTTTTTGTCTTTGT)*
*Plk2:*	*FW (AGGATAGCACCATGGGAAGTGT)*
	*Rev (ACTGAAAGGACGTGCTGCFCAACTG)*
*Gapdh:*	*FW (GCCTTCTCTTGTGACAAAGTGGA)*
	*Rev (CCGTGGGTAGAGTCATACTGGAA)*

### Ribo(−) RNA‐sequencing

Library preparation and sequencing were performed by the Max Planck‐Genome‐Centre Cologne, Germany (http://mpgc.mpipz.mpg.de/home/) for compartmentalized RNA samples. For the first step, rRNA depletion (Ribo‐Zero rRNA removal Kit [Illumina]) has been performed with 1 μg total RNA per sample (*n* = 2 samples per condition), followed by library preparation with NEBNext Ultra™ Directional RNA Library Prep Kit for Illumina (New England Biolabs). Sequencing was performed as 100 bp single read sequencing on HiSeq2500™ (Illumina).

### Protein extraction

Primary hippocampal compartmentalized cultures were prepared as described earlier and treated at DIV19 with either 100 μM PTX or vehicle for 48 h. Inserts were then rinsed twice with ice‐cold phosphate buffered saline (PBS, Gibco™) and lysed using a cell scraper in home‐made RIPA‐buffer (150 mM NaCl, 1% Triton X‐100; 0.5% Sodium Deoxycholate; 1 mM EDTA; 1 mM EGTA; 0.05% SDS; 50 mM Tris pH 8) containing a protease inhibitor cocktail (1:1,000; Roche). Cell lysates were then homogenized by pipetting and centrifuged for 30 min at 13,000 *g* (4°C). The supernatant was snap‐frozen in liquid nitrogen and stored at −80°C until further use. Pierce™ BCA Protein Assay Kit (Thermo Scientific™) was used to quantify protein concentrations following the manufacturer's protocol. See Appendix for the method description of label‐free proteomics.

### Bioinformatic analysis

#### RNA‐Sequencing analysis

Adapter sequences were trimmed from the reads using Trimmomatic 0.38 with ILLUMINACLIP 1:30:10, LEADING:5, TRAILING:5, SLIDINGWINDOW:5:15 AVGQUAL:20 and MINLEN:30. The reads were then mapped to the rat genome Rnor 6.0 using Subread subjunc 1.33.16 and the Ensembl 96 transcript annotation, and quantified with featureCounts (v.1.6) (Liao *et al*, 2014). Counts were then aggregated to gene symbols and only genes with more than 20 reads in at least two samples for further processing.

Counts were then aggregated to gene symbols and only genes with more than 20 reads in at least two samples were considered for further processing.

Differential expression analysis was performed with edgeR (v. 3.28) (Robinson *et al*, 2010) using TMM normalization. Specifically, linear models were fitted using glmFit through all four conditions (∼ *compartment* * *treatment*), and differentially expressed genes were identified by testing for the *treatment* effect in each compartment using separate contrasts.

For the enrichment analyses, a compartment‐specific background of expressed genes was determined by taking only genes with more than 20 reads in at least two conditions within the respective compartment into account.

#### Proteomics data analysis

A comprehensive library of detected proteins was generated by combining a search against a *R*. *Norvegicus* reference proteome with exclusively reviewed entries (Swiss‐Prot, UP000002494, 8,104 proteins) with a search against a reference proteome containing also predicted entries (TrEMBL, UniProt UP000002494, 21,838 proteins) if proteins were undetectable in more than two samples. We detected 4,047 proteins with no missing values across all conditions out of the total 4,250 proteins. Values were first normalized by variance stabilizing transformation using the vsn package (version 3.54.0), and missing values were imputed using the MinProb function (*q*‐value < 0.01) of the DEP package (version 1.8.0).

The package limma (version 3.42.2) was used to perform differential expression analysis using a linear multiplicative model fitted through all four conditions (∼ *compartment* * *treatment*), and differential expression was computed using empirical Bayes statistics to contrast *treatment* and *compartment* effects.

#### GO enrichment analysis

Gene Ontology enrichment analysis was performed using the TopGo algorithm (v.2.40.0) (Alexa *et al*, 2006), essentially as described previously in (Lackinger *et al*, 2019).

For the compartment analysis (Fig 1D, Appendix Fig S1), significantly enriched genes with a logFC of greater than ± 1 were tested against the respective specific background. Subsequently, the Top 6/10 GO‐Terms in each ontology were plotted (with those filtered out that have more than 1,000 annotated genes in CC or more than 300 annotated genes in BP).

PTX GO‐terms were obtained by either testing exclusive gene sets (as characterized in Fig 2C) against the respective specific background (Fig 2D) or by testing all significantly down‐/upregulated genes in a specific compartment (Appendix Fig S6). For Fig 2D, the Top 5 obtained GO‐Terms of all three ontologies were sorted by *Enrichment* and of those, the Top 6 GO‐Terms plotted. GO‐Terms with more than 1,000 annotated genes in an ontology were filtered out.

GO‐Terms enriched in the proteomics dataset were identified by aggregating multiple detected protein isoforms and subsequently performing enrichment analysis of significantly changing proteins (criteria proteomics data analysis) with TopGo against the background of detected proteins. For plotting, GO‐terms with more than 500 annotated genes (CC) were filtered out.

#### miRNA‐binding site enrichment analyses

Predicted conserved MBS of rat conserved miRNA families (Targetscan 7.2) (Agarwal *et al*, 2015), were counted in exclusive gene sets as described in Fig 2C and tested for an enrichment against the specific background using a hypergeometric test on the number of binding sites (soon to be made available on Bioconductor).

We considered only those miRNAs, which are considerably expressed in hippocampal neurons at this developmental stage (expressed higher than the median of all detected miRNAs in Rajman *et al*, 2017).

#### 3′UTR length analysis

In order to get an estimate of the average 3′UTR lengths of genes present in somata and processes, genomic coordinates of the longest transcript associated with each detected gene were downloaded in R (AnnotationHub, Ensemble v.100, *Rattus Norvegicus*). As long 3′UTR isoforms are specifically prevalent in the brain (Miura *et al*, 2013), we chose to focus on these in our analysis.

Only 3′UTRs with a length of more than 20 nucleotides were considered for the comparisons.

#### RNA‐binding‐protein motif analysis

To identify enriched RBP motifs within 3′UTRs in differentially expressed genes, we extracted the matching FASTA sequences according to the criteria specified in the 3′UTR length analysis section. Sequences corresponding to differentially up‐/downregulated genes in each compartment upon PTX treatment were submitted to the AME‐tool of the meme‐suite (McLeay & Bailey, 2010) in combination with the CISBP database of known RBP motifs (Ray *et al*, 2013).

In AME, standard parameters were used with the *Average Odds Score* as scoring method and *Fisher*'*s exact test* for the enrichment estimation. RBP‐names in the plots were obtained by comparing the AME output motif_ID to the CISBP database motif_ID annotations.

#### Additional bioinformatical analyses

See Appendix for the method description of comparison to external dataset, Exon‐Intron split analysis (EISA) and analysis of half‐lives.

#### Graphical representations

Plots were generated using Affinity Designer, Cytoscape, BioRender.com & R (ggplot2 [v.3.3.1], ggpubr [v.0.4.0], ggsci [v.2.9] and ggrepel [v.0.8.1]).

#### Statistics

The number of independent experiments is indicated in the plots, if not explicitly specified differently in the methods section. Boxplots represent data as follows: central line: median; box: 25^th^ to 75^th^ percentile; whiskers: at most 1.5 times the interquartile range (IQR: distance between the 25^th^ to 75^th^ percentile); point outside: outliers outside the whiskers. Normality was tested using the Shapiro–Wilk test considering a *P*‐value under 0.05 non‐normally distributed. Normally distributed data were tested using one‐ or two sample Student's *t*‐test (always two‐sided) or ANOVA and otherwise for non‐normal data the non‐parametric counterpart tests Mann–Whitney *U*‐test or Kruskal–Wallis test. Differences in cumulative distributions were assessed by two‐sample Kolmogorov–Smirnow test. Bivariate correlation analysis between two variables was evaluated using the non‐parametric Spearman rank correlation or the parametric Pearson's correlation using the function ggscatter (from ggpubr).

Additional information about statistical tests not described in the figure legends are provided in the Appendix.

## Author contributions

DC performed proteomics, qPCR, Puromycin‐PLA‐experiments, RNA‐seq analysis and generated the figures. MR performed RNA‐seq. MS performed motif enrichment analysis. SB and MR performed and analysed the smFISH data. LvZ and JB supervised the proteomics experiments. JW performed electrophysiology experiments. CD helped with RNA‐seq data analysis. P‐LG supervised RNA‐seq and motif enrichment analysis. GS coordinated the project and wrote the manuscript.

## Conflict of interest

The authors declare that they have no conflict of interest.

## Supporting information



Appendix

Expanded View Figures PDF

## Data Availability

RNA‐seq data have been deposited to GEO (GSE155540): https://www.ncbi.nlm.nih.gov/geo/query/acc.cgi?acc=GSE155540 The mass spectrometry proteomics data have been deposited to the ProteomeXchange Consortium via the PRIDE partner repository (PXD020745): http://www.ebi.ac.uk/pride/archive/projects/PXD020745 The following figures have associated raw data: Figs 1, 2 and 4, Appendix Fig S1–S7. Selected data are available at https://dcolamethins.shinyapps.io/Compartment_PTX_App/.
