# Supplementary material for: Pervasive compartment‐specific regulation of gene expression during homeostatic synaptic scaling
Source: EMBO Rep. 2021 Aug 16;22(10):e52094. doi: 10.15252/embr.202052094 (PMC8490987; doi:10.15252/embr.202052094)
Supplement: Supplementary file 1 — Appendix [file EMBR-22-e52094-s001.pdf]

Appendix

Pervasive compartment-specific regulation of gene expression during homeostatic synaptic scaling

David Colameo[1,7]*, Marek Rajman[2*#], Michael Soutschek[1,7]*, Silvia Bicker[1,7], Lukas von Ziegler[3,7], Johannes Bohacek[3,7], Jochen Winterer[1, 7], Pierre-Luc Germain[4, 5], Christoph Dieterich[6] and Gerhard Schratt[1,7§]

Table of content:

Appendix Figure S1: Page 2
Appendix Figure S2: Page 3
Appendix Figure S3: Page 4
Appendix Figure S4: Page 5
Appendix Figure S5: Page 6
Appendix Figure S6: Page 7
Appendix Figure S7: Page 8
Appendix Figure S8: Page 9
Appendix Figure S9: Page 10
Appendix Figure S10: Page 11
Appendix Figure S11: Page 12
Appendix Figure Legends Pages 13 – 15
Supplementary Information on the Methods Section: Pages 16 – 26

Somata - MF

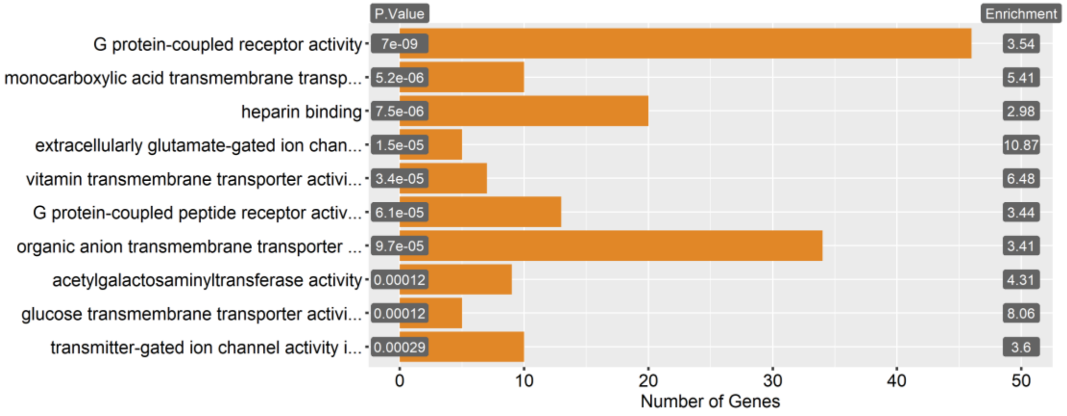

Processes - MF

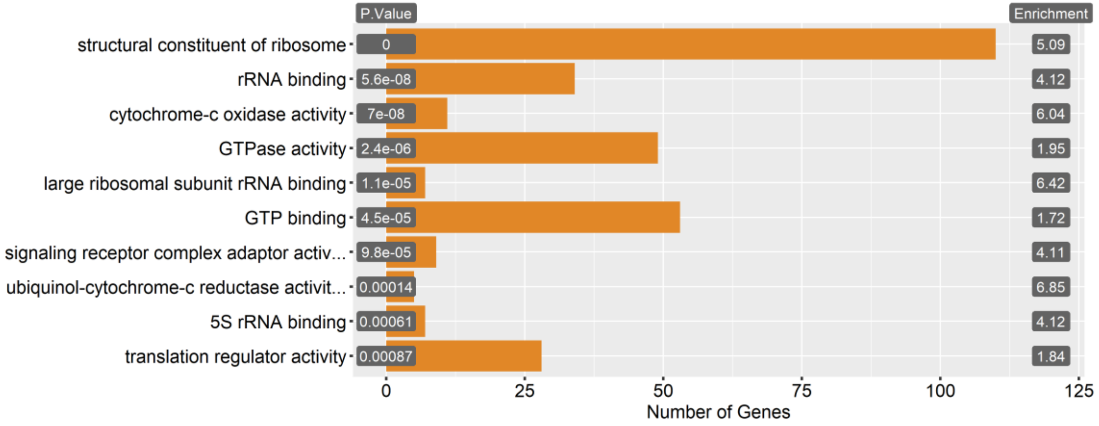

Somata - BP

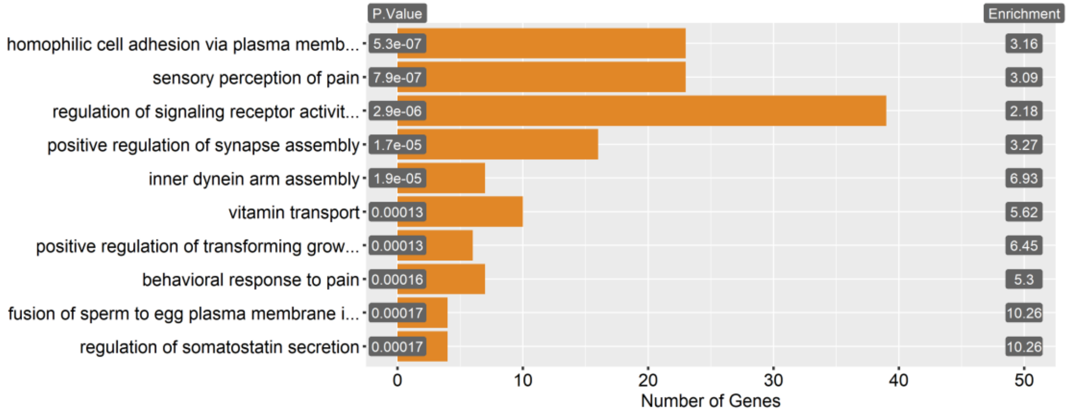

Processes - BP

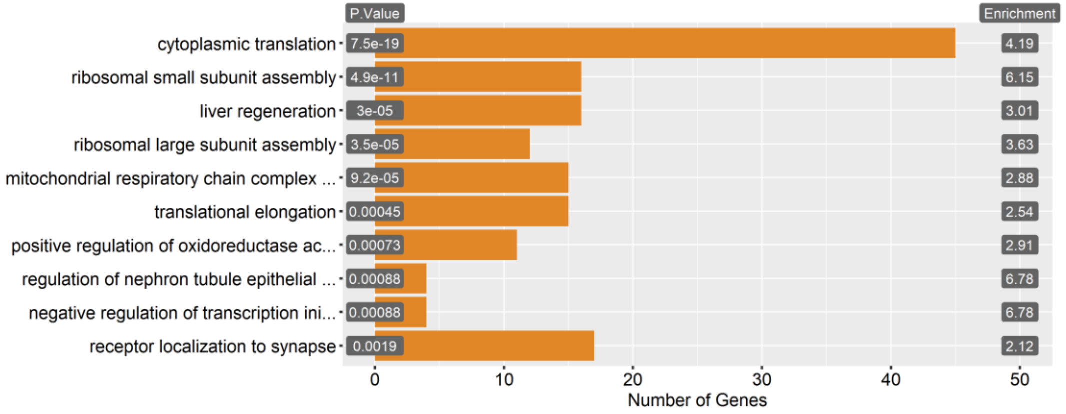

Appendix Fig. S1

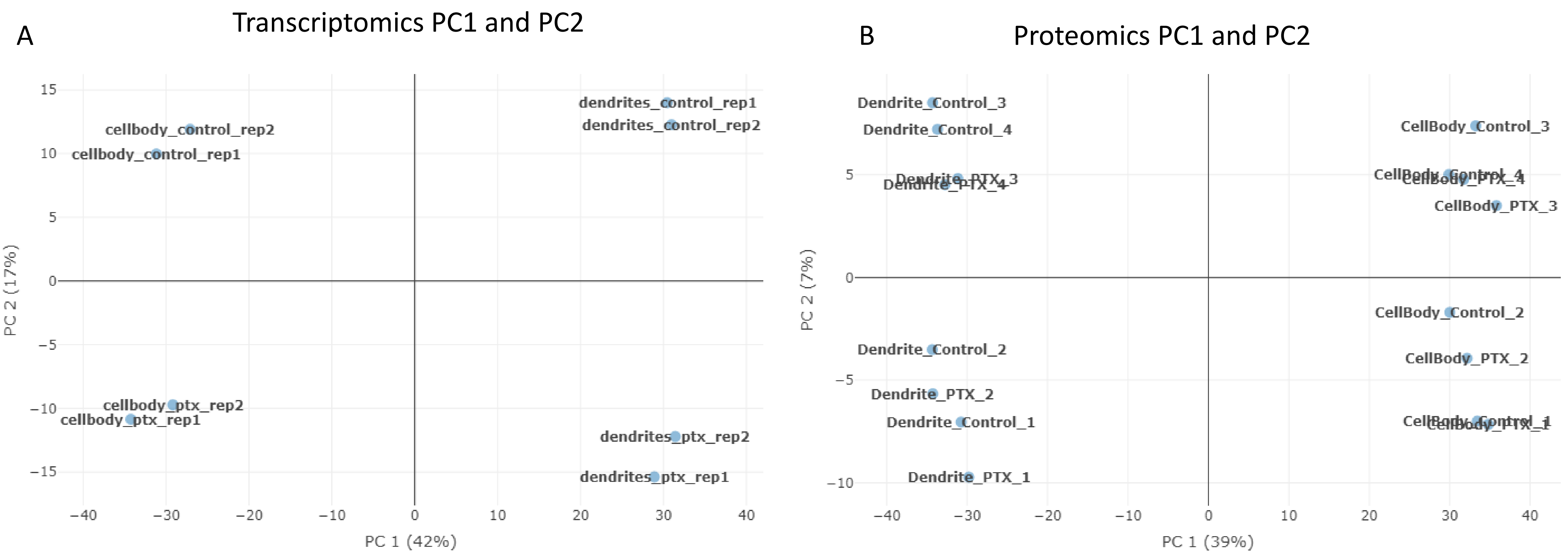

Appendix Fig. S2

lincRNAs

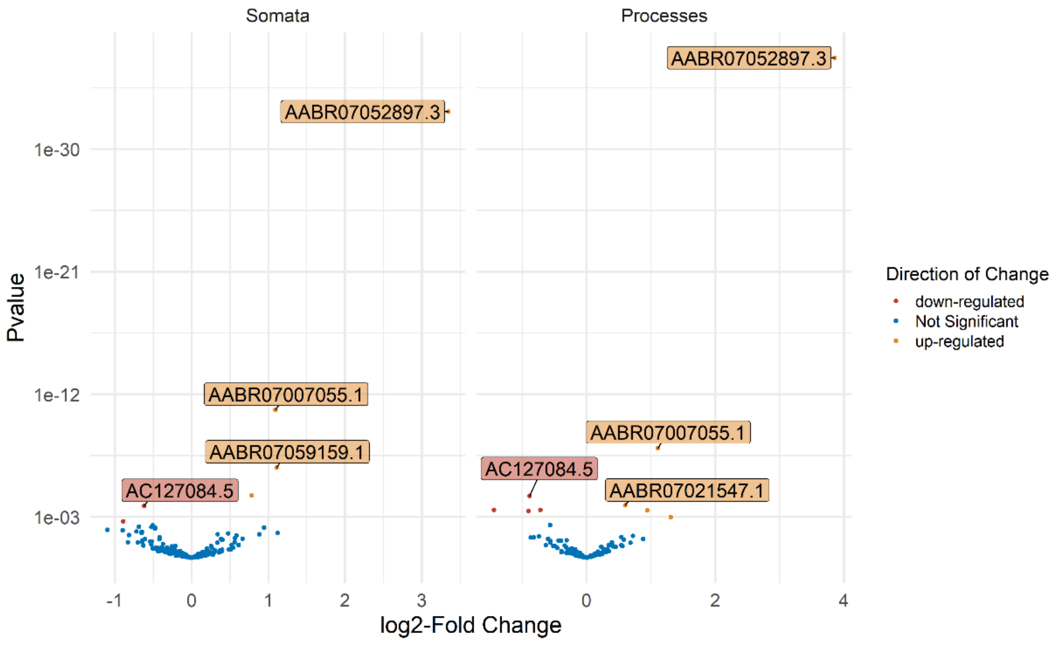

miRNA genes

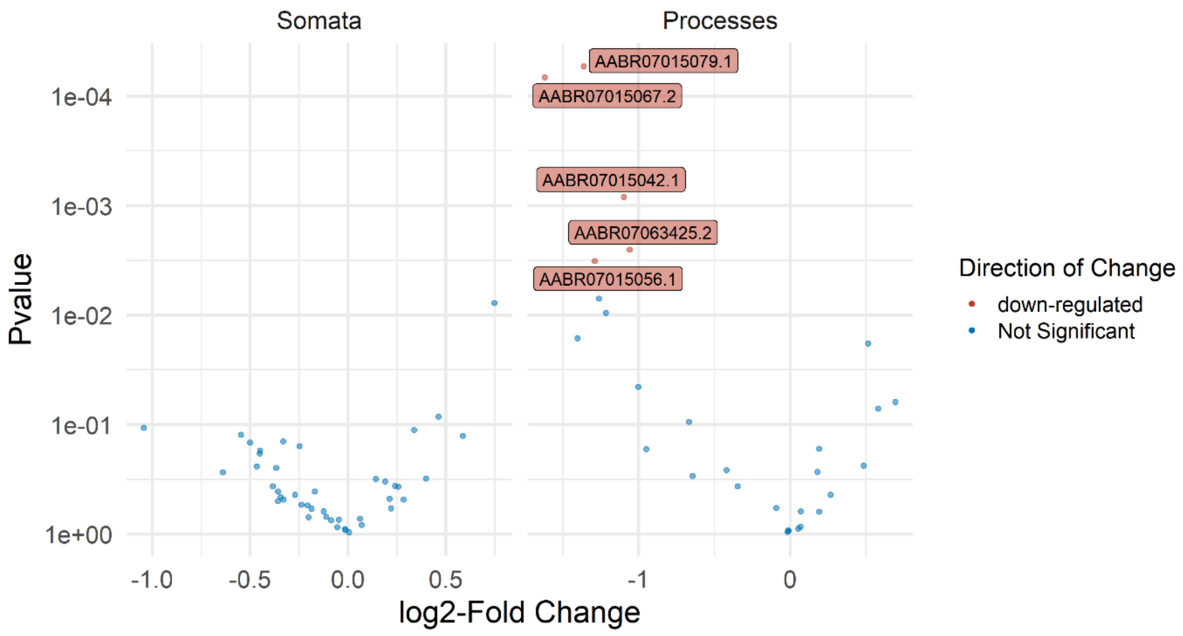

Appendix Fig. S3

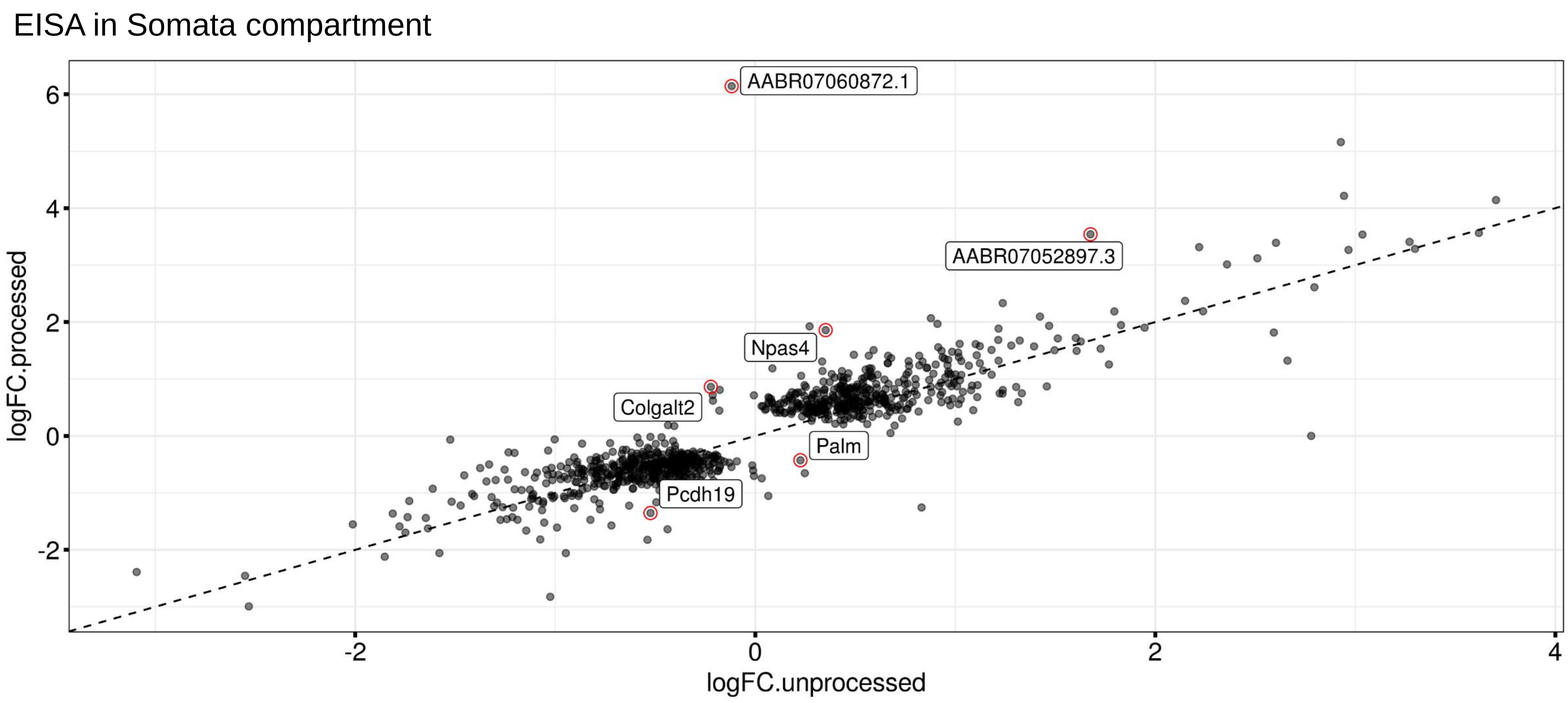

Appendix Fig. S4

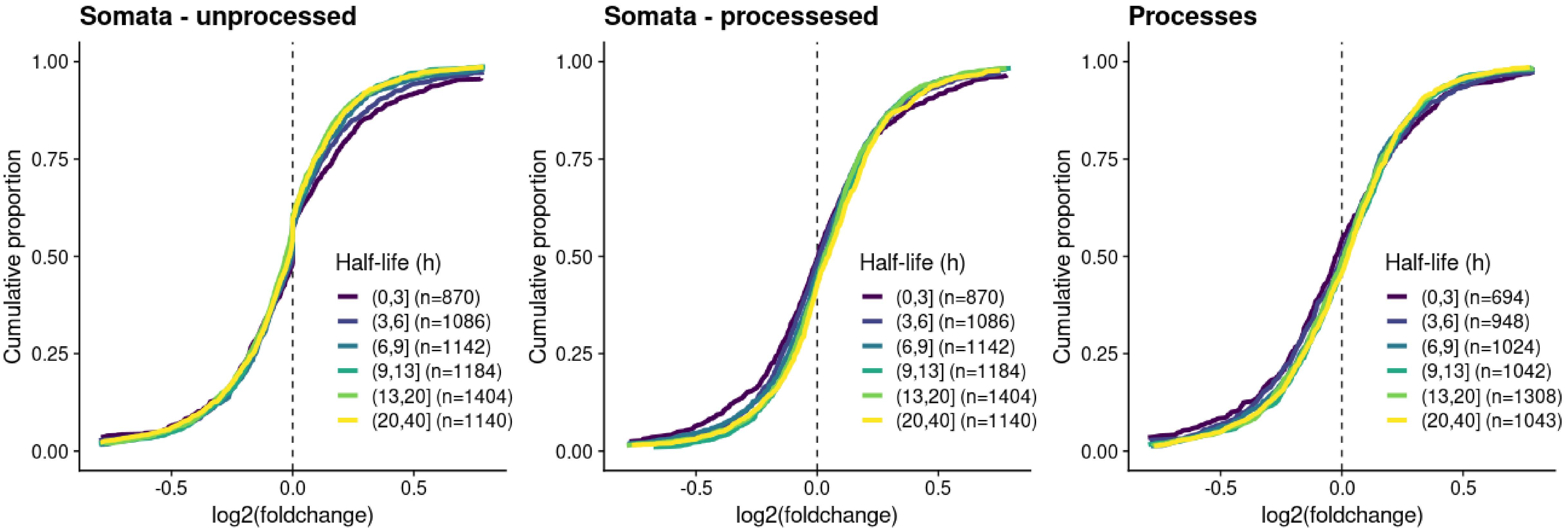

Appendix Fig. S5

Somata

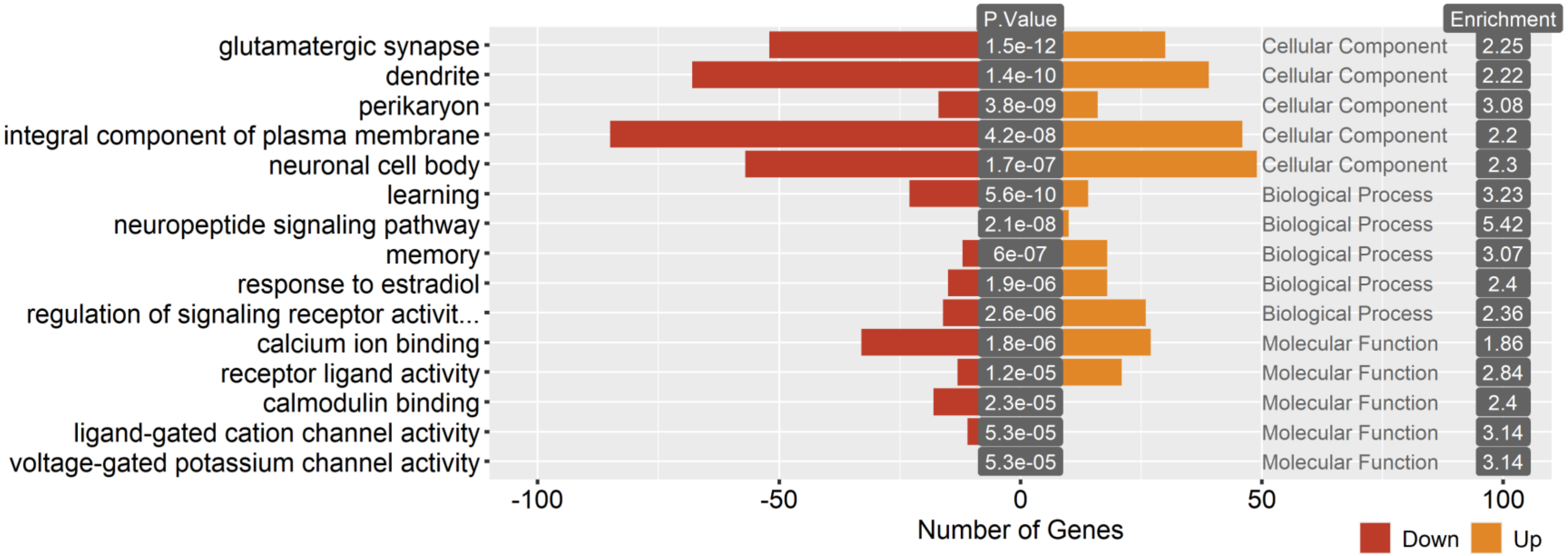

Processes

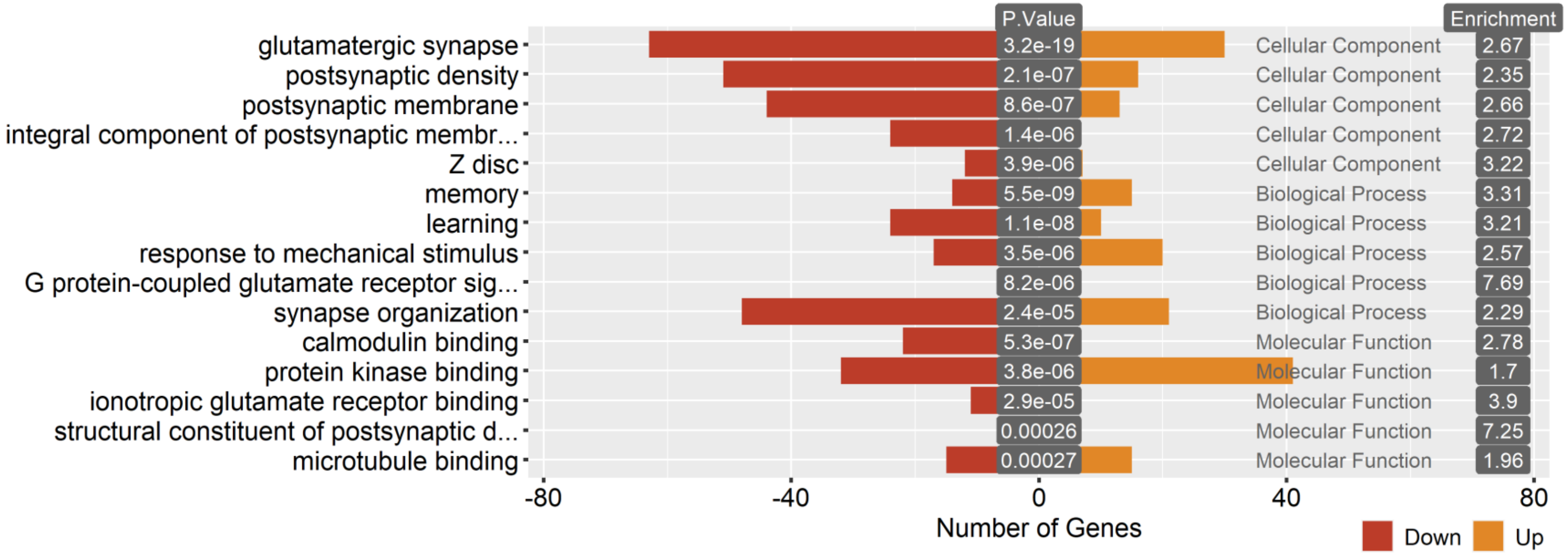

Appendix Fig. S6

Supplemental Figure to qPCR-Data

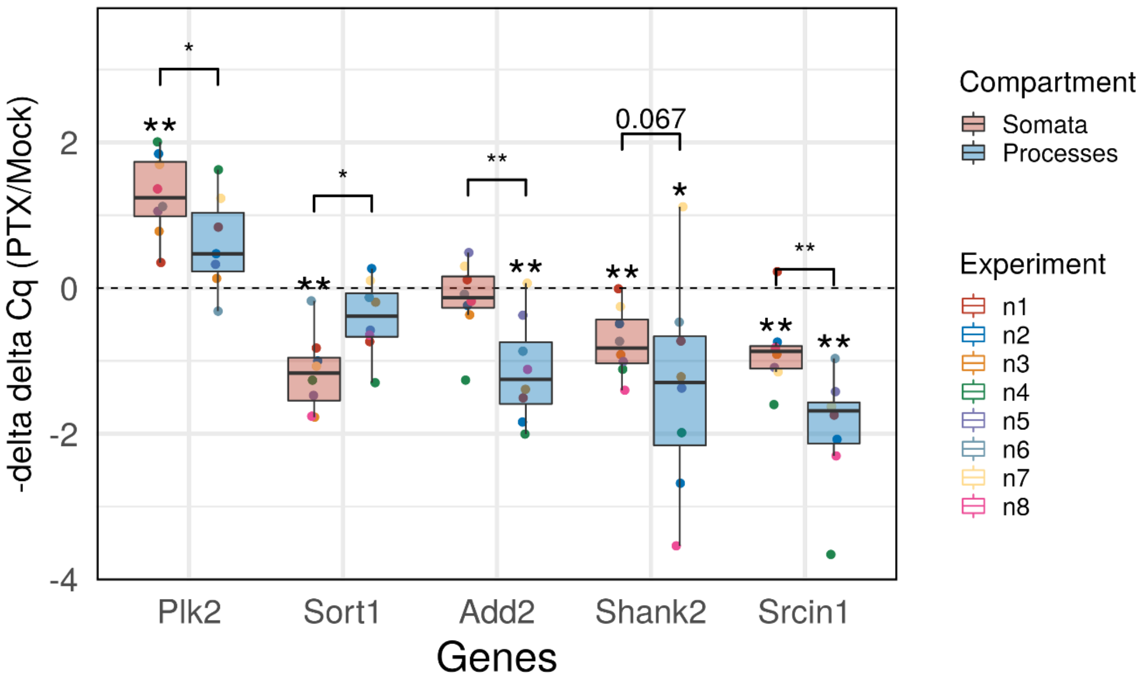

Supplemental Figure to FISH-Data

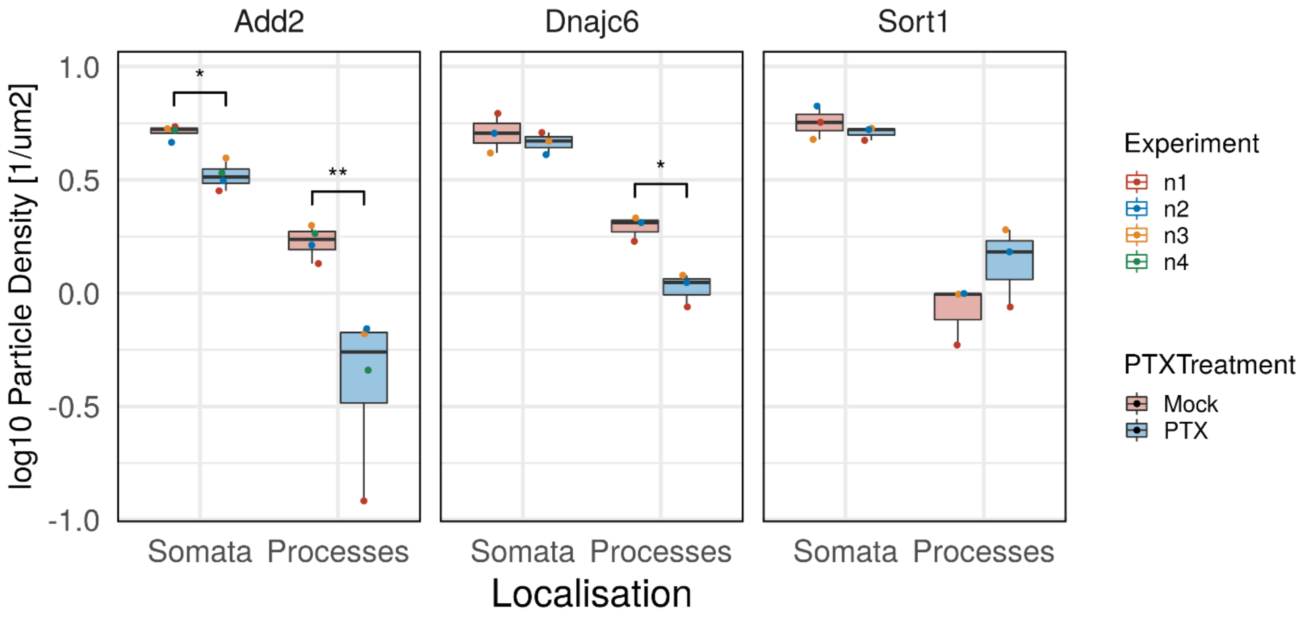

Appendix Fig. S7

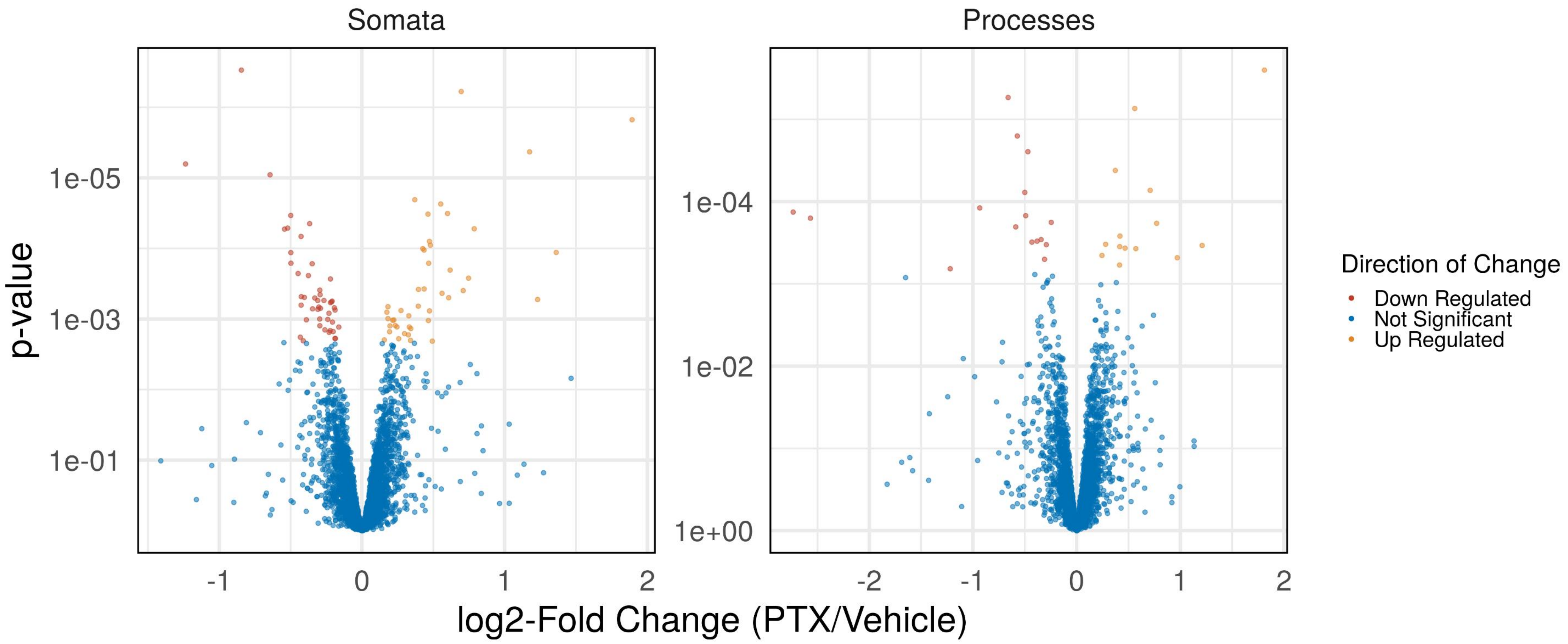

Appendix Fig. S8

Somata

Processes

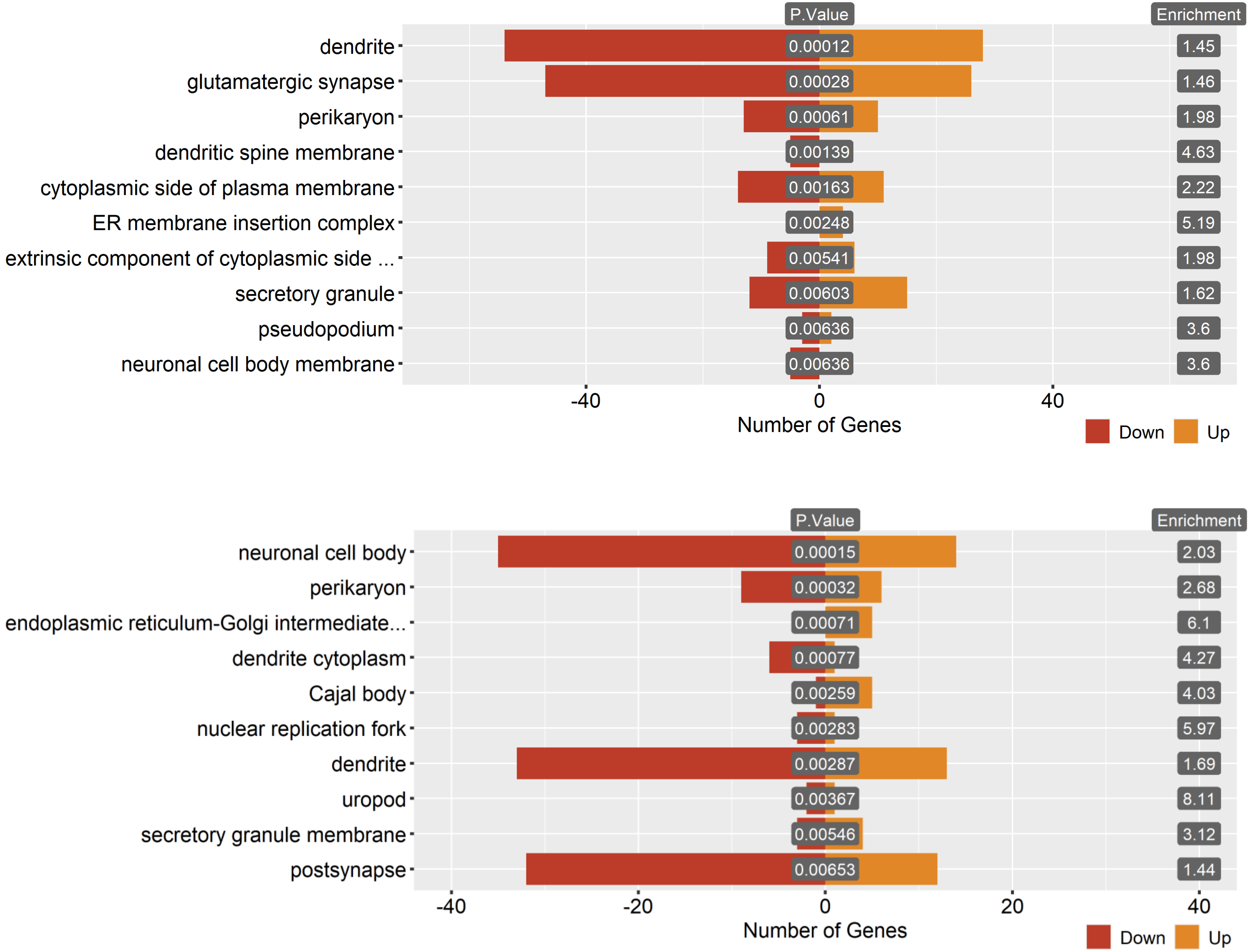

Appendix Fig. S9

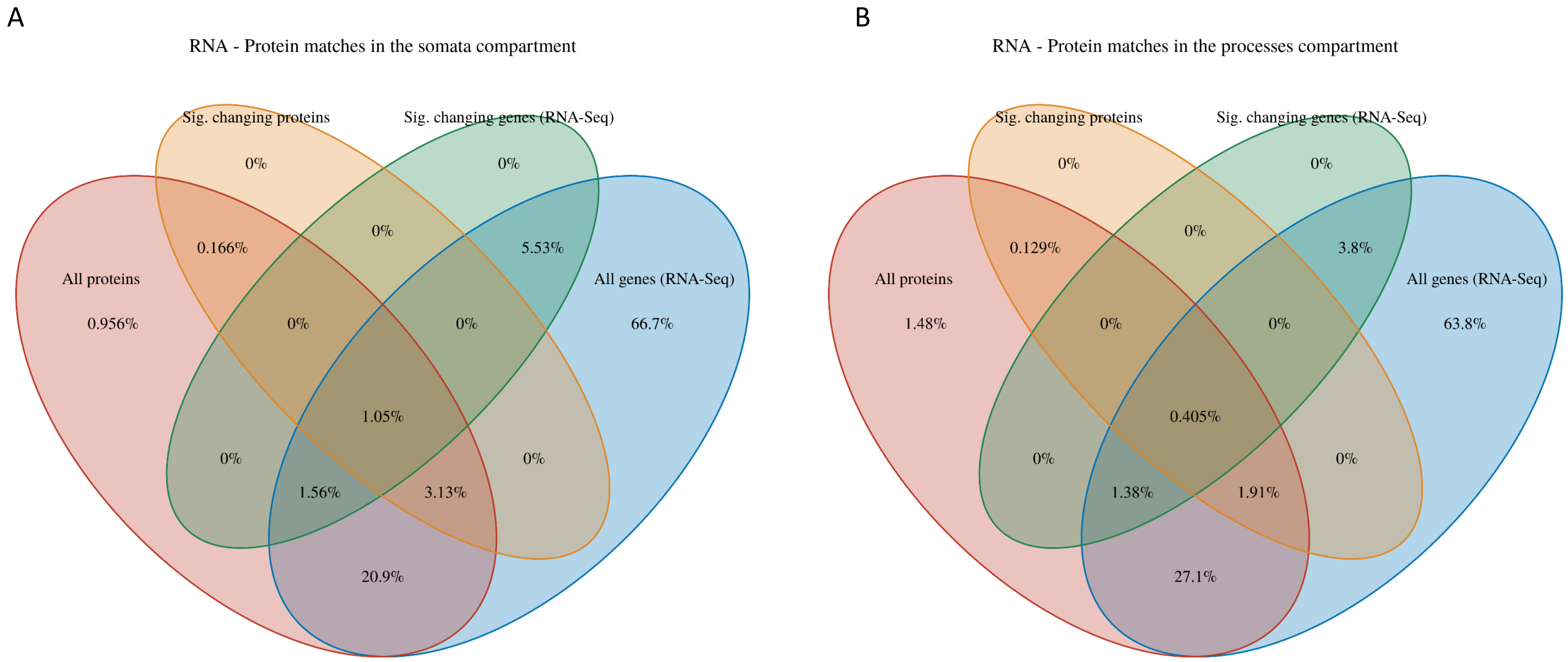

Appendix Fig. S10

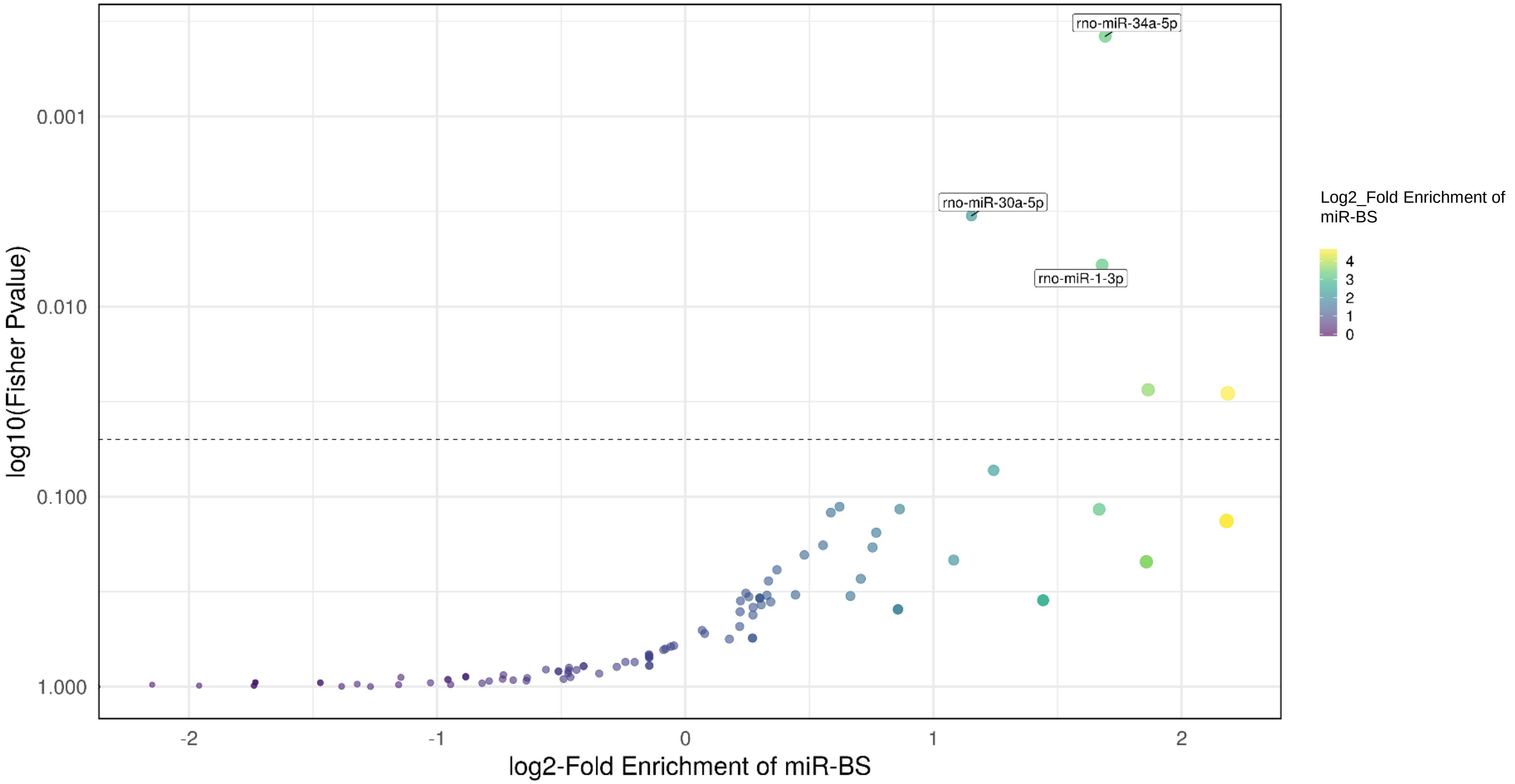

Appendix Fig. S11

Appendix Figure S1: GO-Term analysis of compartment-specific localized genes. Bar graphs illustrating the Top 10 gene ontology terms (MF = molecular function, BP = biological process) of genes enriched in the somata or processes compartment, respectively.

Appendix Figure S2: Principal component analysis of the transcriptomics (A) and proteomics (B) experiments.

Appendix Figure S3: lincRNA and miRNA associated genes are differentially expressed in both compartments upon PTX-treatment. Volcano Plots indicating changes in lincRNA and miRNA genes (Ensembl biotype classification) upon PTX treatment in the somata and processes compartment.

Appendix Figure S4: Exon-Intron split analysis (EISA) of the RNAs detected in the somata compartment by sequencing. Log-Fold-Changes of unprocessed RNAs (containing unspliced introns) are plotted versus the Fold-Changes of processed RNAs. Highlighted are likely candidates of which processed and unprocessed reads might be differentially regulated upon PTX treatment (see methods).

Appendix Figure S5: Distributions of log(foldchanges) upon PTX in unprocessed reads from the soma (A), processed reads from the soma (B), and processes. The genes were separated into groups depending on their half-lives (as estimated from Tushev et al.)

Appendix Figure S6: GO-Term analysis of genes changing upon PTX stimulation in the individual compartments. Depicted are the Top 5 terms of each gene ontology

(sorted by p-value, fisher-elim algorithm). Colored bar graphs indicate the number of significantly changing genes associated with each term in the respective compartment (yellow = sign. upregulated genes, red = sign. downregulated genes).

Appendix Figure S7: Supplemental Figures to Figure 3: (Left): Alternative representation of Real-time quantitative PCR (RT-qPCR) of transcripts changing differentially between compartments using the compartmentalized cultures after 48 hours PTX-treatment. n=7-8 independent biological replicates (Plk2 processes n7 missing due to insufficient material); PTX-effect per compartment was assessed by one-sample Student's t-test with $\mu_0=0$ multiple-test corrected using the Benjamin-Holm method; Differential compartment-effect was assessed by two-way ANOVA followed by Tukey's post-hoc multiple comparison test; $*p < 0.05$; $**p < 0.01$. Boxplots represent data as follows: central line: median; box: 25th to 75th percentile; whiskers: at most 1.5 times the inter-quartile range (IQR: distance between the 25th to 75th percentile); point outside: outliers outside the whiskers. (Right): Alternative representation of quantification of FISH-data shown in Figure 3B. n=3-4 independent biological replicates (Add2 was repeated a fourth time). PTX-effect was assessed by a three-way ANOVA followed by Tukey's post-hoc multiple comparison test; $*p < 0.05$; $**p < 0.01$).

Appendix Figure S8: Volcano plots representing proteins down- (red) or up-regulation (yellow) after 48 hours PTX in the somatic and process compartment. FDR < 0.1.

Appendix Figure S9: GO-Term analysis of significantly changing proteins upon PTX stimulation in individual compartments. Shown are the Top 10 significant GO-Terms of the cellular component ontology (sorted by p-value, fisher-elim algorithm) in both compartments. Colored bar graphs indicate the number of significantly changing

proteins associated with each term in the respective compartment (yellow = sign. upregulated proteins, red = sign. downregulated proteins).

Appendix Figure S10: Venn diagrams indicating the percentage of matches of RNA and protein changes upon PTX treatment in the somata (A) and processes (B) compartment.

Appendix Figure S11: miRNA binding site enrichment analysis of all significantly downregulated genes in the processes compartment.

Supplemental Information on the Method Section

Supplemental Information on the Method Section of the Manuscript “Pervasive compartment-specific regulation of gene expression during homeostatic synaptic scaling”., Colameo, Rajman and Soutschek et al.

Label-Free Proteomics

Protein digest and clean-up: Protein extracts were further processed with a filter assisted sample preparation protocol(Wisniewski et al., 2009). 20ug of protein were added to 30ul SDS denaturation buffer (4% SDS (w/v), 100mM Tris/HCL pH 8.2, 0.1M DTT). For denaturation, samples were incubated at 95°C for 5 min. Samples were diluted with 200ul UA buffer (8M urea, 100mM Tris/HCl pH 8.2) and then loaded to regenerated cellulose centrifugal filter units (Microcon 30, Merck Millipore, Billercia MA, USA). Samples were spun at 14000g at 35°C for 20 min. Filter units were washed once with 200ul of UA buffer followed by centrifugation at 14000g at 35°C for 15 min. Cysteines were alkylated with 100ul freshly prepared IAA solution (0.05M iodoacetamide in UA buffer) for 1 min at room temperature in a thermomixer at 600rpm followed by centrifugation at 14000g at 35°C for 10 min. Filter units were washed 3 times with 100ul of UA buffer then twice with a 0.5M NaCl solution in water (each washing was followed by centrifugation at 35°C and 14000g for 10 min). Proteins were digested overnight at room temperature with a 1:50 ratio of sequencing grade modified trypsin (0.4ug, V511A, Promega, Fitchburg WI) in 130ul TEAB (0.05M Triethylammoniumbicarbonate in water). After protein digestion over night at room temperature, peptide solutions were spun down at 14000g at 35°C for 15 min and acidified with 3ul of 20% TFA (trifluoroacetic acid).

Peptides Clean-up. Peptides were cleaned up using StageTip C18 silica columns (SP301, Thermo Scientific, Waltham MA). Columns were conditioned with 200ul methanol followed by 200 ul of 60% ACN (acetonitrile) / 0.1% TFA. Columns were equilibrated with 2 x 150 ul of 3% ACN / 0,1% TFA. Samples were loaded onto the columns. They were then washed with 2 x 150 ul 3% ACN / 0.1% TFA and eluted with 150 ul 60% ACN / 0.1% TFA. Samples were lyophilized in a speedvac then re-solubilized in 19ul 3% ACN / 0.1% FA (formic acid) prior to LC-MS/MS measurement. 1ul of synthetic peptides (Biognosys AG, Switzerland) were added to each sample for retention time calibration.

LC-MS/MS measurements:

Samples were measured on a QExactive (Thermo Fisher Scientific, Waltham MA, USA). Peptides were separated with an Eksigent NanoLC (AB Sciex, Washington, USA). We used a single-pump trapping 75-um scale configuration (Waters). 1ul of each were injected. Trapping was performed on a nanoEase^{TM} symmetry C18 column (pore size 100Å, particle size 5um, inner diameter 180um, length 20mm). For separation, a nanoEase^{TM} HSS C18 T3 column was used (pore size 100Å, particle size 1.8um, inner diameter 75um, length 250mm, heated to 50°C). Peptides were separated using a 120 min long linear solvent gradient of 5-35% ACN / 0.1% FA (using a flowrate of 300nl / min). Electronspray ionization with 2.6kV was used and a DIA method with a MS1 in each cycle followed by 35 fixed 20 Da precursor isolation windows within a precursor range of 400-1100 m/z was applied. For MS1 we used a maximum injection time of 200ms and an AGC target of 3e6 with a resolution of 60k in the range of 350-1500 m/z. MS2 spectra were acquired using a maximum injection time of 55ms an AGC target of 1e6 with a 30k resolution. A collision energy of 28 was used for fragmentation.

Protein search and quantification:

We used Spectronaut™ (Biognosys, version 10) with directDIA for peak picking and sequence assignment. We used a *M. musculus* reference proteome for *R. norvegicus* from uniprot (UP000002494). We included a maximum of 2 missed cleavages, using a Tryptic in-silico digest with a KR/P cutting profile. Sequences in a range of 7-52 AA were considered. We included carbamidomethyl as fixed modification for cysteine, oxidation as variable modification for methionine and protein N-terminal acetylation as variable modification. Decoys were generated using a scrambled label free decoy method. A normal distribution estimator was used with a 1% FDR for q-value filtering. A maximum of 5 variable modifications were considered. Single hit was determined on the stripped sequence level. Major grouping was done by protein group ID and minor grouping by stripped sequence. Only proteotypic peptide sequences were considered and single hit proteins excluded. For the minor and major group quantification, the top 3 entries were used using the mean precursor/peptide quantity. A localized normalization strategy and interference correction were used. Machine learning was performed on a per run basis and iRT profiling was enabled.

Comparison to external datasets

For the comparison of compartment localization of genes under basal conditions with external datasets, we merged the genes provided in the supplementary material of the metastudy by (von Kugelgen and Chekulaeva, 2020) with detected genes of the RNA-sequencing.

In brief, Kügelgen et al., (2020) calculate average log2 enrichments of genes significantly changed in 11 high coverage datasets (at least 5000 detected transcripts with a TPM > 10) describing neuronal localization in different species. For Figure EV2 D + E we implemented the ranking of genes most abundant in neurites as further provided by Kügelgen et al., (2020).

(Dorrbaum et al., 2020) performed SILAC on whole cells in the context of synaptic scaling to identify changes in protein synthesis and degradation as well as to determine activity dependent effects on protein abundances. We downloaded their supplemental data, including statistical analyses over three time points (1 day, 3 days and 7 days of treatment) and logFC of significantly changing proteins at each time point separately. We merged this data with the results obtained from label-free proteomics and filtered for significantly changing proteins (FDR < 0.5). We further excluded those which did not comprise an equivalent.

For the correlations with the 1 Day time point, we filtered for proteins changing at this time point in the specified analysis (log2FC $\neq$ 0).

Exon-Intron split analysis (EISA)

To quantify spliced (i.e. exonic) and unspliced transcripts, we aligned the reads using Rsubread subjunc (Liao, Smyth and Shi 2019). Subsequently, we quantified these alignments using featureCounts (Liao et al., 2014) with three sets of parameters: i) using exons as features (standard), ii) using exons with --nonSplitOnly, and iii) using transcripts as features with --nonSplitOnly (all three additionally shared the "-O --largestOverlap --nonOverlap 3 --fracOverlap 0.9 --primary -s 2" parameters). The first was used as a quantification of processed transcripts; for unprocessed transcripts, we subtracted from the third the counts of the second (which are compatible with processed transcripts as well). We went by conducting a differential expression analysis of the processed and unprocessed transcripts using edgeR (see RNA-sequencing analysis) with the following linear model in each compartment: ~ readtype * treatment. Since we don't expect meaningful unprocessed reads in the processes compartment, we focused in the further analysis on the somata compartment. We filtered for genes with more than 20 unprocessed reads in more than one sample and further narrowed the list with the following

parameters: i) FDR of changes upon PTX treatment in either processed or unprocessed reads < 0.01 and ii) a positive logCPM value. Genes that are in addition differently regulated upon PTX-treatment in both compartments (FDR processed reads somata & FDR processed reads processes < 0.05) were highlighted (Figure S4).

Differential Exon-Usage analysis (DEU)

We used the Bioconductor package diffUTR 0.99.30 (Gerber et al., 2021) in order to assess the differential usage of specific exons or 3'UTRs upon PTX treatment in either of the two compartments. In brief, bins were prepared based on Rattus Norvegicus Ensembl version 103 and alternative poly-Adenylation information downloaded from polyA-DB (and lifted over to rn6) (https://exon.apps.wistar.org/PolyA_DB/v3/download/3.2/rat_pas.zip) and quantified from the aforementioned subjunc alignments as described in the package. Differential usage was conducted with the DEXSeq 1.36.0 (Reyes et al., 2013) wrapper, gene-level aggregations was performed separately for UTRs and CDSs.

Liao Y, Smyth GK, Shi W (2019). "The R package Rsubread is easier, faster, cheaper and better for alignment and quantification of RNA sequencing reads." *Nucleic Acids Research*, **47**, e47. doi: 10.1093/nar/gkz114.

Anders S, Reyes A, Huber W (2012). "Detecting differential usage of exons from RNA-seq data." *Genome Research*, **22**, 4025. doi: 10.1101/gr.133744.111.

Analysis of RNA half-lives

To estimate mRNA half-lives, we re-analyzed the 3'-seq data upon transcriptional blocking from Tushev et al. Reads were aligned to the Rnor6 genome using STAR and splicing junctions from the Ensembl 99 transcriptome. Read ends were then clustered up to a minimum gap of 12nt to identify distinct sites, which were annotated to the nearest upstream gene (with max 10kb distance to the nearest exon). To normalize

read counts across samples, TMM normalization was first applied, and samples were then expressed as a ratio to the timepoint 0 of the corresponding replicate, and the 20 most stable transcripts were assumed not to be degraded to calculating normalization factors. An exponential decay model (i.e. `y~e^(-lambda*time)` using Levenberg-Marquardt non-linear least square fitting. Transcripts for which the standard error of the lambda was greater than the value of the lambda, or for which the significance of the lambda coefficient was above 0.05, were excluded from further analyes. Finally, to estimate gene-level half-lives, we calculated a weighted sum of transcript-level half-lives, with the transcripts' CPM as weights.

Additional information about statistical tests not described in the figure legends:

Figure 1B: Two Sample Mann-Whitney-U test to test difference in amplitude:

		W-Statistics	Mean1	Mean2	CI Low	CI High	P-Value
Mock	PTX	81	21.723	17.536	0.59	7.82	5.20E-05

Figure 3A: Three-way ANOVA (~ Compartment * PTX-Treatment * Gene + Experiment) with Tukey's post-hoc test

	Df	Sum Sq	Mean Sq	F value	Pvalue
Compartment	1	21.6	21.6	52.8	2.94E-11
PTXTreatment	1	14.2	14.2	34.7	3.10E-08
Genes	4	305	76.4	186	4.76E-53
Experiment	7	6.61	0.944	2.3	0.0302
Compartment:PTXTreatment	1	2.96	2.96	7.23	0.00812
Compartment:Genes	4	46.5	11.6	28.4	5.52E-17
PTXTreatment:Genes	4	25	6.25	15.3	2.90E-10
Compartment:PTXTreatment:Genes	4	4.37	1.09	2.66	0.0353
Residuals	131	53.7	0.41		

contrast	Compartment	Genes	estimate	SE	df	t.ratio	p.value
Mock - PTX	Somata	Plk2	-1.28	0.32	131	-3.99	0.000109
Mock - PTX	Processes	Plk2	-0.616	0.342	131	-1.8	0.0742
Mock - PTX	Somata	Sort1	1.17	0.32	131	3.65	0.000383
Mock - PTX	Processes	Sort1	0.401	0.32	131	1.25	0.213
Mock - PTX	Somata	Add2	0.154	0.32	131	0.48	0.632
Mock - PTX	Processes	Add2	1.13	0.32	131	3.53	0.000584
Mock - PTX	Somata	Shank2	0.74	0.32	131	2.31	0.0223
Mock - PTX	Processes	Shank2	1.36	0.32	131	4.25	4.10E-05
Mock - PTX	Somata	Srcin1	0.863	0.32	131	2.7	0.00789
Mock - PTX	Processes	Srcin1	1.93	0.32	131	6.02	1.65E-08

Figure 3B: Two-way ANOVA (~ Compartment * Gene) with Tukey's post-hoc test

	DF	Sum	Mean Square	F-Value	P-Value
Gene	2	9.244	4.622	18.633	1.13E-04
Compartment	1	1.479	1.479	5.964	2.85E-02
Gene:Compartment	2	4.578	2.289	9.229	2.78E-03
Residual	14	3.473	0.248		

Gene	Group1	Group2	Estimate	CI Low	CI High	p.adj	p.adj.signif
Add2	Somata	Processes	-1.43	-2.67	-0.195	0.0299	*
Sort1	Somata	Processes	-0.755	-0.12	-0.305	0.00957	**
Dnajc6	Somata	Processes	-0.853	0.301	1.41	0.0128	*

Figure 5C: Three-way ANOVA to model PTX effect (~ PTX-Treatment * Localisation * Antibody + Experiment) followed by a Tukey's post-hoc analysis to contrast the PTX-Effect within compartment and peptide of interest.

	Df	Sum Sq	Mean Sq	F value	Pr(>F)	
PTXTreatment	1	0.115	0.115	27.019	1.35E-04	***
Localisation	1	0.796	0.796	186.900	1.73E-09	***
Antibody	1	0.127	0.127	29.842	8.36E-05	***
Experiment	2	0.297	0.148	34.873	3.65E-06	***
PTXTreatment:Localisation	1	0.015	0.015	3.488	0.083	
PTXTreatment:Antibody	1	0.005	0.005	1.077	0.317	
Localisation:Antibody	1	0.084	0.084	19.747	0.001	***
PTXTreatment:Localisation:Antibody	1	0.000	0.000	0.001	0.973	
Residuals	14	0.060	0.004			

contrast	Localisation	Antibody	estimate	SE	df	t.ratio	p.value	
Mock - PTX	Somata	Camk2a	0.117	0.053	14.000	2.201	0.045	*
Mock - PTX	Processes	Camk2a	0.215	0.053	14.000	4.035	0.001	**
Mock - PTX	Somata	Syn1	0.060	0.053	14.000	1.129	0.278	
Mock - PTX	Processes	Syn1	0.161	0.053	14.000	3.031	0.009	**

Figure 5D: Three-way ANOVA to model the puromycin-treatment (~Puro-Treatment * Localisation * Antibody + PTX-Treatment + Experiment) followed by a Tukey's post-hoc analysis.

	Df	Sum Sq	Mean Sq	F value	Pr(>F)	
PuroTreatment	1	0.800	0.800	80.059	2.88E-09	***
Localisation	1	1.311	1.311	131.103	1.95E-11	***
Antibody	1	0.056	0.056	5.553	0.027	*
PTXTreatment	1	0.115	0.115	11.504	0.002	**
Experiment	2	0.420	0.210	21.000	4.45E-06	***
PuroTreatment:Localisation	1	0.006	0.006	0.550	0.465	
PuroTreatment:Antibody	1	0.081	0.081	8.074	0.009	**
Localisation:Antibody	1	0.091	0.091	9.152	0.006	**
PuroTreatment:Localisation:Antibody	1	0.006	0.006	0.554	0.464	
Residuals	25	0.250	0.010			

contrast	Localisation	Antibody	estimate	SE	df	t.ratio	p.value	
PuroMinus - PuroPlus	Somata	Camk2a	-0.486	0.074	25.000	-6.605	6.40E-07	***
PuroMinus - PuroPlus	Processes	Camk2a	-0.486	0.074	25.000	-6.603	6.43E-07	***
PuroMinus - PuroPlus	Somata	Syn1	-0.233	0.074	25.000	-3.160	0.00410	**
PuroMinus - PuroPlus	Processes	Syn1	-0.338	0.074	25.000	-4.588	1.08E-04	***

Figure 6B: Two-sample Kolmogorov-Smirnov Test (Down-regulated vs Up-regulated in both compartments)

			D-Statistics	P-Value
Somata	Down-regulated	Up-regulated	0.09606	0.0438
Processes	Down-regulated	Up-regulated	0.073309	0.2447

Figure 6E: Mann-Whitney U-Test (Ratio in Somata vs Ratio in Processes)

		W-Statistics	Mean1	Mean2	CI Low	CI High	P-Value
Ratio Somata	Ratio Processes	1985	0.37	0.783	-0.666	-0.160	5.20E-05

Appendix Figure S7 (Left): Two-way ANOVA (~ *Compartment* * *Genes* + *Experiment*) with Tukey's post-hoc test

	Df	Sum Sq	Mean Sq	F value	Pr(>F)
Compartment	1	5.92	5.92	13.5	0.000505
Genes	4	50	12.5	28.4	1.98E-13
Experiment	7	9.04	1.29	2.94	0.01
Compartment:Genes	4	8.82	2.2	5.02	0.00144
Residuals	62	27.2	0.439		

contrast	Genes	estimate	SE	df	t.ratio	p.value
Somata - Processes	Plk2	0.724	0.344	62	2.1	0.0396
Somata - Processes	Sort1	-0.766	0.331	62	-2.31	0.0241
Somata - Processes	Add2	0.974	0.331	62	2.94	0.00461
Somata - Processes	Shank2	0.619	0.331	62	1.87	0.0667
Somata - Processes	Srcin1	1.06	0.331	62	3.21	0.00213

One-sample t-test (μ_0=0) to test for PTX-Effect and multiple-testing corrected using the Benjamin-Holm method

Genes	Compartment	estimate	statistic	p.value	parameter	conf.low	conf.high	P.adjusted
Plk2	Somata	1.28	6.42	0.000361	7	0.807	1.75	0.00143
Plk2	Processes	0.616	2.45	0.0499	6	0.000445	1.23	0.0624
Sort1	Somata	-1.17	-6.24	0.000428	7	-1.61	-0.725	0.00143
Sort1	Processes	-0.401	-2.22	0.0621	7	-0.828	0.0266	0.069
Add2	Somata	-0.154	-0.817	0.441	7	-0.598	0.291	0.441
Add2	Processes	-1.13	-4.48	0.00287	7	-1.72	-0.532	0.00479
Shank2	Somata	-0.74	-4.5	0.00279	7	-1.13	-0.351	0.00479
Shank2	Processes	-1.36	-2.7	0.0308	7	-2.55	-0.167	0.0441
Srcin1	Somata	-0.863	-4.71	0.00219	7	-1.3	-0.43	0.00479
Srcin1	Processes	-1.93	-6.75	0.000264	7	-2.6	-1.25	0.00143

Appendix Figure S7 (Right): Two-way ANOVA (~ *Compartment* * *Gene* + *Experiment*) with Tukey's post-hoc test

	Df	Sum Sq	Mean Sq	F value	Pr(>F)
Localisation	1	4.22	4.22	251	1.50E-14
PTXTreatment	1	0.342	0.342	20.4	0.000132
Gene	2	0.186	0.093	5.54	0.0102
Experiment	3	0.133	0.0444	2.64	0.0713
Localisation:PTXTreatment	1	0.067	0.067	3.99	0.0568
Localisation:Gene	2	0.0629	0.0314	1.87	0.175
PTXTreatment:Gene	2	0.419	0.209	12.5	0.000176
Localisation:PTXTreatment:Gene	2	0.207	0.104	6.17	0.00663
Residuals	25	0.42	0.0168		

contrast	Localisation	Gene	estimate	SE	df	t.ratio	p.value
Mock - PTX	Somata	Add2	0.193	0.0917	25	2.1	0.0457
Mock - PTX	Processes	Add2	0.624	0.0917	25	6.81	3.87E-07
Mock - PTX	Somata	Dnajc6	0.0419	0.106	25	0.396	0.696
Mock - PTX	Processes	Dnajc6	0.269	0.106	25	2.54	0.0176
Mock - PTX	Somata	Sort1	0.0449	0.106	25	0.424	0.675
Mock - PTX	Processes	Sort1	-0.212	0.106	25	-2	0.0562
