# Supplementary material for: Pervasive compartment‐specific regulation of gene expression during homeostatic synaptic scaling
Source: EMBO Rep. 2021 Aug 16;22(10):e52094. doi: 10.15252/embr.202052094 (PMC8490987; doi:10.15252/embr.202052094)
Supplement: Supplementary file 2 — Expanded View Figures PDF [file EMBR-22-e52094-s002.pdf]

Expanded View Figures

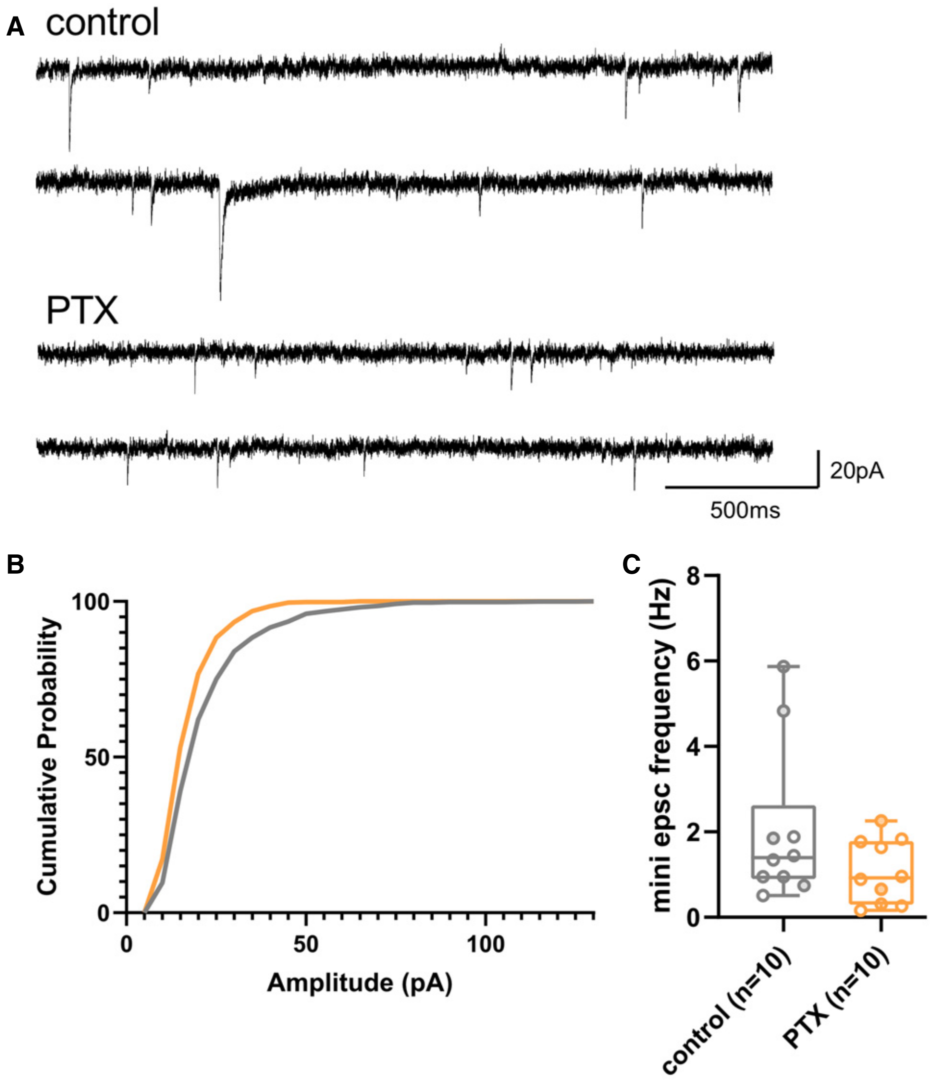

Figure EV1. Additional data on electrophysiological experiments.

A Example traces of a mock- and PTX-treated neuron.

B Cumulative distribution of all miniature EPSC events of mock- (grey line) and PTX-treated (orange line) neurons.

C Quantification of miniature EPSC frequency (Hz) presented as a boxplot (n = 10 neurons from three independent biological replicates). Boxplots: central line: median; box: 25th to 75th percentile; whiskers: until last data point within 1.5× interquartile range (IQR).

© 2021 The Authors

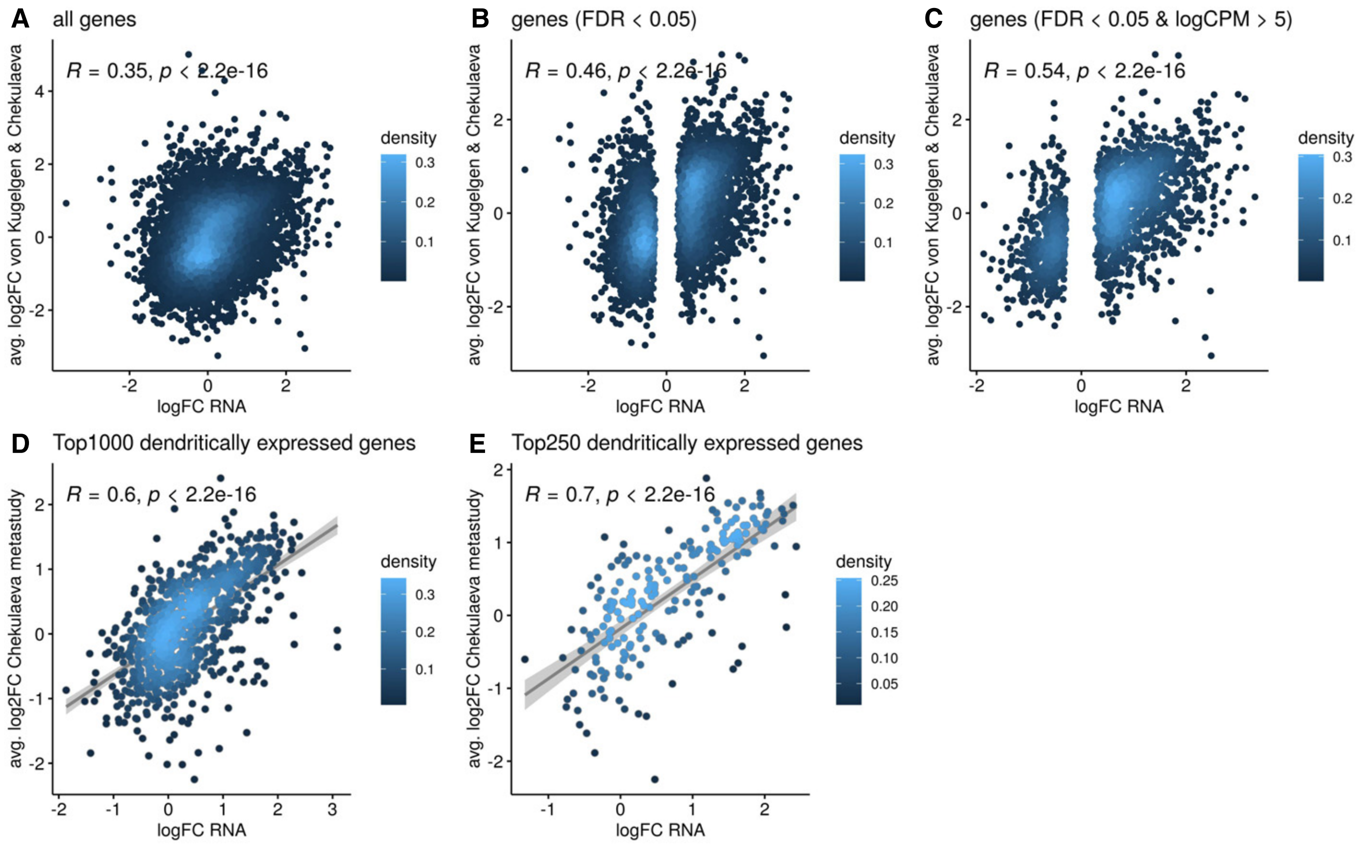

Figure EV2. Subsumption of compartment-specific gene location with a recent meta-study under basal conditions.

Pearson's correlation of RNA logFC (Processes vs Somata) under basal conditions to the average of significant $\log_2$ enrichments of genes as of obtained by (von Kugelgen & Chekulaeva, 2020) from 11 high coverage datasets describing neuronal localization. The statistical significance of Pearson correlations was calculated in the standard fashion, i.e. by testing against a null hypothesis of $r = 0$ using a t-distribution with N-2 degrees of freedom.

A Correlation with all genes detected in our study.

B Subset of significantly enriched or depleted genes in the two compartments (FDR < 0.05).

C Subset of significantly changing and highly expressed genes in the two compartments (FDR < 0.05 and logCPM > 5).

D, E Local enrichment correlation between a subset of the most abundant genes detected in neurites as classified by Kugelgen and Chekulaeva (2020) to genes detected in the compartment sequencing. Regression lines indicate fitted linear models, with the light grey shaded areas depicting the 95% confidence interval.

Data information: Coloured is in each case the X-Y density.

 © 2021 The Authors

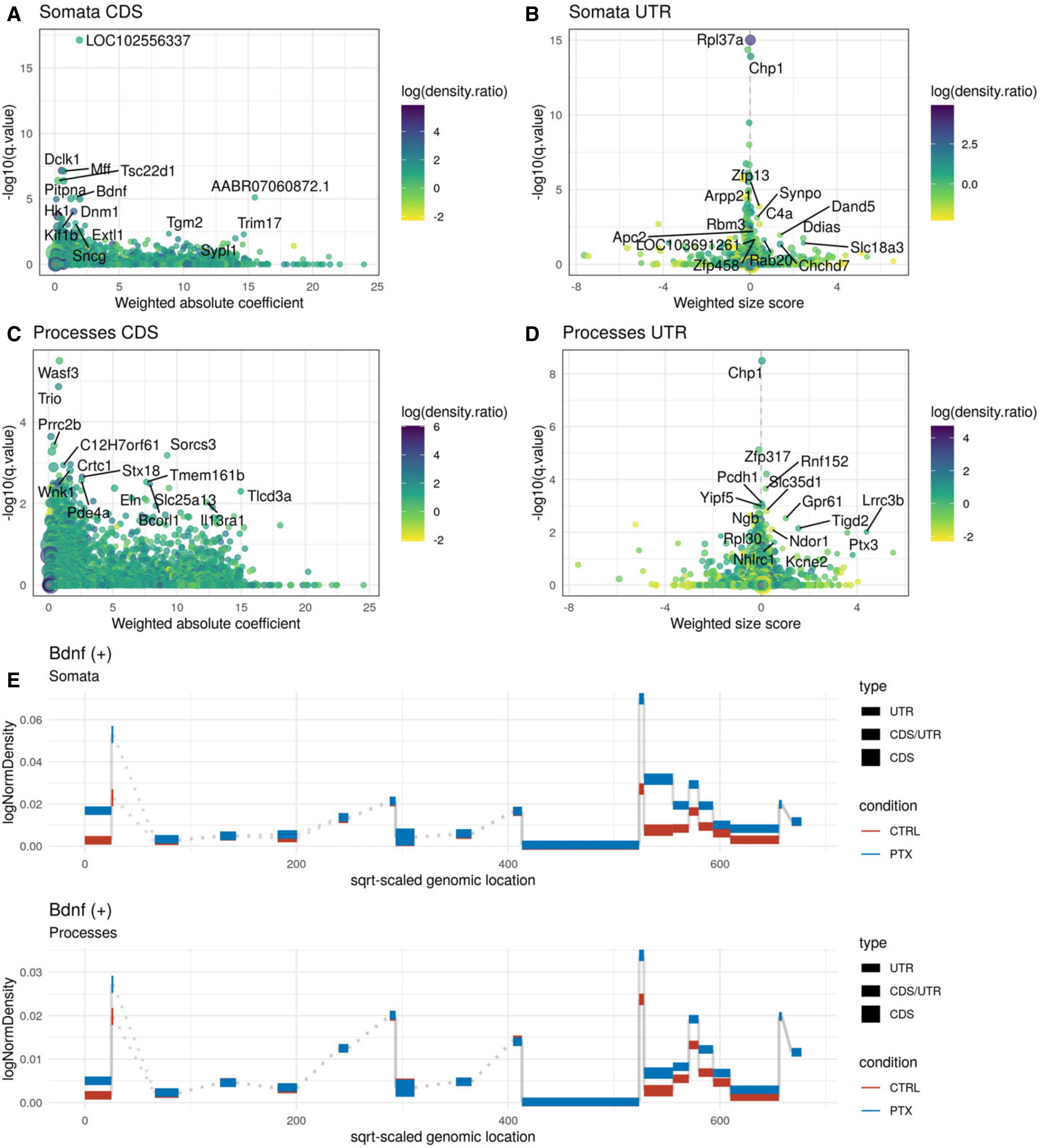

Figure EV3. Differential exon-usage (DEU) analysis upon PTX treatment in the somata and processes compartment.

A–D Gene-Level statistic plots of the DEU analysis in each compartment, separately for the coding sequence (CDS, left) and 3′UTR (right), with the top 15 candidates labelled. Gene-level estimates of effect sizes (bin-level coefficients weighted by significance) are displayed on the *x*-axis in CDS plots (A + C), whereas the weighted size score in 3′UTR plots (B + D) includes a further length weighing (see Gerber *et al* (2021) for further details).

E Bin-level plot displaying different exon-usage upon PTX treatment in both compartments for the example gene Bdnf.

© 2021 The Authors

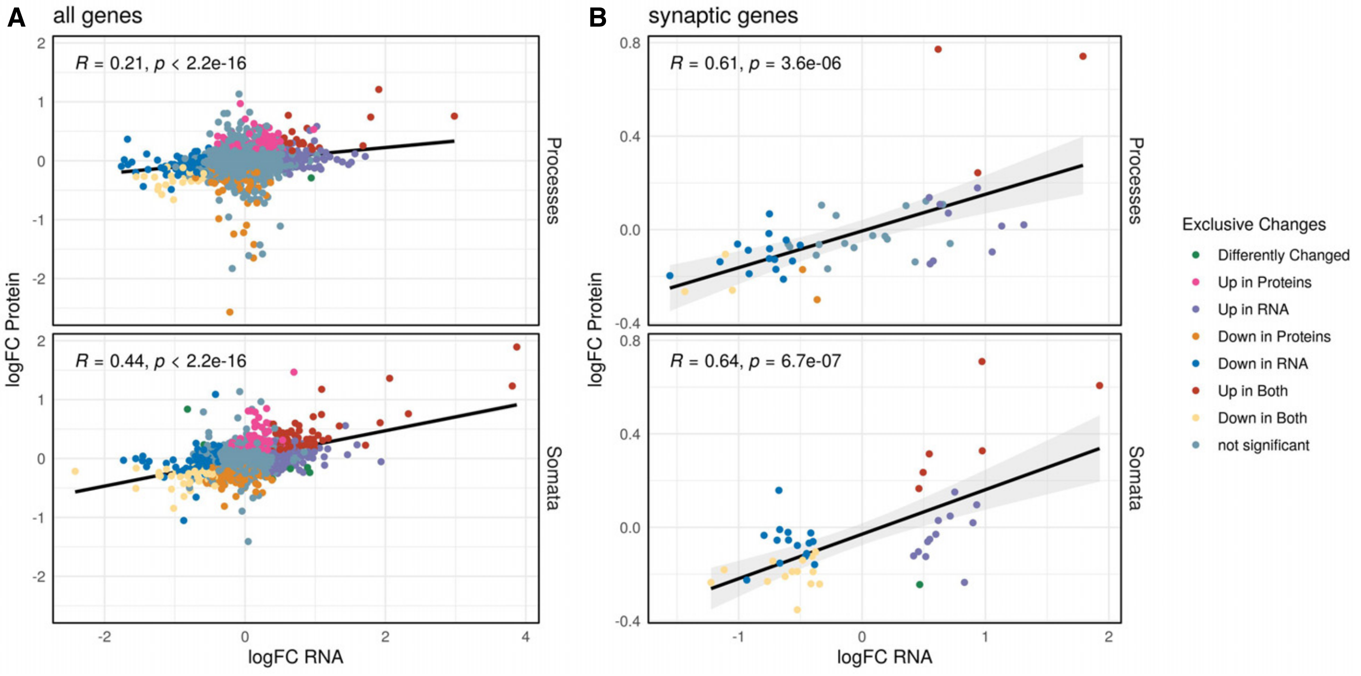

Figure EV4. Correlation of changes in RNA and Protein abundance upon PTX treatment.

Pearson's correlation of RNA- and Protein logFC upon PTX treatment in both compartments. The statistical significance of Pearson correlations was calculated in the standard fashion, i.e. by testing against a null hypothesis of $r = 0$ using a t-distribution with N-2 degrees of freedom. Regression lines indicate fitted linear models, with the light grey shaded areas depicting the 95% confidence interval.

A Correlation with all genes detected in both assays.

B Subset of only synaptic genes (see Fig 6). Coloured are the changes in both compartments (RNA significance with FDR < 0.05, Protein significance with FDR < 0.5).

 © 2021 The Authors

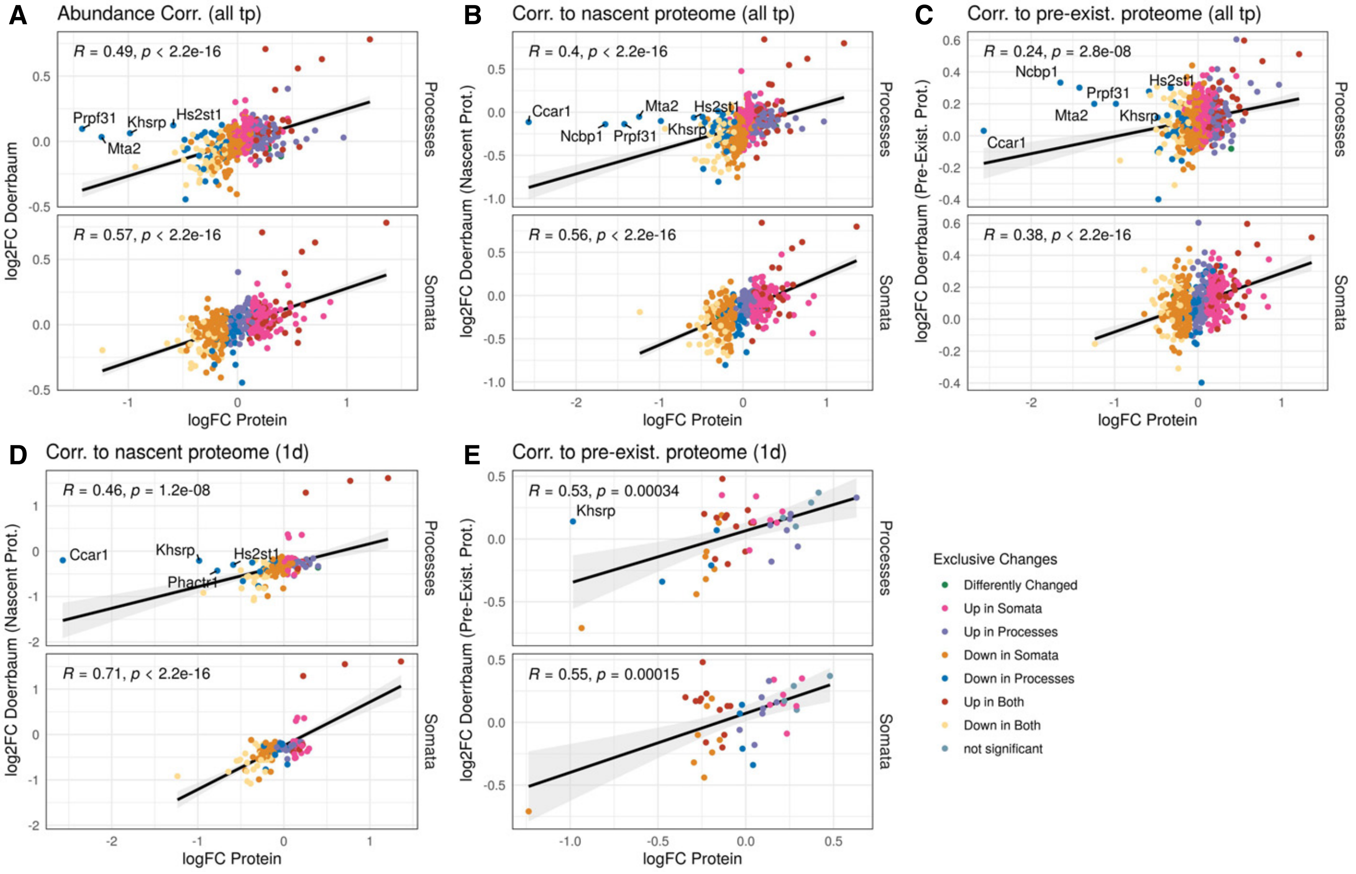

Figure EV5. Comparison of PTX effects on protein changes to the dynamics published in Dorrbaum *et al* (2020).

Pearson's correlation of significantly changing proteins (FDR < 0.5) upon PTX treatment in both compartments to protein dynamics determined by a whole cell SILAC experiment subsequent bicuculline treatment (Dorrbaum *et al*, 2020). The statistical significance of Pearson correlations was calculated in the standard fashion, i.e. by testing against a null hypothesis of $r = 0$ using a t-distribution with N-2 degrees of freedom. Regression lines indicate fitted linear models, with the light grey shaded areas depicting the 95% confidence interval. Proteins significantly downregulated in the processes compartment with a logFC of less than −0.5 are highlighted in the processes panel.

- A Correlation with changes in protein abundance as determined by Dörrbaum and colleagues upon bicuculline treatment over the time period of 7 days (three time points of protein collection).
- B Correlation with changes of the nascent (newly synthesized) proteome over the 7 days' time course.
- C Correlation with changes of the pre-existing proteome over the 7 days' time course.
- D Correlation with changes of the nascent proteome seen by Dorrbaum *et al* (2020) after the time point of 1 day.
- E Correlation with changes of the pre-existing proteome at the 1-day time point.

© 2021 The Authors
