# Supplementary material for: Pervasive compartment‐specific regulation of gene expression during homeostatic synaptic scaling
Source: EMBO Rep. 2021 Aug 16;22(10):e52094. doi: 10.15252/embr.202052094 (PMC8490987; doi:10.15252/embr.202052094)
Supplement: Supplementary file 3 [file EMBR-22-e52094-s003.pdf]

Pervasive compartment-specific regulation of gene expression during homeostatic synaptic scaling

David Colameo, Marek Rajman, Michael Soutschek, Silvia Bicker, Lukas von Ziegler, Johannes Bohacek, Jochen Winterer, Pierre-Luc Germain, Christoph Dieterich, and Gerhard Schratt
DOI: 10.15252/embr.202052094

Corresponding author(s): Gerhard Schratt (gerhard.schratt@hest.ethz.ch)

Review Timeline:

Transfer from Review Commons:	16th Nov 20
Editorial Decision:	18th Nov 20
Revision Received:	26th Apr 21
Editorial Decision:	23rd Jun 21
Revision Received:	12th Jul 21
Accepted:	20th Jul 21

Editor: Esther Schnapp

Transaction Report: This manuscript was transferred to EMBO reports following peer review at Review Commons.

(Note: With the exception of the correction of typographical or spelling errors that could be a source of ambiguity, letters and reports are not edited. Depending on transfer agreements, referee reports obtained elsewhere may or may not be included in this compilation. Referee reports are anonymous unless the Referee chooses to sign their reports.)

1st Editorial Decision **18th Nov 2020**

Dear Gerhard,

Thank you for the submission of your manuscript with referee reports to EMBO reports. I have now read your point by point response, and I am happy to invite you to revise your study along the lines you suggest for publication here.

I would thus like to invite you to revise your manuscript with the understanding that the referee concerns must be fully addressed and their suggestions taken on board. Please address all referee concerns in a complete point-by-point response. Acceptance of the manuscript will depend on a positive outcome of a second round of review. It is EMBO reports policy to allow a single round of major revision only and acceptance or rejection of the manuscript will therefore depend on the completeness of your responses included in the next, final version of the manuscript.

Revised manuscripts should be submitted within three months of a request for revision; they will otherwise be treated as new submissions. Please contact us if a 3-months time frame is not sufficient for the revisions so that we can discuss this further. Please note that the EMBO reports reference style has changed to Harvard style, this needs to be corrected.

Regarding data quantification, please specify the number "n" for how many independent experiments were performed, the bars and error bars (e.g. SEM, SD) and the test used to calculate p-values in the respective figure legends. This information must be provided in the figure legends. Please also include scale bars in all microscopy images.

IMPORTANT NOTE: we perform an initial quality control of all revised manuscripts before re-review. Your manuscript will FAIL this control and the handling will be DELAYED if the following APPLIES:
1) A data availability section providing access to data deposited in public databases is missing. If you have not deposited any data, please add a sentence to the data availability section that explains that.
2) Your manuscript contains statistics and error bars based on n=2. Please use scatter blots in these cases. No statistics should be calculated if n=2.

When submitting your revised manuscript, please carefully review the instructions that follow below. Failure to include requested items will delay the evaluation of your revision.

1) a .docx formatted version of the manuscript text (including legends for main figures, EV figures and tables). Please make sure that the changes are highlighted to be clearly visible.

2) individual production quality figure files as .eps, .tif, .jpg (one file per figure).
See https://wol-prod-cdn.literatumonline.com/pb-assets/embo-site/EMBOPress_Figure_Guidelines_061115-1561436025777.pdf for more info on how to prepare your figures.

3) We replaced Supplementary Information with Expanded View (EV) Figures and Tables that are collapsible/expandable online. A maximum of 5 EV Figures can be typeset. EV Figures should be cited as 'Figure EV1, Figure EV2" etc... in the text and their respective legends should be included in the main text after the legends of regular figures.

- For the figures that you do NOT wish to display as Expanded View figures, they should be

bundled together with their legends in a single PDF file called *Appendix*, which should start with a short Table of Content. Appendix figures should be referred to in the main text as: "Appendix Figure S1, Appendix Figure S2" etc. See detailed instructions regarding expanded view here:

- Additional Tables/Datasets should be labeled and referred to as Table EV1, Dataset EV1, etc. Legends have to be provided in a separate tab in case of .xls files. Alternatively, the legend can be supplied as a separate text file (README) and zipped together with the Table/Dataset file.

4) a .docx formatted letter INCLUDING the reviewers' reports and your detailed point-by-point responses to their comments. As part of the EMBO Press transparent editorial process, the point-by-point response is part of the Review Process File (RPF), which will be published alongside your paper.

5) a complete author checklist, which you can download from our author guidelines . Please insert information in the checklist that is also reflected in the manuscript. The completed author checklist will also be part of the transparent RPF.

6) Please note that all corresponding authors are required to supply an ORCID ID for their name upon submission of a revised manuscript (). Please find instructions on how to link your ORCID ID to your account in our manuscript tracking system in our Author guidelines

7) Before submitting your revision, primary datasets produced in this study need to be deposited in an appropriate public database (see https://www.embopress.org/page/journal/14693178/authorguide#datadeposition). Please remember to provide a reviewer password if the datasets are not yet public. The accession numbers and database should be listed in a formal "Data Availability" section placed after Materials & Method (see also https://www.embopress.org/page/journal/14693178/authorguide#datadeposition). Please note that the Data Availability Section is restricted to new primary data that are part of this study. * Note - All links should resolve to a page where the data can be accessed. *
If your study has not produced novel datasets, please mention this fact in the Data Availability Section.

8) We would also encourage you to include the source data for figure panels that show essential data. Numerical data should be provided as individual .xls or .csv files (including a tab describing the data). For blots or microscopy, uncropped images should be submitted (using a zip archive if multiple images need to be supplied for one panel). Additional information on source data and instruction on how to label the files are available at .

9) Our journal also encourages inclusion of *data citations in the reference list* to directly cite datasets that were re-used and obtained from public databases. Data citations in the article text are distinct from normal bibliographical citations and should directly link to the database records from which the data can be accessed. In the main text, data citations are formatted as follows: "Data ref: Smith et al, 2001" or "Data ref: NCBI Sequence Read Archive PRJNA342805, 2017". In the Reference list, data citations must be labeled with "[DATASET]". A data reference must provide the database name, accession number/identifiers and a resolvable link to the landing page from which the data can be accessed at the end of the reference. Further instructions are available at https://www.embopress.org/page/journal/14693178/authorguide#referencesformat

We would also welcome the submission of cover suggestions, or motifs to be used by our Graphics

Illustrator in designing a cover.

As part of the EMBO publication's Transparent Editorial Process, EMBO reports publishes online a Review Process File (RPF) to accompany accepted manuscripts. This File will be published in conjunction with your paper and will include the referee reports, your point-by-point response and all pertinent correspondence relating to the manuscript.

You are able to opt out of this by letting the editorial office know (emboreports@embo.org). If you do opt out, the Review Process File link will point to the following statement: "No Review Process File is available with this article, as the authors have chosen not to make the review process public in this case."

I look forward to seeing a revised version of your manuscript when it is ready. Please let me know if you have questions or comments regarding the revision.

Kind regards,
Esther

Esther Schnapp, PhD
Senior Editor
EMBO reports

***
Rev_Com_number: RC-2020-00437
New_manu_number: EMBOR-2020-52094V1
Corr_author: Schratt
Title: Pervasive compartment-specific regulation of gene expression during homeostatic synaptic scaling

Point-by-point response to the reviewers' comments (our responses in red)

Reviewer #1 (Evidence, reproducibility and clarity (Required)):

In the current study, Colameo, Schratt et al. exploited a multi-omics approach using RNA-seq and mass spectrometry to address homeostatic synaptic scaling in primary neurons. To allow for compartment-specific resolution, the authors used filter inserts that separate cell body from neuronal protrusions. For both, RNA and protein, Colameo et al. found numerous alterations in expression levels. Moreover, they observed compartment-specific changes. Among coding genes, the authors also detected differentially expressed miRNAs that might contribute to regulate synaptic scaling.

Major concern:

Understanding synaptic scaling at the molecular level is of utmost importance to unravel regulatory pathways that are essential to control neuronal excitability. In this manuscript, the authors addressed this question using RNA-seq and mass spec. First, the approach of using filter inserts is not novel (Poon & Martin, 2006, J. Neurosci. PMC6675000). Second, several papers have been published that have addressed homeostatic synaptic scaling in primary neurons using mass spectrometry and RNA seq, also neuronal compartment-specific (Dörrbaum et al. 2020, Elife; Schanzenbächer et al. 2018 Elife; Schanzenbächer et al. 2016, Neuron; Tushev 2018 Neuron). Consequently, the authors have to compare their results in detail with the cited studies and provide explicitly, which facts are indeed novel and how their results might also differ from the other studies. In the moment, the manuscript does not provide novel insight into the process of synaptic scaling. Also, functional and mechanistic insight is largely lacking and, if at all, mostly correlative.

We strongly disagree with this reviewer that our approach of using filter inserts in combination with transcriptomics/proteomics to unravel compartment-specific changes in gene expression during synaptic scaling is not novel. Indeed, the process of synaptic scaling has not been studied in a compartment-specific manner before as evidenced by a lack of respective publications. The papers by the Schuman group mentioned by this reviewer do not address compartment-specific regulation. Dörrbaum et al. (2020) and Schanzenbächer et al (2016, 2018) use different proteomics approaches in cultured neurons without taking into account different compartments. They also do not provide any transcriptome analysis. In Tushev et al (2018), 3'-end RNA-sequencing is used to study 3'UTR diversity, also in a compartmentalized fashion using hippocampal neuropil dissection. However, synaptic downscaling, which is typically observed >6h after enhanced activity, is not addressed in this paper.
Nevertheless, as suggested by this reviewer, **we have now compared our datasets to Schanzenbächer et al. (2016, 2018) and Dörrbaum et al. (2020)** to investigate the degree of overlap between our study and these studies at the proteomic level. In the revised manuscript, we focused on the comparison to Dörrbaum et al. (2020), since the experimental setup of this study is most comparable to ours. However, if needed, we would of course be happy to also provide the comparisons we obtained for Schanzenbächer et al., 2016&2018. The main conclusions derived from these comparisons **(new Fig. EV5)** are summarized as follows:

1. In general, there is a **good correlation** between the fold-changes in protein levels obtained by both studies, arguing that the results are reproducible and relevant in the context of synaptic downscaling.
2. Correlations are generally **stronger if we compare our data to nascent proteins** rather than pre-existing proteins, arguing that most of the changes we observe originate from alterations in de novo protein synthesis, which is also consistent with the strong correlation between RNA and protein fold-changes.
3. Correlations are generally **stronger if we use our somatic** rather than process **dataset** for comparison, arguing that with our compartmentalized approach, we increase resolution and are now able to monitor process-specific alterations much more robustly. For example, a number of strongly downregulated genes in our dataset (e.g. Ccar1, Khsrp, Ncbp1) are only marginally affected when investigating at the entire neuron level.

We further are highly convinced that our manuscript provides important new insight into the process of synaptic downscaling. We show for the first time that a large fraction of neuronal proteins are controlled in a highly compartmentalized fashion. This is particularly relevant, since synaptic scaling was long assumed to be regulated exclusively at the level of entire neurons or even neural networks. Moreover, we found that these compartment-specifically regulated proteins belong to discrete functional groups, with postsynaptic proteins often being regulated locally in the synapto-dendritic compartment, consistent with dendritic enrichment of the respective protein-encoding mRNAs.
Concerning mechanistic insight, we provide first evidence from bioinformatics analysis that RBP and microRNA regulation are specifically involved in the regulation of gene expression in the different compartments, with a disproportionally high contribution of microRNAs to the post-transcriptional downregulation of synaptic genes in dendrites. In our opinion, further mechanistic experiments are beyond the scope of this resource article.

Specific points:

-Fig. 1: Endogenous neuronal activity is a prerequisite for synaptic scaling and for the interpretation of their RNA-seq and mass spec data. Can the authors provide electrophysiological data that the neurons they investigated showed indeed endogenous activity?

The picrotoxin model of synaptic downscaling in primary hippocampal neurons has been widely used by us and other groups in the past (e.g. Seeburg et al., Neuron 2008; Fiore et al., EMBO J. 2014; Rajman et al., EMBO J. 2017). Nevertheless, we have now included additional data from patch-clamp electrophysiological recordings of rat hippocampal neurons (DIV18-20) treated for 48 h with picrotoxin in the revised manuscript **(new Fig. 1B; EV1)**. As previously reported, we observed a significant reduction in mEPSC amplitudes, but not frequencies in PTX- compared to mock-treated neurons. Thus, the neurons used for our study show endogenous activity and respond PTX-treatment with synaptic downscaling.

-Fig. 2: compartment-specific regulation of certain RNAs is a well-accepted concept in the field to explain local expression changes. For the 463 commonly regulated transcripts, do the authors observe differences in splice isoforms and/or 3'-UTR

length? Moreover, how many of these genes are indeed neuron-specific? A more in-depth analysis would be required to provide novel insight.

Since we have already performed a very in-depth characterization of the transcriptome by RNA-seq, we were in a position to further analyze splice isoforms and 3'UTR length differences in a compartment-specific manner using the already available datasets. These new analyses are now provided in **new Fig. EV3**. Briefly, we observed very interesting **cases for compartment-specific regulation of exon and UTR usage upon PTX treatment (e.g. Homer1)**. Thus, this analysis provides a further justification for performing a multiomics characterization of the synaptic downscaling process in a compartment-specific fashion.

-Fig. 4C: The Pearson correlation coefficient of 0.78 indicates a good correlation between RNA and protein levels. Can the authors provide also the Spearman correlation coefficient to demonstrate that their analysis is independent from the test procedure they used?

We thank the reviewer for pointing this out and have **now provided the Spearman correlation coefficient in all main figures** of the manuscript. In general, we did not observer major differences when using Spearman or Pearson coefficients.

Moreover, did the authors include only significantly changed proteins and RNAs?

The data shown in Fig. 4C only includes significantly changed proteins and RNAs. However, since this point was also brought up by reviewer 2 (see below), we now included **additional correlation plots** with all detectably expressed proteins/RNA, as well as synaptic genes/proteins**, i**n the revised manuscript as supplementary figures **(new Fig. EV4)**. Thereby, we also observed positive correlation between RNA/protein fold-changes at the level of all genes, but, as expected, the correlation becomes stronger the more stringent the inclusion criteria are.

From the plot provided, it is currently hard to judge the actual fold change. For some proteins, it looks like that the fold change is indeed very small. In line with that, it seems like that most of the protein alterations are based on RNA level changes, meaning that the contribution of translation is minor. Isn't that in contrast to published papers claiming that (local) translation is one of the main drivers of synaptic scaling (Schanzenbaecher et al. 2016, Neuron). Have the authors already collected data on translation in that respect?

Indeed, it appears that fold-changes observed in proteomics are often smaller compared to those observed by RNA-seq. Although we have currently no experimental evidence, we suppose that the major reason for lower fold-changes of proteins compared to RNAs is their longer half-lives. Therefore, relatively large changes in RNA abundance do not immediately translate into corresponding alterations in protein levels.
It is true that mRNA translation has been identified previously as important contributor to synaptic scaling, but exclusively in the context of **synaptic upscaling** induced by TTX or retinoic acid (e.g. Sutton et al., 2006; Aoto et al., 2008.). In the reference mentioned by the reviewer (Schanzenbächer et al. 2016), transcriptome analysis by RNA-seq was not performed, leaving open the possibility that the

observed protein changes in this study are also, at least in part, due to corresponding changes in mRNA levels. In this regard the authors state in their discussion: "Whether the observed changes in protein synthesis are due to changes in translation specifically or accompanied by changes in transcript level is an interesting question.»
Intriguingly, the vast majority of the proteins known to function at glutamatergic synapses were found to be downregulated during scaling also by Schanzenbächer et al. (e.g. Shanks, Homer, etc.) and Dörrbaum et al. (e.g. Homer1, Add2, Camk2a). Therefore, synaptic downscaling might initially require the new synthesis of so far unknown proteins for its expression, but the downregulation of key synaptic proteins for its maintenance over extended periods of time.
As eluded to in the discussion (p.15), we provide several lines of evidence that the latter involves RNA degradation in the process compartment. Nevertheless, we admit that our approaches (e.g. Puro-PLA; **new Fig. 5**) do not rule out a contribution of reduced mRNA translation efficacy, and these processes are not mutually exclusive. We have now acknowledged this fact in the discussion of the revised manuscript.

Moreover, in their dataset, how much of the RNA level changes are due to transcriptional alterations and how much is caused by RNA decay? Do they see differences in uridylation of the RNAs they detected?

We have now included an **exon/intron split analysis** (EISA, cf. Rajman et al. 2017) to distinguish between transcriptional and post-transcriptional regulation in the revised manuscript (**new Appendix Fig. S4**). However, since RNAs containing intronic reads are rarely observed in the process compartment, EISA is not suitable to analyze PTX effects specifically in the process compartment, which makes it hard to draw definitive conclusions. The fact that we observe many examples, especially of synaptic genes, which display a specific mRNA downregulation in the process compared to the somatic compartment argues for a local post-transcriptional component (i.e. RNA decay), since one would expect a uniform downregulation in both compartments if transcriptional inhibition would be the predominant mechanism. Similarly, transcriptional inhibition as the main mechanism for downregulation in processes was not supported by our mRNA half-life analysis **(new Appendix. Fig. S5)**.
However, to unequivocally assess potential effects on mRNA stability, new approaches to study individual candidates (e.g. TREAT, Horvathova et al., 2017) or global RNA turnover (e.g. 4-thiouridine (4SU) metabolic labeling) would be required. We tried hard to establish TREAT for selected candidates, but were not able to obtain the specificity required to draw definitive conclusions. This is likely due to the fact that TREAT requires the expression of multiple reporter RNAs from large vectors, which so far has only been successfully achieved in the context of cell lines (e.g. HeLa) with stable genomic integration of the vectors. In neurons, transfection of these reporters proved inefficient and in addition resulted in high background signal, likely due to undegraded plasmid DNA attaching to the neuronal surface. The large vector size further precluded packaging into viral particles for more efficient delivery. Further optimization of the protocol is currently ongoing.
4SU metabolic labeling to our knowledge has so far not been established in the context of compartmentalized primary neuron cultures where starting material is very limiting. Taking into account our experience with bulk RNA-seq in this model, we

expect that major efforts are needed to adopt 4SU to compartmentalized cultures, which in our view is beyond the scope of the present study.

-Fig. 5: I see the point why including RBPs and miRNAs. However, without functional data, this analysis is very descriptive and contains little information.
As mentioned before, we believe that in the context of this resource article, a detailed functional analysis of the role of specific RBPs and miRNAs is beyond the scope. Our article constitutes an important resource for detailed mechanistic follow-up studies by us and other colleagues in the future.

Reviewer #1 (Significance (Required)):

As outlined above, the current manuscript fails to distinguish itself from published papers in terms of novelty. Furthermore, the authors have to compare their data with the mentioned papers to clearly work out what are common findings and what are main differences. Especially, the potential impact of translation has to be investigated and discussed in thorough detail.
Please see our responses above related to the individual concerns.

Reviewer #2 (Evidence, reproducibility and clarity (Required)):

Colameo et al. study the transcriptome/proteome changes underlying synaptic scaling. In addition to previous reports from the same lab they now focus on different neuronal compartments, namely they employ an established model for synaptic scaling and then analyze the "somatic" vs the "process" compartment. This is possible since they grow hippocampal neurons in trans-well plates. They observe a number of interesting changes and suggest a number of processes that may play a role in local transcriptome/proteome changes during synaptic scaling.

The importance of the study does not depend so much on novel mechanistic insights but it provides a very important resource to the field. In fact, the analysis of localized regulation of mRNA/protein abundance in neurons is a very timely field of research but - at least in my view - the published data is not yet sufficient and sometimes conflicting, which may also be due to the fact that researchers study synaptosomes or synapto-dendritic compartments using different technical approaches. The described data and the available raw data will therefore be a powerful resource for future studies.
We are happy that this reviewer feels that our research is timely and provides a powerful resource for the field and future studies.

Having this said, some of the analysis might be further improved. An example might be the efforts to correlate the transcriptome to the proteomic changes, which so far only refers to selected genes/proteins in most cases.
We agree that a more detailed correlation of transcriptome and proteome changes is needed, and have now provided this in the revised manuscript (**new Fig. EV4**, see also our comments to reviewer 1). For example, in addition to focusing on significantly changing genes, **we have now also performed a transcriptomic/proteomic correlation on a global level (EV4A)**. Furthermore, **we**

have specifically correlated RNA/protein changes for synaptic genes (EV4B) and obtained further evidence regarding a potential involvement of RNA decay in their PTX-mediated downregulation.

The authors also cite the previous literature that analyzed the synaptic/dendritic transcriptome/proteome. A more thorough comparison of their data under basal conditions might be helpful to the reader to judge and compare the current data and previous studies, at least under basal conditions. That could be a further supplemental figure
This is also in agreement to the point raised by Reviewer 1 and was addressed with additional bioinformatics analysis in the revised manuscript.
For comparison of the basal data (without PTX, just at the compartment level), we focused on a recent meta-analysis (Kügelen & Chekulaeva, 2020, RNA), since this study provides the most comprehensive data on neurite-enriched transcripts **(new Fig. EV2).** These new analyses revealed a strong correlation between our and published datasets under basal conditions, in particular when restricting the analysis to genes that show high overall expression levels in dendrites (EV2E). Therefore, we are confident that our model system faithfully recapitulates mRNA localization to neuronal processes.

Another minor issues that might be checked is relate to editing. e.g.; panel C is not indicated in Fig 3
We have now fixed this editing issue in the revised manuscript.

Reviewer #2 (Significance (Required)):

The study provides a very important resource to the field and the data will be of interest for future studies that aim to analyze local RNA/Protein dynamics in neurons.

Reviewer #3 (Evidence, reproducibility and clarity (Required)):

Summary:

The authors show the changes in the RNA and protein caused by the treatment of PTX and a supposed synaptic downscaling. They use an in vitro model of rat hippocampal neurons which implies a filter insert that separates processes from soma allowing only growing of neuronal processes to the lower side of the insert. They first dissect transcriptomic changes in the different compartments: the processes and the soma, and then, they also assess that most of the changes also occur at the protein level. These transcriptomic changes are 50% compartment-specific, meaning that 50% of this RNA only changes in the processes or the soma. Finally, they investigate the cause of this compartment-specific changes and they reveal that post-transcriptional changes induced by RNA-binding proteins (RBPs) among others govern this phenomenon, and that this specificity is given by the precise sequence motifs located within the 3'UTR.

Major comments:

-Are the key conclusions convincing?

They don't demonstrate the synaptic downscaling in their model, although it has been described, it should be analysed at least and presented in a supplementary figure.

As already mentioned in response to reviewer 1, we have now included an electrophysiological characterization of our synaptic downscaling model (48h picrotoxin treatment of primary hippocampal neurons) in the revised manuscript **(new Fig. 1B; Fig. EV1).** We observed the expected reduction in mEPSC amplitude, but not frequency, thereby confirming that our pharmacological approach led to robust downscaling of excitatory synapse of hippocampal pyramidal neurons.

It is also not well described and not demonstrated that the changes are widespread and not only local in the synapses.

We apologize if our statements regarding widespread compartment-specific changes in gene expression were ambiguous. In fact, we wanted to convey the message that local changes (either happening predominantly in the process or somatic compartment) are not the exception, meaning that there are rather large gene sets (>500) which respond to chronically elevated activity in a highly compartment-specific manner, presumably due to the existence of local post-transcriptional mechanisms involving RBPs and microRNAs. In addition, there are of course many genes, as observed in multiple previous studies (e.g. Dörrbaum et al. 2020; Schanzenbächer et al. 2016, 2018; Rajman et al., 2017), which respond to PTX at the level of the entire neuron. We try to better explain these observations in the revised manuscript and have toned down our statements regarding widespread expression in the abstract and the discussion.

-Should the authors qualify some of their claims as preliminary or speculative, or remove them altogether?

Yes, they say that their study indicates that compartmentalized gene expression changes are widespread in synaptic scaling and might co-exist with neuron-wide mechanisms to allow synaptic computation and homeostasis but they don't demonstrate that the gene expression changes are widespread. There are some things not coherent, such as for example that 50% of the DEGs were common to both compartments and that they say that a large fraction of PTX-responsive RNAs displays compartment-specific regulation- This sentence is ambiguous

Please see our response to the previous concern raised by this reviewer above.

They shouldn't say that the changes of the RNA and protein have a high reproducibility in the control conditions, as they don't measure the correlation as they do in the PTX vs Mock conditions.

We apologize for this misunderstanding, but our statement (p.5) actually only referred to the reproducibility of proteomics and transcriptomics experiments separately. In fact, we were not able to perform a correlation of RNA and protein

levels under basal conditions, since we do not have a reference point for this kind of analysis (such as the mock-treated condition for the PTX analysis).

They should specify which is the percentage of matches of RNA-protein changes.

We have now provided these numbers in the **new Appendix figure S9** in the revised manuscript.

Then they also say that the protein and RNA changes correlate but that compart specific localization relies on post-transcriptional mechanisms.

We have now included additional data (EISA, Analysis based on RNA half-lives, **new Appendix Fig. S4, S5**) which suggests that compartment-specific regulation is involved post-transcriptional mechanisms. However, as pointed out already in our response to reviewer 1, our experimental setup does not allow us to unequivocally rule out a contribution of transcriptional control to the observed PTX-dependent alterations in gene expression. This has now been clarified in the discussion.

-Would additional experiments be essential to support the claims of the paper? Request additional experiments only where necessary for the paper as it is, and do not ask authors to open new line of experimentation.

I suggest performing two kind of experiments, the first one in order to visualize that RNA translation of some of the DEGs is ocurrying differently in PTX and Mock conditions as they mention in the discussion; I will use assays that allow a direct visualization of local mRNA translation (e.g. Puro-PLA38) or local mRNA degradation (e.g. TREAT39) because they would be more aclarative and would be a proof that the mRNA are being translated or degraded locally.
We want to thank this reviewer for these excellent suggestions. Unfortunately, we were unable to establish the TREAT assay due to technical obstacles (see also our comments to reviewer 2). However, we **successfully established Puro-PLA**, which allowed us to monitor local protein synthesis of candidates in PTX-treated hippocampal neurons (**new Fig. 5**). Thereby, we were able to show PTX-dependent downregulation of Camk2a and Syn1 expression in the process compartment, consistent with our results from RNA-seq and proteomics. Together, this suggests that PTX reduces the de novo synthesis of important synaptic proteins locally in processes. As pointed out in the discussion, the exact mechanism of this inhibition is not clear, but based on our results likely involves downregulation of the corresponding mRNAs, e.g. via RBPs and microRNAs. In addition, a reduction in mRNA translation efficacy might also be involved (see our discussion on p.15).
The second one: experiments with organotypic cultures in which you maintain the normal synaptic connections in a more physiological way and would try to assess the change in expression after treatment with PTX of some specific RNAs that change in the soma or in the processes will clarify that the transcriptomic changes that occur in the model they define in the article can occur also in a more physiological way, as the in the vitro model you have the processes completely separated from the somas and this doesn't occur in the same way in vivo.
We agree that organotypic slice cultures would constitute a more physiological way to look at synaptic downscaling, although they still represent an *ex vivo* system

which doesn’t fully recapitulate the situation in the intact hippocampus. The difference to cultured neurons is also not as pronounced as assumed by this reviewer, since processes are still intact and attached to the soma in the filter insert model (severing of processes occurs after the 48h PTX treatment during lysate preparation).
Most importantly, organotypic cultures are currently not established in our laboratory, and we also do not have any colleagues nearby who could have helped us with setting them up in a timeframe compatible with this revision. Altogether, considering that the potential added value obtained with the organotypic cultures is rather small, we feel that the cost-benefit ratio is not favorable and therefore refrained from establishing this model.
-Are the suggested experiments realistic in terms of time and resources? It would help if you could add an estimated cost and time investment for substantial experiments.

Regarding the first experiment is realistic in terms of time and resources because it can be done in 2-3 months, and in terms of resources they just have to acquire the plasmids.
The second one could me more time-consuming if they don't have a well stablish organotypic culture model and it would cost more resources because they might have to acquire new reagents or products.
We agree with these statements and therefore decided to pursue Puro-PLA assays but not experiments using organotypic slices.

-Are the data and the methods presented in such a way that they can be reproduced?

The methods used for the experiments are accurately explained in a way that can be reproduced.

-Are the experiments adequately replicated and statistical analysis adequate?

Regarding the statistical analysis, they test for normality and use the specific adequate test for the different experiments.

Minor comments:

-Specific experimental issues that are easily addressable.

-Are prior studies referenced appropriately?

The prior studies are referenced appropriately

-Are the text and figures clear and accurate?

The figure 5 schemas need to be finsihed.
The figure 5E legend needs to be clarified.
It lacks a C nomenclature for figure 3C.

We have modified the figures according to these suggestions in the revised manuscript.

-Do you have suggestions that would help the authors improve the presentation of their data and conclusions?

It would help a wider explanation in the introduction of the synaptic upscaling/downscaling processes for people not totally involved in this field. They also need to review the sentences that are ambiguous and change the conclusions as I have mentioned above.

As suggested by this reviewer, we have expanded the introduction (p.3), fixed ambiguities and toned down some of the conclusions which are not fully supported by the current dataset. In particular, a more extensive discussion regarding the potential mechanisms underlying the observed compartment-specific regulation of synaptic mRNAs has been added (p.15).

Reviewer #3 (Significance (Required)):

-Describe the nature and significance of the advance (e.g. conceptual, technical, clinical) for the field.

The paper might be of interest for people working in the field.

-Place the work in the context of the existing literature (provide references, where appropriate).

There are studies revealing some specific mechanisms about how synaptic scaling is working, such as the retinoic acid signalling which mediates synaptic downscaling1 other that show that synaptic scaling involves changes in locally translated RNAs2, but a wide genomic study was lacking. This work gives a wide information of all the RNAs changed upon PTX treatment induced synaptic downscaling and how these changes are regulated to be compartment specific.
We are grateful to this reviewer that s/he points out that a wide genomic study looking at compartment-specific changes in gene expression during synaptic scaling was lacking.

1. Aoto, J., Nam, C. I., Poon, M. M., Ting, P. and Chen, L. Synaptic signaling by all-trans retinoic acid in homeostatic synaptic plasticity. Neuron 60, 308-320, doi: S0896-6273(08)00707-1 [pii]

2. Sun, C., Nold, A., Tchumatchenko, T., Heilemann, M., and Schuman, EM. The Spatial Scale of Synaptic Protein Allocation during Homeostatic Plasticity (BioRxiv, doi: https://doi.org/10.1101/2020.04.29.06883).

-State what audience might be interested in and influenced by the reported findings.

The audience might be interested in the RNA-seq data generated in the study

because it can open new studies about new molecules functioning in synaptic downscaling or in synaptogenesis. It can also be useful to study the etiology of different diseases which include synaptic down/upscaling due to an excess or a decrease of activity such as Epilepsy, Alzheimer Disease, etc.

-Define your field of expertise with a few keywords to help the authors contextualize your point of view. Indicate if there are any parts of the paper that you do not have sufficient expertise to evaluate.

My field of expertise is synaptogenesis, neural circuits development and early embryogenesis.

1st Revision - Editorial Decision 23rd Jun 2021

Dear Gerhard,

Thank you for the submission of your revised manuscript. We have finally received the enclosed reports from all referees. As you will see, while referee 1 is more negative, referee 2 (in the cross-comments) and referee 3 agree that your manuscript will be a useful resource for the field, so we can offer to publish it as a resource.

Please do address referee 1's minor comments and the comments on the figures and referee 3's comments in a point-by-point response and incorporate the changes in the final manuscript file.

A few other editorial changes will also be required:

- Please reduce the number of keywords to 5.

- Please rename "Competing Interests" to Conflict of Interest.

- Please correct the reference style to the correct EMBO reports style (slightly different from the current style): https://www.embopress.org/page/journal/14693178/authorguide#referencesformat

- Fig 1F callout is missing. Figs. EV1-EV4 panel callouts are missing.
The Appendix figure panels are not called out. There are a couple of callout to "Suppl Fig.", please correct.

- The Appendix file is missing a table of content with page numbers. The nomenclature is incorrect, the figures need to be called Appendix Figure S1, etc.

- The subheading "Methods" needs to be corrected to "Materials and Methods".

- Please add the subheading 'Expanded View Figure Legends'.

- The "Real time PCR primers" should be moved to the paragraph on real time PCR in the methods section.

- The Data Availability Section needs to be moved to the end of the Materials and Methods section.

I attach to this email a related ms file with comments by our data editors. Please address all comments in the final manuscript.

EMBO press papers are accompanied online by A) a short (1-2 sentences) summary of the findings and their significance, B) 2-3 bullet points highlighting key results and C) a synopsis image that is exactly 550 pixels wide and 200-600 pixels high (the height is variable). You can either show a model or key data in the synopsis image. Please note that text needs to be readable at the final size. Please send us this information along with the revised manuscript.

I made a few changes to the abstract that needs to be written in present tense:

Synaptic scaling is a form of homeostatic plasticity which allows neurons to adjust their action potential firing rate in response to chronic alterations in neural activity. Synaptic scaling requires

profound changes in gene expression, but the relative contribution of local and cell-wide mechanisms is controversial. Here we perform a comprehensive multi-omics characterization of the somatic and process compartments of primary rat hippocampal neurons during synaptic scaling. We uncover both highly compartment-specific and correlating changes in the neuronal transcriptome and proteome. Whereas downregulation of crucial regulators of neuronal excitability occurs primarily in the somatic compartment, structural components of excitatory postsynapses are mostly downregulated in processes. Local inhibition of protein synthesis in processes during scaling is confirmed for candidate synaptic proteins. Motif analysis further suggests an important role for trans-acting post-transcriptional regulators, including RNA-binding proteins and microRNAs, in the local regulation of the corresponding mRNAs. Altogether, our study indicates that, during synaptic scaling, compartmentalized gene expression changes might co-exist with neuron-wide mechanisms to allow synaptic computation and homeostasis.

Please let me know whether you agree with these changes.

I look forward to seeing a final version of your manuscript as soon as possible. Please use this link to submit your revision: https://embor.msubmit.net/cgi-bin/main.plex

Best wishes,
Esther

Esther Schnapp, PhD
Senior Editor
EMBO reports

Referee #1:

In the current study, Colameo, Schratt et al. extended their own previous studies on homeostatic synaptic scaling. Here they exploited a multi-omics approach using RNA-seq and mass spectrometry. Whereas the mentioned papers by the Schuman lab did not use a filter method to explicitly separate processes from cell bodies, they provide convincing data for both the pre- and the postsynaptic compartments (Schanzenbacher 2016, 2018 amongst others). Also, these studies did not do mass spec and RNAseq simultaneously. However, those papers provided the first interesting data on changes in the proteome of the two compartments. As outlined in my initial review, I thought (and I am still convinced) that the authors should provide functional and/or mechanistic insight into the process of homeostatic synaptic scaling. Here are plenty of options, e.g. experiments addressing RNA decay, translational regulation, RBPs or miRNAs.
In the revised manuscript, the authors have now added some electrophysiology (new Fig. 1B, Fig. EV1) reporting "the expected reduction in mEPSC amplitude, but not frequency, thereby confirming that our pharmacological approach led to robust downscaling of excitatory synapse of hippocampal pyramidal neurons". In my opinion, this confirms that their picrotoxin model works.
Second, Schratt and colleagues followed the suggestion to compare the datasets from Schanzenbacher et al. (2016, 2018) and Dorrbaum et al. (2020) to investigate changes at the proteomic level (new Fig. EV5). Here, they confirm a good correlation with the mentioned studies at the protein level. Furthermore, they see a stronger correlation with their somatic data set than with their process dataset and increased resolution. However, one would like to see functional data on the mentioned downregulated genes (Ccar1, Khsrp, Ncbp1).
Third, the authors included bioinformatics analysis that RBP and miRNA regulation might be

involved in gene expression in different compartments. However, this initial data sets needs experimental validation.
Fourth, the authors show first data for splice isoforms and 3'UTR length differences in Fig. EV3.
In conclusion, the manuscript does not per se qualify as resource as it fails to significantly distinguish itself from published ones. Furthermore, recent published resource papers provide validation data to convince the general readership that the approach is of general interest.
For a research paper, the study does not (yet) qualify due to a lack in functional new insight into the underlying process of homeostatic synaptic scaling. Finally, the figures are not publication quality, they are not properly labeled/numbered, several figures are cut/incomplete, e.g. Figs 4, 9, 10, and the last. Significantly more emphasis has to be put on presenting data in the optimal way for general readership.

Minor point:
Intro, p4: "However, recent theoretical considerations challenged this view and suggested an important contribution of local mechanisms (e.g. operating at the level of individual dendritic segments) to synaptic scaling (Rabinowitch and Segev, 2008). Well, you mean 13 years ago? In my opinion, this evidence is not recent and goes back even longer.

Referee #2:

The new version of the manuscript has benefit from addressing referees' concerns/comments. It now adds new data and analysis that further improve the significance of the results. In my opinion, the manuscript is suited to be published in the current form.

Referee #3:

Colameo et al. employ hippocampal primary cultures to study the mechanisms induced during synaptic scaling. As a model for synaptic scaling they treat neurons with picrotoxin (PTX). The novelty of the study lies in the fact that neurons are grown in a transwell system and that the authors study the proteome and RNAome in response to PTX treatment in specific compartments such as neurites. Using this system they describe a number of mRNA and protein changes upon PTX treatment. These data represent an important resource to the field. My task was to evaluate the current revision of the manuscript on the basis of the other reviewer's comments. In my view the authors did a solid job to address the remaining questions and especially the PLA experiments add to the insight. It is true that the study remains descriptive but I would not follow the arguments of reviewer 1 that the data would not be novel. I believe that data will be a valuable resource to the field.

More specific comments

- Do the cultures include glia cells? Why does cell cycle genes are induced in processes? Page 7.

- Fig3A &C. Rather then showing the FC only, it might be more informative to also show the actual control groups and compare. Thus, please also compare to control and not just somata vs processes.

- Fig 3B. The analysis seems to focus on "processes" regions close to the soma. Would the findings

be similar if regions more distant to the some are analyzed? This should at least be discussed.

- The microRNA data is interesting but should ideally be supported by experiments or at least a comparison to other datasets reporting synaptic/somatic miRNAs such as PMID 31097639 or 33569760

Cross-comments Referee 2:

I agree with most of the comments of the referee #1. However, as mentioned by this referee, none of the previous publications provided i) separated cell body and processes analysis and ii) simultaneous mass spec and RNAseq data, which, in my opinion, distinguishes this work from the previous ones (novelty).

The manuscript would benefit from validation data, of course, but as also pointed out by referee #1, there is a high correlation of the somatic data with previous publications, which, again, in my opinion, validates their approach and datasets.

Finally, although some recent resource publications provide validation data, it is not always the case. The manuscript does not suggest a paradigm shift or new concepts that would require further experimental validation. As I previously indicated, I understand the manuscript as a resource, with useful data, not outstanding, but of interest for people in the field (not as a research article which will need a strong validation support).

In sum, I did not have the feeling to be in front of a particularly striking manuscript, but a correct one, with useful data for the community.

EMBOR-2020-52094V2 – Point-by-Point Response

Reviewer 1
Minor point:

Intro, p4: "However, recent theoretical considerations challenged this view and suggested an important contribution of local mechanisms (e.g. operating at the level of individual dendritic segments) to synaptic scaling (Rabinowitch and Segev, 2008). Well, you mean 13 years ago? In my opinion, this evidence is not recent and goes back even longer.

We agree with this reviewer that the theoretical evidence pointing to a contribution of local mechanisms in synaptic scaling is not recent. We have changed the wording in the revised manuscript accordingly.

Reviewer 3
More specific points:

- Do the cultures include glia cells? Why does cell cycle genes are induced in processes? Page 7.

We used «mixed cultures» consisting of neurons and glia cells for our experiments. The presence of glia cells is in fact very important to support proper synapse maturation in these cultures. Therefore, it is also not surprising that we found an induction of cell cycle genes in processes, which likely originates from abundant glia processes present in the process compartment.

- Fig3A &C. Rather then showing the FC only, it might be more informative to also show the actual control groups and compare. Thus, please also compare to control and not just somata vs processes.

We completely agree with this comment and now also provide a comparison between PTX- and control-treated neurons for FISH and qPCR analysis in the Appendix (new Appendix Fig. S7).

- Fig 3B. The analysis seems to focus on "processes" regions close to the soma. Would the findings be similar if regions more distant to the some are analyzed? This should at least be discussed.

We apologize for being unclear here, but the analysis was in fact performed on the entire dendrite area. The boxes shown in Fig. 3B simply depict the regions which are shown at higher magnification on the right panel of the respective figure.

- The microRNA data is interesting but should ideally be supported by experiments or at least a comparison to other datasets reporting synaptic/somatic miRNAs such as PMID 31097639 or 33569760

As already pointed out in our previous response letters, we strongly agree that an in-depth functional characterization of microRNAs originating from our bioinformatics analysis is beyond the scope of this resource article. As suggested by this reviewer, we have now compared our dataset (Fig. 7F) to a recent study from the Fischer lab (PMID 33569760, Fig. 1F; No microRNA expression data is provided in PMID 31097639). Intriguingly, many of the candidate miRNAs are shared between these two studies (e.g. miR-24-3p, miR-132-3p, miR-125a-5p, miR-129-5p), providing further support for a role of these miRNAs in the regulation of local mRNA translation at synapses. We have added a sentence summarizing these results to the discussion (p. 16).

2nd Revision - Editorial Decision 20th Jul 2021

Prof. Gerhard Schratt
ETH Zurich - D-HEST
Systems Neuroscience, Bldg Y17 L48
Institute for Neuroscience
Winterthurerstr. 190
Zurich, Zurich 8057
Switzerland

Dear Gerhard,

I am very pleased to accept your manuscript for publication in the next available issue of EMBO reports. Thank you for your contribution to our journal.

At the end of this email I include important information about how to proceed. Please ensure that you take the time to read the information and complete and return the necessary forms to allow us to publish your manuscript as quickly as possible.

As part of the EMBO publication's Transparent Editorial Process, EMBO reports publishes online a Review Process File to accompany accepted manuscripts. As you are aware, this File will be published in conjunction with your paper and will include the referee reports, your point-by-point response and all pertinent correspondence relating to the manuscript.

If you do NOT want this File to be published, please inform the editorial office within 2 days, if you have not done so already, otherwise the File will be published by default [contact: emboreports@embo.org]. If you do opt out, the Review Process File link will point to the following statement: "No Review Process File is available with this article, as the authors have chosen not to make the review process public in this case."

Should you be planning a Press Release on your article, please get in contact with emboreports@wiley.com as early as possible, in order to coordinate publication and release dates.

Thank you again for your contribution to EMBO reports and congratulations on a successful publication. Please consider us again in the future for your most exciting work.

Best wishes,
Esther

Esther Schnapp, PhD
Senior Editor
EMBO reports

****

THINGS TO DO NOW:

You will receive proofs by e-mail approximately 2-3 weeks after all relevant files have been sent to our Production Office; you should return your corrections within 2 days of receiving the proofs.

Please inform us if there is likely to be any difficulty in reaching you at the above address at that time. Failure to meet our deadlines may result in a delay of publication, or publication without your corrections.

All further communications concerning your paper should quote reference number EMBOR-2020-52094V3 and be addressed to emboreports@wiley.com.

Should you be planning a Press Release on your article, please get in contact with emboreports@wiley.com as early as possible, in order to coordinate publication and release dates.

EMBO PRESS

YOU MUST COMPLETE ALL CELLS WITH A PINK BACKGROUND

PLEASE NOTE THAT THIS CHECKLIST WILL BE PUBLISHED ALONGSIDE YOUR PAPER

Corresponding Author Name: Gerhard Schratt
Journal Submitted to: EMBO Reports
Manuscript Number: EMBOR-2020-52094V1

Reporting Checklist For Life Sciences Articles (Rev. June 2017)

This checklist is used to ensure good reporting standards and to improve the reproducibility of published results. These guidelines are consistent with the Principles and Guidelines for Reporting Preclinical Research issued by the NIH in 2014. Please follow the journal's authorship guidelines in preparing your manuscript.

A- Figures
1. Data
The data shown in figures should satisfy the following conditions:

- the data were obtained and processed according to the field's best practice and are presented to reflect the results of the experiments in an accurate and unbiased manner.
- figure panels include only data points, measurements or observations that can be compared to each other in a scientifically meaningful way.
- graphs include clearly labeled error bars for independent experiments and sample sizes. Unless justified, error bars should not be shown for technical replicates.
- if n< 5, the individual data points from each experiment should be plotted and any statistical test employed should be justified
- Source Data should be included to report the data underlying graphs. Please follow the guidelines set out in the author ship guidelines on Data Presentation.

2. Captions

Each figure caption should contain the following information, for each panel where they are relevant:

- a specification of the experimental system investigated (eg cell line, species name).
- the assay(s) and method(s) used to carry out the reported observations and measurements
- an explicit mention of the biological and chemical entity(ies) that are being measured.
- an explicit mention of the biological and chemical entity(ies) that are altered/varied/perturbed in a controlled manner.

- the exact sample size (n) for each experimental group/condition, given as a number, not a range;
- a description of the sample collection allowing the reader to understand whether the samples represent technical or biological replicates (including how many animals, litters, cultures, etc.).
- a statement of how many times the experiment shown was independently replicated in the laboratory.
- definitions of statistical methods and measures:
  - common tests, such as t-test (please specify whether paired vs. unpaired), simple χ2 tests, Wilcoxon and Mann-Whitney tests, can be unambiguously identified by name only, but more complex techniques should be described in the methods section;
  - are tests one-sided or two-sided?
  - are there adjustments for multiple comparisons?
  - exact statistical test results, e.g., P values = x but not P values < x;
  - definition of 'center values' as median or average;
  - definition of error bars as s.d. or s.e.m.

Any descriptions too long for the figure legend should be included in the methods section and/or with the source data.

In the pink boxes below, please ensure that the answers to the following questions are reported in the manuscript itself. Every question should be answered. If the question is not relevant to your research, please write NA (non applicable). We encourage you to include a specific subsection in the methods section for statistics, reagents, animal models and human subjects.

USEFUL LINKS FOR COMPLETING THIS FORM

http://www.antibodypedia.com
http://1degreebio.org
http://www.equator-network.org/reporting-guidelines/improving-bioscience-research-repor
http://grants.nih.gov/grants/olaw/olaw.htm
http://www.mrc.ac.uk/Ourresearch/Ethicsresearchguidance/Useofanimals/index.htm
http://ClinicalTrials.gov
http://www.consort-statement.org
http://www.consort-statement.org/checklists/view/32-consort/66-title
http://www.equator-network.org/reporting-guidelines/reporting-recommendations-for-tumo
http://datadryad.org
http://figshare.com
http://www.ncbi.nlm.nih.gov/gap
http://www.ebi.ac.uk/ega
http://biomodels.net/
http://biomodels.net/miriam/
http://jjj.biochem.sun.ac.za
http://oba.od.nih.gov/biosecurity/biosecurity_documents.html
http://www.selectagents.gov/

B- Statistics and general methods

	Please fill out these boxes (Do not worry if you cannot see all your text once you press return)
1.a. How was the sample size chosen to ensure adequate power to detect a pre-specified effect size?	Samples sizes were determined based on the expected effect sizes and variability.
1.b. For animal studies, include a statement about sample size estimate even if no statistical methods were used.	NA
2. Describe inclusion/exclusion criteria if samples or animals were excluded from the analysis. Were the criteria pre-established?	No data was excluded from the results, except for a strong outlier in the qPCR data (Shank2 experiment 3 as it showed an extreme increase, outlier removal was justified by the value being bigger than 1.5 times the interquantile range (IQR as difference between the 25th and 75th quantiles)
3. Were any steps taken to minimize the effects of subjective bias when allocating animals/samples to treatment (e.g. randomization procedure)? If yes, please describe.	For FISH-microscopy experiments, researchers were blinded to the experimental condition. For Duolink-Puro-PLA experiments, researchers were blinded to the Duolink channel-signal but not to experimental condition but MAP2-channel was used as sole criterion for cell selection.
For animal studies, include a statement about randomization even if no randomization was used.	For studies involving cell culture, randomization of the samples is not necessary.
4.a. Were any steps taken to minimize the effects of subjective bias during group allocation or/and when assessing results (e.g. blinding of the investigator)? If yes please describe.	Image Analysis was completely automated, thus unbiased.
4.b. For animal studies, include a statement about blinding even if no blinding was done	For studies involving cell culture, randomization of the samples is not necessary.
5. For every figure, are statistical tests justified as appropriate?	Yes.
Do the data meet the assumptions of the tests (e.g., normal distribution)? Describe any methods used to assess it.	Normality was tested using the Shapiro-Wilk test considering a p-value under 0.05 non-normally distributed. Normally distributed data was tested using one- or two sample Student's T-test (always two-sided), ANOVA and otherwise for non-normal data the non-parametric counterpart tests Mann-Whitney-U test or Kruskal-Wallis test. Bivariate correlation analysis between two variables was evaluated using the non-parametric Spearman rank correlation or the parametric Pearson's correlation using the function ggscatter (from ggpubr).
Is there an estimate of variation within each group of data?	We provided 95% confidence intervals (CI) for all tests.

Is the variance similar between the groups that are being statistically compared?	We tested homogeneity of variance using Levene's test where a p-value < 0.05 indicates statistically different variances across groups. In all the ANOVA-models used, our data passed the Levene's test (p-value > 0.05)

C- Reagents

6. To show that antibodies were profiled for use in the system under study (assay and species), provide a citation, catalog number and/or clone number, supplementary information or reference to an antibody validation profile. e.g., Antibodypedia (see link list at top right), 1DegreeBio (see link list at top right).	We provided RRIDs, catalog numbers and clone number (where applicable) for every used antibody in the method section.
7. Identify the source of cell lines and report if they were recently authenticated (e.g., by STR profiling) and tested for mycoplasma contamination.	We used primary rat hippocampal neurons.

* for all hyperlinks, please see the table at the top right of the document

D- Animal Models

8. Report species, strain, gender, age of animals and genetic modification status where applicable. Please detail housing and husbandry conditions and the source of animals.	For the preparation of primary hippocampal neurons, rat embryos (E18), both sexes, wild-type Sprague-Dawley (Janvier) were used.
9. For experiments involving live vertebrates, include a statement of compliance with ethical regulations and identify the committee(s) approving the experiments.	All animal experiments were carried out under institutional guidelines (ZH196/17 Kanton Zürich Gesundheitsdirektion Veterinäramt).
10. We recommend consulting the ARRIVE guidelines (see link list at top right) (PLoS Biol. 8(6), e1000412, 2010) to ensure that other relevant aspects of animal studies are adequately reported. See author guidelines, under 'Reporting Guidelines'. See also: NIH (see link list at top right) and MRC (see link list at top right) recommendations. Please confirm compliance.	compliance with ARRIVE confirmed

E- Human Subjects

11. Identify the committee(s) approving the study protocol.	NA
12. Include a statement confirming that informed consent was obtained from all subjects and that the experiments conformed to the principles set out in the WMA Declaration of Helsinki and the Department of Health and Human Services Belmont Report.	NA
13. For publication of patient photos, include a statement confirming that consent to publish was obtained.	NA
14. Report any restrictions on the availability (and/or on the use) of human data or samples.	NA
15. Report the clinical trial registration number (at ClinicalTrials.gov or equivalent), where applicable.	NA
16. For phase II and III randomized controlled trials, please refer to the CONSORT flow diagram (see link list at top right) and submit the CONSORT checklist (see link list at top right) with your submission. See author guidelines, under 'Reporting Guidelines'. Please confirm you have submitted this list.	NA
17. For tumor marker prognostic studies, we recommend that you follow the REMARK reporting guidelines (see link list at top right). See author guidelines, under 'Reporting Guidelines'. Please confirm you have followed these guidelines.	NA

F- Data Accessibility

18: Provide a "Data Availability" section at the end of the Materials & Methods, listing the accession codes for data generated in this study and deposited in a public database (e.g. RNA-Seq data: Gene Expression Omnibus GSE39462, Proteomics data: PRIDE PXD000208 etc.) Please refer to our author guidelines for 'Data Deposition'.<br><br>Data deposition in a public repository is mandatory for:<br>a. Protein, DNA and RNA sequences<br>b. Macromolecular structures<br>c. Crystallographic data for small molecules<br>d. Functional genomics data<br>e. Proteomics and molecular interactions	We provided all accession numbers in the Data Availability. RNA-Seq Data: Gene Expression Omnibus (GSE155540). Proteomics Data: ProteomeXchange Consortium via the PRIDE partner repository (PXD020745).
19. Deposition is strongly recommended for any datasets that are central and integral to the study; please consider the journal's data policy. If no structured public repository exists for a given data type, we encourage the provision of datasets in the manuscript as a Supplementary Document (see author guidelines under 'Expanded View' or in unstructured repositories such as Dryad (see link list at top right) or Figshare (see link list at top right).	We created a ShinyApp with all processed omics-data as tables and possibility to plot them: https://dcolamethins.shinyapps.io/Compartment_PTX_App/
20. Access to human clinical and genomic datasets should be provided with as few restrictions as possible while respecting ethical obligations to the patients and relevant medical and legal issues. If practically possible and compatible with the individual consent agreement used in the study, such data should be deposited in one of the major public access-controlled repositories such as dbGAP (see link list at top right) or EGA (see link list at top right).	NA
21. Computational models that are central and integral to a study should be shared without restrictions and provided in a machine-readable form. The relevant accession numbers or links should be provided. When possible, standardized format (SBML, CellML) should be used instead of scripts (e.g. MATLAB). Authors are strongly encouraged to follow the MIRIAM guidelines (see link list at top right) and deposit their model in a public database such as Biomodels (see link list at top right) or JWS Online (see link list at top right). If computer source code is provided with the paper, it should be deposited in a public repository or included in supplementary information.	Custom codes are available on request.

G- Dual use research of concern

22. Could your study fall under dual use research restrictions? Please check biosecurity documents (see link list at top right) and list of select agents and toxins (APHIS/CDC) (see link list at top right). According to our biosecurity guidelines, provide a statement only if it could.	No, our study does not fall under dual use research restrictions.
